# Genome-Wide Identification of Alternative Splice Forms Down-Regulated by Nonsense-Mediated mRNA Decay in *Drosophila*

**DOI:** 10.1371/journal.pgen.1000525

**Published:** 2009-06-19

**Authors:** Kasper Daniel Hansen, Liana F. Lareau, Marco Blanchette, Richard E. Green, Qi Meng, Jan Rehwinkel, Fabian L. Gallusser, Elisa Izaurralde, Donald C. Rio, Sandrine Dudoit, Steven E. Brenner

**Affiliations:** 1 Division of Biostatistics, School of Public Health, University of California Berkeley, Berkeley, California, United States of America; 2 Department of Molecular and Cell Biology, University of California Berkeley, Berkeley, California, United States of America; 3 Department of Plant and Microbial Biology, University of California Berkeley, Berkeley, California, United States of America; 4 Stowers Institute for Medical Research, Kansas City, Missouri, United States of America; 5 Max-Planck Institute for Evolutionary Anthropology, Leipzig, Germany; 6 Max-Planck Institute for Developmental Biology, Tuebingen, Germany; Stanford University, United States of America

## Abstract

Alternative mRNA splicing adds a layer of regulation to the expression of thousands of genes in *Drosophila melanogaster*. Not all alternative splicing results in functional protein; it can also yield mRNA isoforms with premature stop codons that are degraded by the nonsense-mediated mRNA decay (NMD) pathway. This coupling of alternative splicing and NMD provides a mechanism for gene regulation that is highly conserved in mammals. NMD is also active in *Drosophila*, but its effect on the repertoire of alternative splice forms has been unknown, as has the mechanism by which it recognizes targets. Here, we have employed a custom splicing-sensitive microarray to globally measure the effect of alternative mRNA processing and NMD on *Drosophila* gene expression. We have developed a new algorithm to infer the expression change of each mRNA isoform of a gene based on the microarray measurements. This method is of general utility for interpreting splicing-sensitive microarrays and high-throughput sequence data. Using this approach, we have identified a high-confidence set of 45 genes where NMD has a differential effect on distinct alternative isoforms, including numerous RNA–binding and ribosomal proteins. Coupled alternative splicing and NMD decrease expression of these genes, which may in turn have a downstream effect on expression of other genes. The NMD–affected genes are enriched for roles in translation and mitosis, perhaps underlying the previously observed role of NMD factors in cell cycle progression. Our results have general implications for understanding the NMD mechanism in fly. Most notably, we found that the NMD–target mRNAs had significantly longer 3′ untranslated regions (UTRs) than the nontarget isoforms of the same genes, supporting a role for 3′ UTR length in the recognition of NMD targets in fly.

## Author Summary

A gene can be processed into multiple mRNAs through alternative splicing. Alternative splicing increases the number of proteins encoded by the genome, but not all alternative mRNAs produce protein. Instead, some are degraded by nonsense-mediated mRNA decay (NMD), a surveillance system that was originally identified as a means of clearing the cell of mRNAs with nonsense, or stop codon, mutations. Alternative splicing that introduces early stop codons will lead to NMD, offering a way for the cell to down-regulate gene expression after a gene has been transcribed. In this paper, we have developed a new analysis method to study the combined effect of alternative splicing and degradation in the fruit fly *Drosophila melanogaster* using microarrays. We have found a stringently defined set of 45 genes that can be spliced either into an mRNA that encodes a protein or into an mRNA that is degraded by NMD, down-regulating the overall gene expression. The affected genes include a number that are central to the cell's regulatory processes, including translation, RNA splicing, and cell cycle progression. Our results also help shed light on how NMD determines whether a stop codon is premature, and thus whether to target an mRNA for degradation.

## Introduction

Nonsense-mediated mRNA decay (NMD) is an RNA surveillance system that down-regulates mRNAs containing early stop codons in all eukaryotes examined [1]. NMD functions to clear the cell of transcripts containing potentially harmful nonsense mutations [2]. In addition to this role in surveillance of mutations, NMD affects the expression of numerous non-mutant endogenous targets [3]–[6]. These natural targets include many mRNAs that are the products of alternative splicing; one study reported that 45% of alternatively spliced human genes have at least one isoform that may be degraded by NMD [7].

In some of these cases, alternative splicing and NMD act together to regulate gene expression, providing an additional layer of post-transcriptional regulation. By altering the abundance and activity of splicing factors, the cell can differentially splice a pre-mRNA into a productive mRNA that encodes a protein or into an unproductive mRNA with an early stop codon that makes the mRNA a target for NMD. Unproductive splicing is used in the regulation and autoregulation of numerous genes [8] including mammalian splicing factors, spliceosome components [9]–[14] and the spermidine/spermine N1-acetyltransferase (SSAT) gene [15].

Alternative splicing is prevalent in the fruit fly *Drosophila*. At least 46% of detected genes show differential expression of alternative regions during development [16]. In flies, alternative splicing plays an important role in many processes including sex determination, neuronal wiring, and eye development [17]–[19]. Although NMD is active in *Drosophila*, our understanding of its impact on the fly transcriptome is limited. A study of the effect of NMD on gene expression in *Drosophila* showed that levels of 14% of detected genes increased at least 1.5-fold after a key NMD factor, UPF1, was depleted [20]. This analysis used gene expression microarrays that assess total mRNA from a gene, and thus it could not measure the levels of distinct alternative splice forms. Natural NMD targets produced by alternative splicing in *Drosophila* have not been assayed previously.

The NMD machinery of *Drosophila*, as in all eukaryotes studied, requires the core set of UPF proteins, UPF1, UPF2, and UPF3 [21],[22]. As in mammals, it also involves SMG1, SMG5, and SMG6 (but, unlike mammals, not SMG7), which are involved in the phosphorylation and dephosphorylation of UPF1 [22]. Although the core NMD machinery is essentially the same in human and *Drosophila*, the mechanism by which premature termination codons are recognized is different in the two organisms. In both cases, the nonsense codon seems to be recognized as premature based on its position relative to proteins associated with the transcript, downstream of the stop codon. In human, the primary downstream markers are exon junction complexes deposited during splicing [23],[24]. Exon junction complexes are not required for NMD in *Drosophila* [22]. A recent study indicates that, instead, some early stop codons are recognized based on their distance from the poly-A tail, mediated by the binding of cytoplasmic poly-A binding protein (PABPC1) [25]. This study provided valuable data about the NMD mechanism based on manipulation of a single reporter construct. Studies of a wider range of NMD targets are necessary before a general rule can be inferred.

Splicing-sensitive microarrays have been used successfully to assay alternative splicing on a global scale (reviewed in [26]). This method has been applied in fly to assess global splicing changes when splicing factors are inhibited or overexpressed and to measure sexually dimorphic splicing [27]–[29]. Microarrays have also been used to measure the effect of NMD on the levels of alternatively spliced mRNAs in human, mouse, worm, and yeast [4],[12],[30],[31]. However, most techniques used to analyze these microarrays only measure the change in probes specific to individual alternative splice junctions or alternative exons. One method, successfully used to assay alternative splicing in human, measures changes in exon inclusion events [32], but has yet to be extended to more general splicing events. None of these methods provide isoform-level fold-changes, limiting their ability to find NMD targets. In this work, we have developed a new algorithm that makes it possible to obtain isoform-level measurements for all categories of alternative splicing and alternative processing events. We use a generative non-linear regression model to deconvolve individual probe measurements into estimates of overall isoform-level fold-changes and relative proportions of isoforms.

Our goals in this project were two-fold: first, to determine the effect of NMD on alternatively spliced mRNAs in the *Drosophila* transcriptome, and second, to identify features of these transcripts that might cause them to be targets of NMD. To assess the effect of NMD, we have inhibited NMD in *Drosophila* cells and measured changes in expression on a custom splicing-sensitive microarray. After measuring junction and exon splicing changes and then estimating isoform-level fold-changes, we identified NMD targets using a hierarchy of stringent criteria that eliminate many secondary effects and potential artifacts, at the cost of substantially reduced sensitivity to legitimate NMD targets. Using this conservative approach, we have found a high-confidence set of 45 genes where NMD decreases the level of one isoform without impacting the levels of other isoforms. We found that the reading frames of NMD–target mRNAs were often misannotated in sequence databases. After identifying the correct reading frames, we found that the NMD–target mRNAs differed significantly from the nontarget isoforms, with shorter coding regions and longer 3′ untranslated regions (UTRs). Our results show that alternative splicing and NMD affect a diverse set of genes in fly including genes involved in translation and mitosis, suggesting that regulation of unproductive splicing might play important roles in *Drosophila*.

## Results

### Microarray analysis of alternative splicing in NMD–inhibited cells

We previously developed a splicing-sensitive microarray to detect alternative splicing, alternative transcription start sites, and alternative polyadenylation in *Drosophila* [27]. The array contains 43,337 exon and junction probes, targeting 7,768 transcripts of 2,793 alternatively processed genes in FlyBase 4. In order to identify cellular mRNAs naturally targeted by the NMD machinery, RNA was obtained from a previous experiment in which levels of the key NMD effectors UPF1 and UPF2 were reduced in S2 cells by dsRNAi, with three independent knockdowns of each effector [20]. Following the functional knockdown of the NMD machinery, confirmed by the stabilization of an NMD reporter, RNA was extracted and the microarray was used to probe the changes in alternative splicing patterns relative to the patterns in control cells treated with an unrelated dsRNA.

When compared with the control samples, the *upf1* knockdown samples showed substantial probe-level changes, as well as substantial down-regulation of probes targeting *upf1* (Figures S1, S2, S3, S4). The *upf2* knockdown showed smaller probe-level changes, and we observed that the probes to the *upf2* gene itself showed only a small decrease in the *upf2* knockdown compared to control, with the exception of one highly up-regulated probe targeting the same area as the dsRNA. This indicates that the *upf2* knockdown was less effective. We have therefore excluded the *upf2* results from our primary analysis; further data are available in the Supplementary Results in Text S1.

### An algorithm to resolve isoform-level changes in expression

Splicing-sensitive arrays that contain splice junction probes can easily measure the change in the use of a given splice junction. However, to study the effect of NMD on mRNA stability, we must know the fold-change of the entire set of isoforms, which may include multiple alternatively spliced junctions. This is not trivial because many of the probes on the array target multiple transcripts. We have developed a new algorithm, based on a generative non-linear regression model with least squares estimation, to deconvolve the measurements of multiple probes targeting different combinations of isoforms into an overall fold-change measurement for each isoform. In addition to isoform fold-changes, the algorithm yields estimates of the relative proportions of the different isoforms.

**Figure 1:**
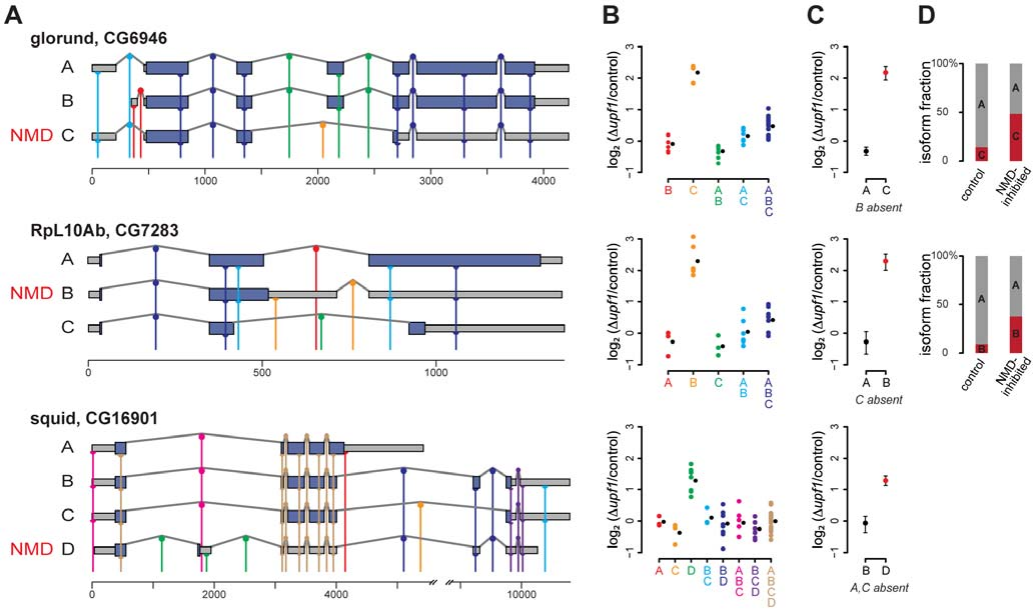
Isoform deconvolution. (A) Probe placement and gene structure for 3 NMD–affected genes: glorund, RpL10Ab, and squid. Gene structures are shown with exons as boxes and introns represented by peaked lines. Dark blue regions indicate the coding region and grey boxes show the untranslated regions (UTRs). Each probe is represented by a vertical colored line, and its complementary site on an isoform is shown by a half circle (exon probe) or full circle (splice junction probe). The different colors indicate the combination of isoforms each probe targets. The NMD–affected isoform of each gene is indicated. The coding sequences (CDSs) of CG6946-RC, CG7283-RB, and CG16901-RD were identified as described in the text. (B) Normalized log_2_ fold-changes for the probes in the *upf1* experiment, grouped by which isoforms they target with colors corresponding to panel A. Each colored circle is the measurement of one probe on one array. The black circle is the group-wise mean of the fold-changes. (C) Deconvolved fold-change for the individual isoforms; “possibly absent” isoforms are not plotted (see text). (D) Estimated relative abundance of each present isoform in both the control and the NMD inhibited samples. We estimate that the NMD–target isoform of squid was a negligible fraction of total squid mRNA.

Deconvolution requires probes targeting different combinations of isoforms. For a gene with only two isoforms, we require probes targeting the two individual isoforms as well as probes targeting both isoforms; having only probes targeting the individual isoforms would preclude the estimation of relative abundance. As an example of a situation where deconvolution is impossible, alternative polyadenylation can produce two isoforms that differ only in the length of the last exon, and there is no possible probe that uniquely targets the shorter isoform. For genes with more than two isoforms the details are more subtle, but in general a gene with 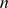 isoforms requires probes targeting at least 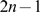 different combinations. This requirement makes it difficult to deconvolve genes with many isoforms, and, in some cases, it is provably impossible to obtain isoform-level fold-changes. Also, the algorithm and the array design assume that gene structures are known. Unknown alternative splice forms may lead to misinterpretation of the observed probe fold-changes. Examples of gene structures, probe locations, probe and isoform fold-changes, and relative proportions can be found in Figure 1.

The mathematical formulation of the generative model is presented in the Materials and Methods section. The algorithm should be of general use in integrating data from splicing-sensitive microarrays to infer isoform-level changes. The principles behind the algorithm can also be applied to other methods such as high-throughput mRNA sequencing for studying alternative splicing.

Using the algorithm, we were able to deconvolve the isoform-level fold-changes in the *upf1*-knockdown experiment for 1,410 of 1,576 genes with two isoforms and for 668 of 1,124 genes with three or more isoforms (involving as many as 11 isoforms). 574 of the genes were not deconvolved because they did not satisfy the requirement of having 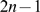 different probe combinations. The generative model imposes certain restrictions on the fold-change of a probe targeting multiple isoforms; 38 genes grossly violating these restrictions were flagged as inconsistent and no predictions were made for these genes.

Following deconvolution, we used statistical tests to classify the isoform-level changes. In microarray measurements, it is difficult to distinguish mRNAs whose levels do not change between conditions from mRNAs that are not present in either condition. For our study, we are most interested in cases where one isoform is differentially affected by NMD inhibition. An overall change in gene expression, with no change in splicing, can appear to be differential abundance of isoforms if one isoform is never present and is incorrectly called “unchanged.” To eliminate these false positives we devised a heuristic method to call isoforms “possibly absent,” at the expense of incorrectly eliminating some unchanged isoforms. The heuristic method is based on the reasoning that an mRNA should have positive evidence for its presence; in the absence of positive evidence we would rather conservatively conclude the transcript is absent than that it is present and unchanged. The “possibly absent” isoforms were excluded from later analyses, greatly improving the reliability of identified NMD–affected genes.

Using a 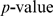 cutoff of 0.001, we found that 1,553 genes out of the 2,078 deconvolved genes show no change in expression upon inhibition of NMD. The remaining 525 genes have a total of 1,384 isoforms, of which 285 were “up-regulated,” 287 “slightly down-regulated,” 41 “very down-regulated,” 58 “unchanged,” and 713 were classified as “possibly absent.”

### Using isoform level fold-changes to determine response to NMD inhibition

In order to identify genes with isoforms targeted by NMD, we considered the joint behavior of all isoforms of the gene. To generate a high-confidence set of affected genes, we focused on high specificity with a consequent reduction in sensitivity. Therefore, our results do not provide an estimate of the prevalence of unproductive splicing, as many true NMD targets will be excluded by our criteria. To avoid making predictions based on secondary effects of the knockdown, we used the following reasoning:

1. An NMD–target mRNA should be degraded by NMD in the control sample, but not in NMD–inhibited cells. Thus, one isoform of the gene should be more abundant in the *upf1* knockdown sample relative to the control sample.
2. A nontarget mRNA should not be directly affected by NMD inhibition, although in some known examples the abundance of nontarget isoforms decreases slightly in NMD–inhibited cells, perhaps as a result of feedback. Thus, the other isoform of the same gene should not be differentially expressed or should be only slightly less abundant in the knockdown sample.

Using this classification scheme, a two-isoform gene is called an NMD target if the more abundant isoform is unchanged and the less abundant isoform is up-regulated upon NMD inhibition. As a result, this scheme primarily identifies NMD–affected genes that do not show gene-level differential expression, excluding most genes with a change in transcription level. For genes with more than two isoforms, we required that at least one isoform be up-regulated, at least one isoform be unchanged or only slightly down-regulated, and the rest of the isoforms be up-regulated, unchanged, slightly down-regulated, or possibly absent.

The full characterization of a gene as affected by NMD involves a number of sequential statistical tests. Correcting for multiple testing in a situation with nested tests is an open problem in statistics. We approach this problem by generating two sets of genes affected by NMD: one highest-confidence set where all significance levels were fixed at 0.001 (stringent) and one set where all levels were fixed at 0.05 (less stringent).

Our analysis of the *upf1* knockdown revealed 45 genes putatively affected by NMD with the stringent threshold (Table 1) and 189 genes putatively affected by NMD with the less stringent threshold (Tables S5, S6, S7, S8, S9, S10, S11, S12). We will focus on the stringent set throughout our analysis.

**Table 1:** UPF1 target genes.

gene ID	name	transcript	NMD status
CG1088	Vha26	CG1088-RB	target
		CG1088-RA	nontarget
CG1263	RpL8	CG1263-RB	target
		CG1263-RA	nontarget
CG12891	CPTI	CG12891-RB	target
		CG12891-RA	nontarget
CG13521	robo	CG13521-RA	target
		CG13521-RB	nontarget
CG13900		CG13900-RA	target
		CG13900-RB	nontarget
CG1753		CG1753-RB	target
		CG1753-RA	nontarget
CG18009	Trf2	CG18009-RA	target
		CG18009-RD	nontarget
CG1902		CG1902-RC	target
		CG1902-RA	nontarget
CG2152	Pcmt	CG2152-RB	target
		CG2152-RA	nontarget
CG33206	l(1)G0168	CG33206-RB	target
		CG33206-RA	nontarget
CG3358		CG3358-RA	target
		CG3358-RB	nontarget
CG3629	Dll	CG3629-RB	target
		CG3629-RA	nontarget
CG3731		CG3731-RA	target
		CG3731-RB	nontarget
CG4059	ftz-f1	CG4059-RA	target
		CG4059-RB	nontarget
CG4673		CG4673-RB	target
		CG4673-RA	nontarget
CG5215	Zn72D	CG5215-RA	target
		CG5215-RB	nontarget
CG5896	grass	CG5896-RA	target
		CG5896-RB	nontarget
CG6084		CG6084-RB	target
		CG6084-RA	nontarget
CG6315	fl(2)d	CG6315-RB	target
		CG6315-RA	nontarget
CG6454		CG6454-RA	target
		CG6454-RB	nontarget
CG7540	M6	CG7540-RA	target
		CG7540-RB	nontarget
CG8332	RpS15	CG8332-RB	target
		CG8332-RA	nontarget
CG9248		CG9248-RB	target
		CG9248-RA	nontarget
CG9354	RpL34b	CG9354-RA	target
		CG9354-RB	nontarget
CG9413		CG9413-RA	target
		CG9413-RB	nontarget
CG10107		CG10107-RA	target
		CG10107-RB	nontarget
		CG10107-RC	possibly absent
CG10948		CG10948-RB	target
		CG10948-RC	nontarget
		CG10948-RA	possibly absent
CG14217	Tao-1	CG14217-RB	target
		CG14217-RA	nontarget
		CG14217-RD	possibly absent
		CG14217-RE	possibly absent
CG14792	sta	CG14792-RB	target
		CG14792-RA	nontarget
		CG14792-RD	possibly absent
CG1623		CG1623-RC	target
		CG1623-RE	nontarget
		CG1623-RA	possibly absent
CG16901	sqd	CG16901-RD	target
		CG16901-RB	nontarget
		CG16901-RA	possibly absent
		CG16901-RC	possibly absent
CG17299	SNF4Agamma	CG17299-RG	target
		CG17299-RF	nontarget
		CG17299-RA	possibly absent
		CG17299-RB	possibly absent
		CG17299-RC	possibly absent
		CG17299-RD	possibly absent
		CG17299-RE	possibly absent
		CG17299-RH	possibly absent
CG17332	VhaSFD	CG17332-RA	target
		CG17332-RB	nontarget
		CG17332-RD	possibly absent
CG18069	CaMKII	CG18069-RB	target
		CG18069-RC	nontarget
		CG18069-RA	possibly absent
CG31237	Rpb4	CG31318-RA	target
		CG31237-RA	nontarget
		CG31318-RB	possibly absent
CG31305		CG31305-RA	target
		CG31305-RG	nontarget
		CG31305-RI	nontarget
		CG31305-RB	possibly absent
		CG31305-RD	possibly absent
		CG31305-RF	possibly absent
CG31332	unc-115	CG31332-RD	target
		CG31332-RB	nontarget
		CG31332-RC	nontarget
		CG31332-RA	possibly absent
CG31764	vir-1	CG31764-RA	target
		CG31764-RB	nontarget
		CG31764-RC	nontarget
CG32423	shep	CG32423-RD	target
		CG32423-RB	nontarget
		CG32423-RA	possibly absent
		CG32423-RC	possibly absent
CG33175	spri	CG33175-RG	target
		CG33175-RA	nontarget
		CG33175-RH	possibly absent
CG4376	Actn	CG4376-RB	target
		CG4376-RA	nontarget
		CG4376-RC	possibly absent
CG4452		CG4452-RB	target
		CG4452-RA	nontarget
		CG4452-RC	possibly absent
CG6854		CG6854-RA	target
		CG6854-RB	nontarget
		CG6854-RC	possibly absent
CG6946	glo	CG6946-RC	target
		CG6946-RA	nontarget
		CG6946-RB	possibly absent
CG7283	RpL10Ab	CG7283-RB	target
		CG7283-RA	nontarget
		CG7283-RC	possibly absent

Set of NMD targets for *upf1*.

### Genes affected by alternative splicing and NMD

We performed a Gene Ontology (GO) term enrichment analysis with the program AmiGO on the set of 45 affected genes to assess the effect of alternative splicing and NMD on cellular processes (Table 2, Tables S3, S4) [33]. The most significantly enriched biological process term, when comparing the NMD–target genes to all genes represented on our array, was “translation” (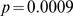, with no multiple testing correction), and parents of this term were also enriched. The NMD–target genes in this category encode five ribosomal proteins and two other RNA-binding proteins with roles in translation. The NMD–target genes also include another five genes encoding RNA-binding or splicing-related proteins, but related GO terms were not significantly enriched (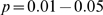). Terms related to the mitotic spindle were also enriched (*e.g.*, 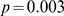 for “mitotic spindle elongation”). Interestingly, ribosomal protein genes were also largely responsible for this enrichment; many ribosomal proteins were previously identified in a genome-wide screen for mitotic spindle defects [34].

**Table 2:** GO analysis.

GO term	*p*-value	genes
protein metabolic process	0.0040	RpL8 RpL10Ab RpL34b RpS15 sta glo sqd CaMKII grass SNF4Agamma Tao-1 CG3731 CG10107
→ cellular protein metabolic process	0.0038	RpL8 RpL10Ab RpL34b RpS15 sta glo sqd CaMKII grass SNF4Agamma Tao-1 CG3731 CG10107
→→ translation	0.0009	RpL8 RpL10Ab RpL34b RpS15 sta glo sqd
microtubule cytoskeleton organization	0.0054	RpL8 RpL10Ab RpS15 sta CG13900 sqd
→ spindle organization	0.0086	RpL8 RpL10Ab RpS15 sta CG13900
→→ mitotic spindle organization	0.0041	RpL8 RpL10Ab RpS15 sta CG13900
→→ spindle elongation	0.0031	RpL8 RpL10Ab RpS15 sta
→→→ mitotic spindle elongation	0.0031	RpL8 RpL10Ab RpS15 sta

Significantly enriched GO biological process terms, showing terms as parent (top) to child (bottom). The ribosomal protein *sta* is also known as RpSA.

It was previously observed that knockdown of *upf1* or *upf2* caused cell cycle arrest in the G2/M phase [20]. To further investigate the connection between mitosis and NMD, we compared our set of NMD–affected genes to sets of genes associated with mitosis. Amongst our NMD targets, there was a significant enrichment (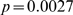) of a set of 402 genes with known mitotic defect phenotypes (119 of which were alternatively spliced and thus measured on our array) [34],[35]. The overlap comprised six genes, including the five genes with mitotic spindle GO annotations found in our AmiGO analysis. However, there was no enrichment of a set of 1000 genes that are co-expressed with known mitotic genes and likely to be differentially expressed in mitosis [35]. We believe it is unlikely that the mRNAs identified in our analysis as NMD targets are, instead, predominantly secondary effects of mitotic arrest, although we do not rule out the possibility that a subset of putative NMD targets actually represent such secondary effects. Indeed, the AmiGO results suggest that unproductive splicing of the six ribosomal and RNA-binding proteins may play a more direct role in cell cycle progression.

**Figure 2:**
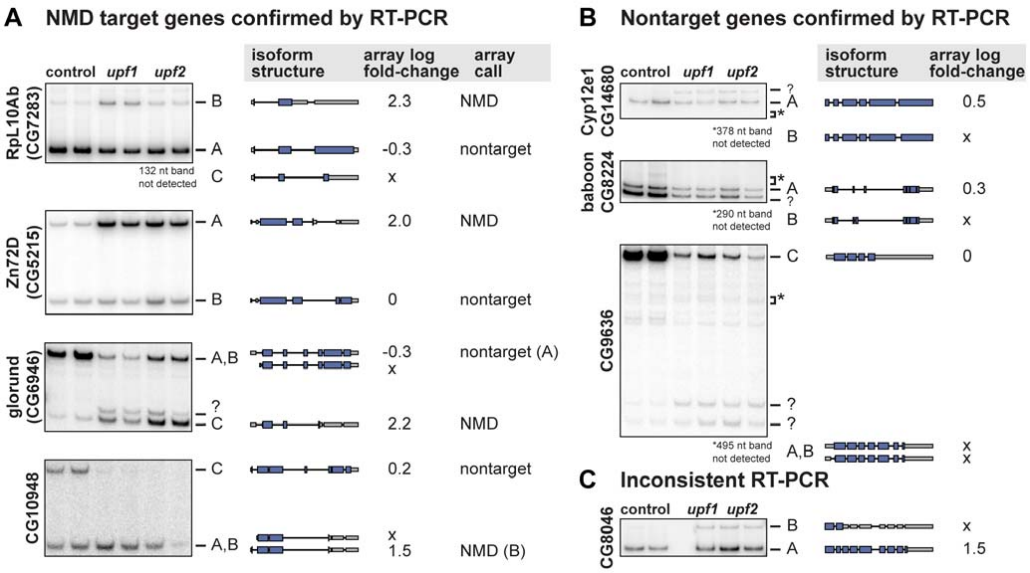
Experimental validation of NMD status. RNA samples isolated from control cells or cells depleted of UPF1 or UPF2 were analyzed by RT-PCR using primers flanking the alternative region of each gene. Bands corresponding to each isoform are labeled to the right and the exon/intron structure of each isoform is depicted along with the log_2_ fold-change estimated for that isoform from the array data when NMD is inhibited. “?” indicates a band of unknown origin and “*” shows the expected location of a band that is not observed. The isoform corresponding to the missing band is shown beneath the gel. “x” indicates an isoform called “possibly absent” on the array. Full gels are shown in Figure S10. (A) The action of NMD was confirmed on four genes called NMD–affected based on the array. For each gene, the ratio of NMD–target∶nontarget mRNA increased upon NMD inhibition. (B) Isoform classifications were confirmed for three genes called unaffected based on the array. (C) RT-PCR of CG8046 shows that NMD affects one isoform that was called “possibly absent” based on the array.

We experimentally tested the NMD status of isoforms of 10 genes chosen for having a large fold-change in at least one junction probe after *upf1* inhibition. Four of these genes had been called NMD–affected based on the microarray deconvolution, four genes had been called unaffected, and two genes had complex splicing patterns that had prevented their deconvolution. We used RT-PCR to measure the effect of *upf1* and *upf2* knockdowns on the 10 genes (Figure 2 and Figures S7, S8). We saw that the ratio of NMD–target∶nontarget mRNA increased upon *upf1* and *upf2* knockdown for all four genes called NMD–affected, confirming the array analysis. For three of the four genes called unaffected, we also confirmed the array analysis. One gene, CG8046, was called unaffected based on the array data, but RT-PCR showed that it is probably an NMD target because the ratio of isoform B∶A increases substantially upon NMD inhibition. Finally, two genes could not be deconvolved in the array analysis but have large individual probe fold-changes. Both genes, RpS9 and RpL3, are shown by RT-PCR to have an NMD–target isoform (Figure S7). In all, we found that the array analysis properly classified all isoforms of 7 out of 8 genes it was able to deconvolve. The analysis had no false positives, but as expected our analysis sometimes missed true NMD targets.

### Comparison with gene expression array results

Our results complement those of a previous study that identified NMD targets in *Drosophila* using a microarray approach that did not distinguish between alternative splice forms. Rehwinkel *et al.* used a gene expression microarray to measure the effect of inhibiting each of six NMD effectors [20]. They found that 525 mRNAs, or 14.3% of genes detected on the array, were up-regulated at least 1.5-fold after depleting UPF1. They focused on a core group of 184 genes that were up-regulated in at least 10 of their 12 knockdowns.

For each gene on our array, we compared the fold-change from the Rehwinkel *et al. upf1* knockdown with the gene-level fold-change from our analysis, obtained by averaging constitutive probes (Figures S5, S6). The two experiments have a correlation of 0.6.

As described above, our classification scheme focuses on genes that generally do not show differential expression at the gene level. For that reason, we would not expect a strong concordance between the NMD–affected genes identified in the two studies. Also, we only assayed genes annotated with multiple isoforms, which are only a small subset of the genes present on the Rehwinkel *et al.* platform. Indeed, there is almost no overlap between the two sets of inferred NMD targets; the only genes that were found to be affected by NMD in both studies are CG13900, CG10948, and *glorund*, all three involved in RNA processing.

Rehwinkel *et al.* validated the direct effect of NMD on nine genes, three of which were present on our array. One of these, CG13900, is an NMD target in our set. The other two genes are not classified as NMD targets in our results because they showed a change in overall expression rather than a differential effect on different isoforms. Rehwinkel's validation also demonstrated that two genes in their core set of NMD–affected genes, *pgi* and CG30035, do not appear to be direct NMD targets. Both genes were present on our array and both were correctly called nontargets.

### Reannotating coding regions reveals distinct features of NMD–target isoforms

Although the exact mechanism of premature stop codon recognition is unknown in *Drosophila*, it is generally assumed that NMD–target mRNAs have early stop codons relative to nontarget mRNAs. In light of this, it was startling that 35 of 45 genes in the set of NMD affected genes were annotated in FlyBase with the same stop codon in the NMD–target and nontarget isoforms. We determined that the annotated FlyBase coding sequence (CDS) was often unlikely to be the biologically accurate CDS. The FlyBase annotation protocol automatically chooses the longest open reading frame (ORF) of each transcript as the CDS, unless other evidence is available [36]. For the thousands of alternatively spliced genes, this annotation strategy may introduce substantial misinformation into gene and protein databases.

To understand the effect of NMD on a transcript, we identified the reading frame most likely to be recognized by the ribosome. In general, a eukaryotic ribosome initiates translation at the 5′-most AUG of an mRNA [37]. However, the ribosome may skip one or more AUG codons before initiating translation, or it may first translate a short upstream ORF (uORF) [38]. No single strategy for annotating reading frames will correctly represent the biology in all cases. We were guided by the principles that a gene should have at least one transcript that encodes a full-length, functional protein, and that the start codon of that transcript is likely to be recognized in the other, alternative transcripts as well.

**Figure 3:**
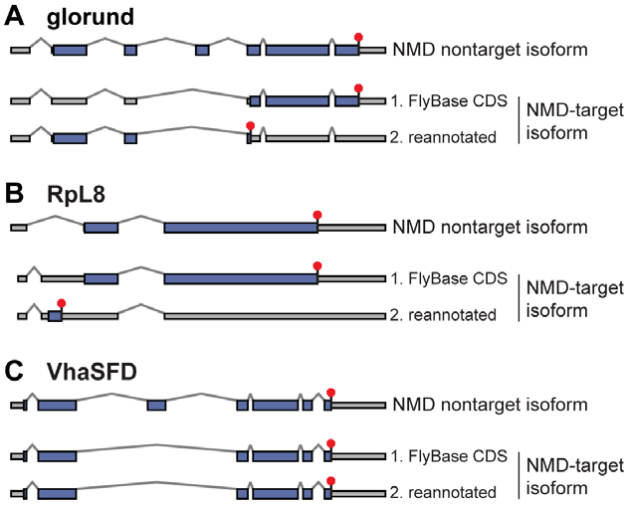
CDS reannotation. For each gene, the top diagram shows the gene structure including the CDS (blue rectangles), stop codon (red octagon), and UTRs (gray rectangles) of the isoform unaffected by NMD. The bottom diagrams show the gene structure of the NMD–affected isoform; (1) depicts the annotated CDS from FlyBase and (2) depicts the re-annotated CDS. (A) Re-annotation of glorund shows the existence of a likely CDS that shares its start codon with the unaffected isoform and has an early stop codon. (B) FlyBase annotates the same ORF in the unaffected and NMD–affected isoforms of RpL8. Re-annotation shows that alternative splicing introduces a uORF. (C) Our method does not find an alternate CDS in the NMD–affected isoform of VhaSFD, which differs from the unaffected isoform by skipping an in-frame cassette exon.

We employed two distinct methods to choose the correct CDS. One method makes use of the *upf1* knockdown data to help identify the transcript most likely to encode a full-length, functional protein. We assumed that in most cases this transcript would not be a target of NMD. Therefore, we chose the longest ORF found in any NMD nontarget isoforms of a given gene as the canonical CDS. We then assumed that the start of this canonical CDS is recognized *in vivo*, regardless of whether it begins at the first AUG codon in the transcript. We inferred the CDS of each isoform by choosing the ORF beginning at this canonical start codon (Figure 3). In some isoforms, alternative processing has introduced isoform-specific sequence upstream of the canonical start codon, *e.g.*, due to an upstream promoter or alternative splicing in the first intron (Figure 3B). In these cases, we considered the possibility that the alternative sequence contains a new, upstream AUG that is recognized by the ribosome, perhaps as the start of a short uORF with an early stop codon.

The second method to annotate CDSs is blind to NMD status. The longest ORF present in any transcript, NMD–target or nontarget, was chosen as the canonical CDS, and its start codon was used to annotate the CDS in all transcripts. This second method has the benefit of being unbiased, but because it ignores some data, it is likely to be less accurate. The results from this second, unbiased method were used in our statistical analysis of gene features correlated with NMD status. Full details of our reannotation methods are found in the Supplementary Methods in Text S1.

Basing the CDS annotation on the NMD status of each transcript in the set of 45 *upf1*-affected genes, the first annotation algorithm found 27 genes with a noticeably early stop codon in the NMD–target isoform relative to the nontarget isoform, out of 41 genes (four genes were removed from the analysis because of inconsistencies between FlyBase 4 and more recent transcript data). Without using NMD status as input, the second annotation algorithm found early stop codons in 23 out of 41 genes. The NMD–affected isoforms without early stop codons may represent unknown aspects of the NMD mechanism or, more likely, secondary effects of the knockdowns. We also re-annotated the CDS of the *upf1*-affected genes identified with the less stringent 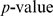 cutoff. Early stop codons are found in a lower percent of the NMD targets in this set compared to the strict set: 92/181 using NMD status, and 65/181 without using NMD status. This suggests that the less stringent 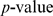 may include more genes that are not directly affected by NMD.

If the NMD–target mRNAs do not encode functional proteins, we would not expect their CDSs to be optimized for translation efficiency or under selective pressure to maintain amino acid sequence. A comparison to overall *Drosophila* codon usage showed that the nontarget mRNAs were significantly skewed towards preferred codons and the NMD–target mRNAs showed less preference for preferred codons. This indicates that the unproductive reading frames are less optimized for translation efficiency. We also estimated the ratio of non-synonymous to synonymous substitutions (dN/dS) in dual-coding regions in which the reading frame of the NMD–target isoform is shifted relative to the nontarget isoform, comparing *D. melanogaster* to *D. ananassae* using PAL2NAL [39] (Supplementary Results in Text S1). In 3 of 4 dual-coding regions, from *glo*, *robo*, and CG4452, the NMD–target reading frame had a very high dN/dS, indicating that it was probably not under coding sequence constraints, and the nontarget reading frame had a low dN/dS as expected. In only one gene, CG9413, dN/dS was lower in the NMD–target reading frame than in the nontarget reading frame, indicating that this sequence might be under protein-coding constraints in both frames. Overall, these results suggest that our CDS annotation was generally accurate, and support the notion that our NMD–target mRNAs do not yield protein.

### Identification of mRNA features correlated with NMD status

We sought to find features of mRNAs that were correlated with NMD target status. These features could reveal aspects of the NMD mechanism for recognizing premature stop codons. We considered the lengths of the 5′ UTR, 3′ UTR, and CDS; the number of introns in the UTRs, the CDS, and the transcript as a whole; the number and size of potential uORFs; and sequence features such as A-rich regions. These features were chosen based on existing hypotheses about NMD. The presence of introns in the 3′ UTR triggers NMD in human, while the length of the 3′ UTR has been implicated in NMD in *Drosophila* [25]. Small upstream ORFs might trigger NMD of some transcripts [40], and A-rich elements in mammalian 5′ UTRs also destabilize some mRNAs via the binding of PABPC1 [41]. Although experiments have shown that NMD of a reporter construct in *Drosophila* does not depend on components of the exon junction complex [22], we also tested the possibility of a rule akin to the human 50-nucleotide rule. We computed the distance between the stop codon and the position of the last exon junction in the transcript.

**Figure 4:**
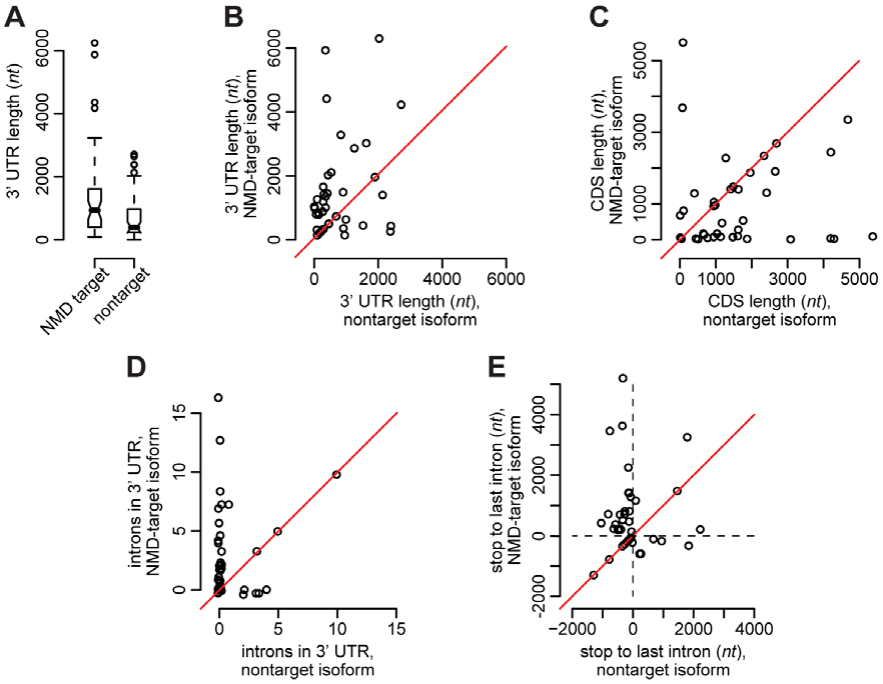
Features correlated with NMD status. (A) Boxplots of the 3′ UTR length comparing the strict set of *upf1* NMD–target mRNAs to the set of *upf1* NMD nontarget mRNAs from the same genes. The lower box indicates the second quartile of values and the upper box the third quartile, the belt shows the median, and the whiskers indicate the largest value within 1.5× the size of the box. (B) The 3′ UTR length, (C) CDS length, (D) number of introns in the 3′ UTR, and (E) distance between the stop codon and the last intron, compared per gene for each gene in the strict set of *upf1*-affected genes. These pairwise comparisons show more significant differences than the comparison in panel (A).

The NMD targets and NMD nontargets were first compared using an unpaired analysis, where we compare the marginal feature distributions for each of the two sets of isoforms (Figure 4A). Such a comparison yielded little difference between the two groups, mostly due to high heterogeneity between genes relative to differences between distinct isoforms of the same gene. We therefore proceeded with a more powerful paired analysis in which we compared each feature of the NMD–target isoform with the corresponding feature of the NMD nontarget isoform for the same gene. In case a gene has two or more isoforms that are labeled target or nontarget, the feature values for the isoforms of the given gene were averaged to yield a single number per gene per category. For each comparison we considered both one- and two-sided tests with the alternative hypothesis that the NMD–target isoforms have, for instance, longer 3′ UTRs or more introns in the 3′ UTR region.

We found six features to be correlated with NMD status in the *upf1*-affected genes (Figure 4 and Figures S9, S10, S11, S12, S13, S14, S15, S16, S17, S18, S19, S20, S21). Relative to the nontarget isoforms, the NMD–target isoforms have shorter CDSs, fewer introns in the CDS, longer 3′ UTRs, more introns in the 3′ UTR, longer ORFs in the 3′ UTR, and a greater distance between the stop codon and the last intron. All of these features were significant at a 5% level with 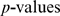 between 0.0008 and 0.003 (one-sided tests; 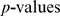 for two-sided tests were twice as large) for the stringent set of *upf1*-affected genes. All six features had somewhat less significant 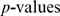 using the less stringent set of targets (between 0.042 and 0.09 for one-sided tests).

In our set of NMD–affected genes we find that there are essentially two subgroups. One subgroup of genes shows the differences described above, with longer 3′ UTRs in NMD–target mRNAs. In the other group, the NMD target and nontarget isoforms of a given gene share the same 3′ UTR structure – implying that no feature in the 3′ part of the gene can be responsible for NMD recognition. Some of these genes might have been classified incorrectly and may instead reflect secondary effects of NMD inhibition.

We also used MEME to search for overrepresented sequence motifs within the 3′ UTRs of NMD–target mRNAs [42], analogous to the downstream element implicated in NMD in yeast [43]. The only motifs found to be enriched within the UTRs of NMD–target mRNAs were repetitive sequences (Figure S22A). When we limited the search to the UTRs of NMD–target mRNAs with early stop codons, we found two additional non-repetitive motifs (Figure S22A), but both are similar to known splicing enhancers [44]. No significant motifs were found in the UTRs of the nontarget mRNAs.

### Insight into the *Drosophila* NMD mechanism

The features correlated with NMD status are obviously not independent, and our data cannot resolve which, if any, of these are detected directly by the NMD mechanism. Alternative splicing has only a small effect on the length of the mRNAs produced from most of the NMD–affected genes; its principal effect is to change the position of the stop codon, simultaneously shortening the CDS and lengthening the 3′ UTR. The change in 3′ UTR length may also account for the significance of the other features that distinguish NMD–target from nontarget isoforms.

Our observation of longer 3′ UTRs agrees with previous work indicating that the NMD mechanism in *Drosophila* is affected by the length of the 3′ UTR. Behm-Ansmant *et al.* determined that nonsense codons in an *adh* reporter construct are recognized as premature based on the distance between the stop codon and PABPC1 bound to the poly-A tail of the transcript [25]. Stop codons 379 nucleotides or fewer upstream of the poly-A tail did not elicit NMD, but stop codons 397 nt or more upstream of the poly-A tail caused degradation. Our larger set of natural NMD targets allows us to compare this length to the UTR lengths of the transcripts identified by our array to see if that result is more generally applicable.

We found that on average the NMD–target isoforms have longer 3′ UTRs than the nontarget isoforms, but 397 nt is not a discriminant. Almost all (33/41) of the NMD–target isoforms have UTRs longer than 397 nt, but over a third (17/44) of the nontarget isoforms also have UTRs longer than 397 nt. It may be more appropriate to include only genes that are more likely to be direct NMD targets. The 397 nt cutoff does describe all but one of the 27 NMD–target isoforms with an early stop codon relative to the non-target isoform of the same gene. However, 9 of 28 nontarget isoforms also have a 3′ UTR longer than 397 nt. From our data, the best descriptor seems to be a length cutoff of 742 nt, which correctly classifies 26/27 NMD–target mRNAs and 22/28 nontarget mRNAs. It is clear that the length of the 3′ UTR is a key determinant of NMD, but neither our statistical correlation nor the published experimental study provide a general rule for predicting NMD status.

## Discussion

We have found that alternative splicing in *Drosophila* can produce mRNAs that are targets of NMD. Using strict criteria, we find 45 genes with both an isoform that is stabilized after NMD inhibition and an isoform that is not affected by NMD inhibition. Our set includes examples of many different modes of alternative processing, including cassette exon skipping or inclusion, alternative 5′ or 3′ splice sites, intron retention, and alternative splicing combined with alternative transcription start sites or polyadenylation. Note that our conservative criteria are not intended to provide a full measure of the true prevalence of unproductive splicing in fly.

Most of the NMD–target isoforms have early stop codons relative to the unaffected isoform of the same gene, indicating that our results include many direct targets of NMD. However, a third of the apparent NMD–target isoforms in the stringent set do not have early stop codons. While some may be false positives, others are likely to represent secondary effects of NMD inhibition, genes with unannotated alternative splicing events, or unknown aspects of the NMD pathway. The NMD machinery may recognize and degrade some mRNAs whose stop codons do not appear premature, as occurs in the mammalian UPF1-dependent process known as Staufen mediated decay [45],[46].

Many NMD–affected genes without early stop codons may not be direct targets of NMD and may instead demonstrate the downstream effects of unproductive splicing. Secondary splicing effects are particularly likely in cases when splicing factors are direct targets of NMD. In *C. elegans*, the altered expression of splicing factors after NMD inhibition may affect the splicing of numerous genes [30]. Our set of NMD–affected genes includes at least seven genes encoding characterized RNA-binding or splicing-related proteins. One of these splicing factors, Squid, is known to affect the splicing of at least 255 other genes [29]. Among its targets are five genes identified as NMD–affected in this study, and one of these genes has no early stop codon. There may be additional splicing factors affected by NMD which our methods overlooked. Alternative isoforms of the SR splicing factor B52 were dramatically stabilized upon NMD inhibition, but this gene was not classified as an NMD target because we could not determine if the nontarget isoforms were present. Ten NMD–affected genes in our results are known targets of B52, three of which have no early stop codon [19],[27]. Our set of targets also includes at least two transcription factors, Dll and FTZ-F1, and altered transcription may thus be a secondary effect of inhibiting NMD. Further, *upf1* and *upf2* knockdowns cause cell cycle arrest in mitosis [20], which may cause secondary splicing effects and confound our interpretation of NMD targets. However, the six known mitosis-related genes amongst our targets almost all have early stop codons and thus appear likely to be direct NMD targets. This leads to the intriguing possibility that the mitotic arrest phenotype is due to the misregulation of specific unproductive splicing events after NMD inhibition.

NMD was initially identified for its role in clearing the cell of erroneous and potentially harmful mRNAs. However, unproductive splicing can also be used to regulate gene expression. In mammalian systems, alternative splicing and NMD are combined to regulate the expression of numerous genes. RNA-binding proteins and ribosomal proteins, in particular, seem to employ unproductive splicing to autoregulate their expression, perhaps to maintain homeostasis ([11],[12],[47],[48]; reviewed in [49]). We have shown that this theme is continued in *Drosophila*. Many translation and splicing-related proteins are found in our set of fly NMD targets, and further investigation may elucidate important roles of unproductive splicing in the regulation of *Drosophila* processes.

## Materials and Methods

### RNA interference and microarray hybridization

RNA interference was performed against *upf1* and *upf2* and RNA was obtained from cultured *Drosophila* Schneider cells as described in [20]. As a reference, RNA was obtained from mock-treated cells as in [20]. Samples from three independent knockdowns of *upf1* and *upf2* were amplified, labeled, and hybridized onto a custom two-color microarray as described in [27].

### RT–PCR validation of NMD targets

Reverse transcription and amplification were performed as described in [27]. For each experiment, 1 µg of RNA was reverse transcribed using SuperScript II (Invitrogen) following the manufacturer's protocol. One-fiftieth of the RT reaction was used in a PCR reaction with Taq polymerase (NEB) following the manufacturer's protocol. PCR primers were chosen to flank the alternatively spliced region and the primer sequences are available upon request.

### Microarray design

The microarray was manufactured by Agilent using the 44 k platform with a custom array design. The array was designed using the methods described in [27], but updated to use data from FlyBase 4.0. The updated array design had two improvements: the exonic probes were chosen to be isothermal with the average 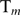 of the junction probes, and a 20-nt dT stilt was added to decrease the chance of steric hindrance between the labeled probes and the glass surface. The 43,337 probes on the array (excluding control probes) measure the following target sequences: 10,694 alternative exons or splice junctions, 25,213 constitutive exons or splice junctions, 2,798 alternative polyadenylation events, and 4,632 alternative transcription start events. In addition, there are 10 negative controls and 23 positive controls. In total, the array interrogates 7,768 transcripts of 2,793 genes.

### Microarray preprocessing

The image analysis was performed by Agilent Feature Extraction version 7.5.1. The scanned images were preprocessed using the limma package [50] from Bioconductor release 2.1 [51]. The background correction was done using the normexp method [52], with an offset of 10, and was followed by loess normalization between the red and the green channel within each array. Raw and preprocessed data have been submitted to GEO with accession number GSE13532.

### Isoform deconvolution

As a motivating example we start by considering the behavior of a probe targeting two different isoforms of the same gene (for example, an exon probe for a constitutively expressed exon). Let 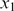 and 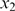 be the absolute amounts of mRNA of isoforms 1 and 2 in the control sample and let 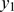 and 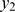 be the absolute amounts of mRNA of isoforms 1 and 2 in the treatment sample. The treatment-control fold-change for the probe is then
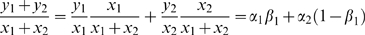
(1)with 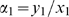, 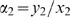, 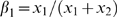. We recognize 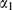 and 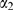 as the fold-changes associated with isoforms 1 and 2 and 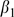 as the relative proportion of isoform 1 in the control sample. These relative expression parameters are estimable from a microarray experiment, as opposed to the absolute mRNA amounts.

This approach can be immediately generalized to a probe targeting 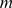 out of 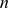 isoforms of a given gene. In this case, the treatment-control fold-change associated with such a probe becomes
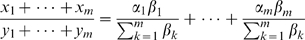
(2)with 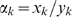 being the fold-change associated with the 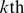 isoform and 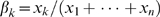 being the relative proportion of isoform 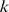 out of all 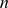 isoforms.

Because noise in microarray experiments appears to be additive on the log scale, we propose the following model
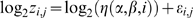
(3)with 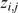 being the observed fold-change for probe 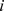 and sample 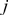,
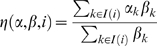
(4)being the fold-change parameter defined above, and 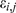 being a noise term. 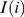 is a function that for every probe 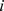 yields which isoforms the probe targets.

We propose to estimate the parameters 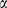 and 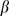 using non-linear least squares, *i.e.*, by solving the following minimization problem
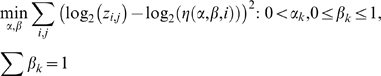
(5)Based on a heuristic argument, we expect the presence of the logarithm to turn this into a non-convex optimization problem.

A variant of this minimization problem, where the constraint 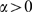 is replaced by 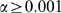, is solved using an adaptive barrier method proposed by [53] and implemented in the R function constrOptim, using a collection of suitably chosen starting points intermixed with random points.

Hypotheses related to the differential expression parameters, such as 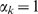 (is isoform 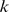 not differentially expressed) or 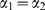 (are isoforms 1 and 2 similarly expressed), are tested using F-statistics (for details see a reference on non-linear regression such as [54]).

### NMD calls

**Figure 5:**
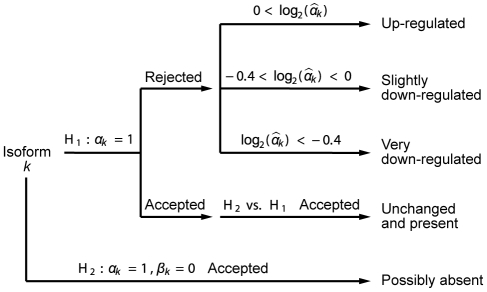
Isoform classification. Flowchart for isoform classification. Each isoform is classified separately. 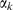 is the non-log fold-change associated with isoform 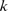 and 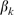 is the relative proportion of the isoform in the control sample.

For each gene, isoform-level measures were deconvolved using the approach described above, and each isoform classified according to the process depicted in Figure 5. For every isoform in the gene, the following hypotheses were tested: 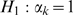 (is isoform 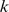 not differentially expressed), 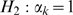, 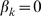 (is isoform 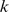 possibly absent). Any given hypothesis was considered rejected if the nominal 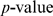 was lower than 0.001 (“stringent” set) or 0.05 (“less stringent” set) and accepted otherwise. An isoform was characterized as “up-regulated” if 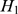 was rejected and 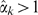, “slightly down-regulated” if 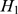 was rejected and 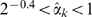, “very down-regulated” if 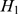 was rejected and 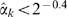, “unchanged and present” if 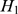 was accepted and a nested test of 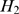 against 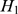 was rejected, and finally as “possibly absent” if 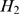 was accepted (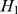 was not tested for this classification). With this characterization, it is possible for an isoform to be labeled as “unchanged and present” as well as “possibly absent.” In that case, “possibly absent” takes precedence.

Based on this characterization, a gene was labeled as “NMD affected” if at least one isoform was “up-regulated,” at least one isoform was “slightly down-regulated” or “unchanged and present,” and the remaining isoforms were either “slightly down-regulated,” “unchanged and present” or “possibly absent.” The isoforms for such a gene were labeled as either “NMD target,” “NMD nontarget,” or “possibly absent.” These labels were used as input in the feature correlation.

### GO analysis

We used AmiGO [33] to compare the GO terms of all genes on our array vs both the strict and less-strict sets of NMD–affected genes, with a p-value cutoff of 0.01. Annotations were obtained from FlyBase via AmiGO.

### Feature correlation

For every gene that had an isoform affected by NMD, we labeled the isoforms affected by NMD as NMD targets, the isoforms present but not affected by NMD as NMD nontargets, and we discarded the isoforms that were not present.

For each feature we performed a paired as well as an unpaired analysis. The unpaired analysis compares the distribution of a feature for the NMD–target isoforms to the corresponding distribution for the NMD nontarget isoforms. The paired analysis computes, for each gene, the difference between the feature for the NMD–target isoform and the feature for the NMD nontarget isoform. The distribution of these differences are then compared to zero. In case there were two or more isoforms in a group, the values of the feature were averaged. As expected, we found the paired comparison to be more powerful.

Specifically, for every feature, we performed a Wilcoxon rank sum test with exact permutation 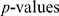. The test was either paired or unpaired depending on the analysis being done. The permutation 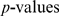 were computed using the package *coin*, see [55]. We also visually inspected the distributions using boxplots and scatterplots; see Figures S9, S10, S11, S12, S13, S14, S15, S16, S17, S18, S19, S20, S21 and Tables S1, S2.

## Supporting Information

Figure S1 Overlayed MA plots for *upf1*. MA plot of the 3 normalized *upf1* arrays. The three arrays have been superimposed on the same plot. The red line is a lowess line, and the blue line is *M* = 0. The yellow/green points are probes targeting *upf1/upf2* and the blue/purple points are positive (present)/negative (absent) control probes.
(0.79 MB PDF)

Figure S2 Overlayed MA plots for *upf2*. As Figure S1, but for *upf2*.
(0.79 MB PDF)

Figure S3 Individual MA plots for *upf1*. MA plots of the 3 normalized *upf1* arrays side by side. Lines and colorscheme are as in Figure S1.
(2.28 MB PDF)

Figure S4 Individual MA plots for *upf2*. MA plots of the 3 normalized *upf2* arrays side by side. Lines and colorscheme are as in Figure S1.
(2.26 MB PDF)

Figure S5 Smoothed scatterplot of *upf1* and Affymetrix data. Smoothed scatterplot of gene level log_2_ fold changes from this study versus Rehwinkel *et al.*, from the *upf1* arrays. Rehwinkel's normalized data was obtained from Array Express E-MEXP-202. Genes that were labelled as absent in either study were removed. The blue line is *y* = *x* and the red line is lowess smoother. Our gene level fold changes were obtained by averaging all probes annotated as being constitutive. Using only constitutive exon probes instead of constitutive exon and junction probes did not qualitatively change the scatterplot.
(0.68 MB PDF)

Figure S6 Smoothed scatterplot of *upf2* and Affymetrix data. As Figure S5, but for *upf2*.
(0.68 MB PDF)

Figure S7 RT-PCR shows NMD in RpS9 and RpL3. RT-PCR shows NMD-target isoforms of RpS9 and RpL3. Neither gene could be deconvolved by our array analysis.
(0.51 MB PDF)

Figure S8 RT-PCR validation of NMD. Full gels from the RT-PCR validation described in main Figure 1.
(1.45 MB PDF)

Figure S9 Length of 5′ UTR. Top right and top left plots are boxplots comparing the set of NMD-target mRNAs to the set of NMD nontarget mRNAs from the same genes, for both the stringent and the less stringent set of *upf1*-affected genes. Bottom right and bottom left are scatterplots between mRNAs of the same gene, labelled as either NMD-target or NMD nontarget, for both the stringent and the less stringent set of genes. The feature in question is the “length of 5′ UTR.” The scatterplots have an aspect ratio of 1.
(0.06 MB PDF)

Figure S10 Length of longest ORF in the 5′ UTR. As Figure S9 for the feature “length of longest ORF in 5′ UTR.” The bottom left scatterplot has been jittered.
(0.05 MB PDF)

Figure S11 Fraction of A nucleotides in 5′ UTR. As Figure S9 for the feature “fraction of A nucleotides in 5′ UTR.” The bottom left scatterplot has been jittered.
(0.05 MB PDF)

Figure S12 Length of longest A-rich region in 5′ UTR. As Figure S9 for the feature “length of longest A-rich region in 5′ UTR.” The bottom left scatterplot has been jittered.
(0.05 MB PDF)

Figure S13 Length of 3′ UTR. As Figure S9 for the feature “length of 3′ UTR.”
(0.05 MB PDF)

Figure S14 Number of introns in 3′ UTR. As Figure S9 for the feature “number of introns in 3′ UTR.” Both the bottom right and the bottom left scatterplots have been jittered.
(0.05 MB PDF)

Figure S15 Length of longest ORF in 3′ UTR. As Figure S9 for the feature “length of longest ORF in 2′ UTR.”
(0.05 MB PDF)

Figure S16 Fraction of A nucleotides in 3′ UTR. As Figure S9 for the feature “fraction of A nucleotides in 3′ UTR.”
(0.05 MB PDF)

Figure S17 Length of longest A-rich region in 3′ UTR. As Figure S9 for the feature “length of longest A-rich region in 3′ UTR.”
(0.05 MB PDF)

Figure S18 Number of introns in transcript. As Figure S9 for the feature “number of introns in transcript.” The bottom left scatterplot has been jittered.
(0.05 MB PDF)

Figure S19 Length of CDS. As Figure S9 for the feature “length of CDS.”
(0.05 MB PDF)

Figure S20 Number of introns in CDS. As Figure S9 for the feature “number of introns in CDS.” The bottom left scatterplot has been jittered.
(0.05 MB PDF)

Figure S21 Distance from stop codon to final intron. As Figure S9 for the feature “distance from stop codon to final intron.” CG11100 did not have any introns and is not assigned a value.
(0.05 MB PDF)

Figure S22 Motifs found by MEME. A. Overrepresented motifs found in the 3′ UTRs of NMD-target mRNAs. MEME was used to search for motifs with a width of 6–16 nt occurring zero or one times per sequence. All motifs appear to be repetitive sequence. B. Overrepresented motifs found in the 3′ UTRs of the 27 NMD-target mRNAs with an early stop codon. Motifs 2, 3, and 5 match the motifs in (A); motifs 1 and 4 appear similar to known RARRAR splicing enhancers.
(0.47 MB PDF)

Table S1 Number of AUG (start) codons in 5′ UTR. Tables (a) and (b) show an unpaired comparison between the NMD-target mRNAs and the NMD nontarget mRNAs for the stringent and the less stringent set of *upf1*-affected genes, for the feature “number of AUG (start) codons in 5′ UTR.” Tables (c) and (d) show a paired comparison between the two sets of mRNAs, for the stringent and the less stringent set of genes.
(0.04 MB PDF)

Table S2 Number of introns in 5′ UTR. As Table S1, but for the feature “number of introns in 5′ UTR.”
(0.03 MB PDF)

Table S3 GO terms enriched in stringent *upf1* set.
(0.04 MB PDF)

Table S4 GO terms enriched in less stringent *upf1* set.
(0.03 MB PDF)

Table S5 Number of genes successfully deconvolved. The number of genes deconvolved by the analysis. “Deconvolved” indicates that the deconvolution was successful, “too few configurations” indicates the there were too few probe configurations to deconvolve the gene and “inconsistent” indicates that the gene could be deconvolved, but the estimates were inconsistent with the gene model.
(0.03 MB PDF)

Table S6 *upf2* target genes, stringent set. Set of NMD targets for *upf2*, using the stringent cutoff.
(0.04 MB PDF)

Table S7 *upf1* target genes, less stringent set.
(0.05 MB PDF)

Table S8 *upf2* target genes, less stringent set.
(0.04 MB PDF)

Table S9 Deconvolution results for the stringent set of *upf1* affected genes.
(0.04 MB PDF)

Table S10 Deconvolution results for the less stringent set of *upf1* affected genes.
(0.05 MB PDF)

Table S11 Deconvolution results for the stringent set of *upf2* affected genes.
(0.03 MB PDF)

Table S12 Deconvolution results for the less stringent set of *upf2* affected genes.
(0.04 MB PDF)

Text S1 Supplementary results and methods.
(0.08 MB PDF)

## References

[1] Culbertson MR, Leeds PF (2003). Looking at mRNA decay pathways through the window of molecular evolution.. Curr Opin Genet Dev.

[2] Cali BM, Anderson P (1998). mRNA surveillance mitigates genetic dominance in *Caenorhabditis elegans*.. Mol Gen Genet.

[3] Mendell JT, Sharifi NA, Meyers JL, Martinez-Murillo F, Dietz HC (2004). Nonsense surveillance regulates expression of diverse classes of mammalian transcripts and mutes genomic noise.. Nat Genet.

[4] Pan Q, Saltzman AL, Kim YK, Misquitta C, Shai O (2006). Quantitative microarray profiling provides evidence against widespread coupling of alternative splicing with nonsense-mediated mRNA decay to control gene expression.. Genes Dev.

[5] Isken O, Maquat LE (2007). Quality control of eukaryotic mRNA: safeguarding cells from abnormal mRNA function.. Genes Dev.

[6] Rehwinkel J, Raes J, Izaurralde E (2006). Nonsense-mediated mRNA decay: Target genes and functional diversification of effectors.. Trends Biochem Sci.

[7] Lewis BP, Green RE, Brenner SE (2003). Evidence for the widespread coupling of alternative splicing and nonsense-mediated mRNA decay in humans.. Proc Natl Acad Sci U S A.

[8] Lareau LF, Brooks AN, Soergel DAW, Meng Q, Brenner SE, Blencowe BJ, Graveley BR (2007). The coupling of alternative splicing and nonsense mediated mRNA decay.. Alternative splicing in the postgenomic era, Landes Biosciences.

[9] Sureau A, Gattoni R, Dooghe Y, Stevenin J, Soret J (2001). SC35 autoregulates its expression by promoting splicing events that destabilize its mRNAs.. EMBO J.

[10] Wollerton MC, Gooding C, Wagner EJ, Garcia-Blanco MA, Smith CWJ (2004). Autoregulation of polypyrimidine tract binding protein by alternative splicing leading to nonsense-mediated decay.. Mol Cell.

[11] Lareau LF, Inada M, Green RE, Wengrod JC, Brenner SE (2007). Unproductive splicing of SR genes associated with highly conserved and ultraconserved DNA elements.. Nature.

[12] Ni JZ, Grate L, Donohue JP, Preston C, Nobida N (2007). Ultraconserved elements are associated with homeostatic control of splicing regulators by alternative splicing and nonsense-mediated decay.. Genes Dev.

[13] Saltzman AL, Kim YK, Pan Q, Fagnani MM, Maquat LE (2008). Regulation of multiple core spliceosomal proteins by alternative splicing-coupled nonsense-mediated mRNA decay.. Mol Cell Biol.

[14] Rossbach O, Hung LH, Schreiner S, Grishina I, Heiner M (2009). Auto- and cross-regulation of the hnRNP L proteins by alternative splicing.. Mol Cell Biol.

[15] Hyvonen MT, Uimari A, Keinanen TA, Heikkinen S, Pellinen R (2006). Polyamine-regulated unproductive splicing and translation of spermidine/spermine N1-acetyltransferase.. RNA.

[16] Stolc V, Gauhar Z, Mason C, Halasz G, van Batenburg MF (2004). A gene expression map for the euchromatic genome of *Drosophila melanogaster*.. Science.

[17] Bell LR, Maine EM, Schedl P, Cline TW (1988). Sex-lethal, a *Drosophila* sex determination switch gene, exhibits sex-specific RNA splicing and sequence similarity to RNA binding proteins.. Cell.

[18] Hattori D, Demir E, Kim HW, Viragh E, Zipursky SL (2007). Dscam diversity is essential for neuronal wiring and self-recognition.. Nature.

[19] Gabut M, Dejardin J, Tazi J, Soret J (2007). The SR family proteins B52 and dASF/SF2 modulate development of the *Drosophila* visual system by regulating specific RNA targets.. Mol Cell Biol.

[20] Rehwinkel J, Letunic I, Raes J, Bork P, Izaurralde E (2005). Nonsense-mediated mRNA decay factors act in concert to regulate common mRNA targets.. RNA.

[21] Alonso CR, Akam M (2003). A Hox gene mutation that triggers nonsense-mediated RNA decay and affects alternative splicing during *Drosophila* development.. Nucleic Acids Res.

[22] Gatfield D, Unterholzner L, Ciccarelli FD, Bork P, Izaurralde E (2003). Nonsense-mediated mRNA decay in *Drosophila*: at the intersection of the yeast and mammalian pathways.. EMBO J.

[23] Le Hir H, Izaurralde E, Maquat LE, Moore MJ (2000). The spliceosome deposits multiple proteins 20–24 nucleotides upstream of mRNA exon-exon junctions.. EMBO J.

[24] Nagy E, Maquat LE (1998). A rule for termination-codon position within intron-containing genes: when nonsense affects RNA abundance.. Trends Biochem Sci.

[25] Behm-Ansmant I, Gatfield D, Rehwinkel J, Hilgers V, Izaurralde E (2007). A conserved role for cytoplasmic poly(A)-binding protein 1 (PABPC1) in nonsense-mediated mRNA decay.. EMBO J.

[26] Calarco JA, Saltzman AL, Ip JY, Blencowe BJ, Blencowe BJ, Graveley BR (2007). Technologies for the global discovery and analysis of alternative splicing.. Alternative splicing in the postgenomic era, Landes Biosciences.

[27] Blanchette M, Green RE, Brenner SE, Rio DC (2005). Global analysis of positive and negative pre-mRNA splicing regulators in *Drosophila*.. Genes Dev.

[28] McIntyre LM, Bono LM, Genissel A, Westerman R, Junk D (2006). Sex-specific expression of alternative transcripts in *Drosophila*.. Genome Biol.

[29] Blanchette M, Green RE, MacArthur S, Brooks AN, Brenner SE (2009). Genome-wide analysis of alternative pre-mRNA splicing and RNA-binding specificities of the Drosophila hnRNP A/B family members.. Mol Cell.

[30] Barberan-Soler S, Zahler AM (2008). Alternative splicing regulation during *C. elegans* development: splicing factors as regulated targets.. PLoS Genet.

[31] Sayani S, Janis M, Lee CY, Toesca I, Chanfreau GF (2008). Widespread impact of nonsense-mediated mrna decay on the yeast intronome.. Mol Cell.

[32] Shai O, Morris QD, Blencowe BJ, Frey BJ (2006). Inferring global levels of alternative splicing isoforms using a generative model of microarray data.. Bioinformatics.

[33] Carbon S, Ireland A, Mungall CJ, Shu S, Marshall B (2009). AmiGO: online access to ontology and annotation data.. Bioinformatics.

[34] Goshima G, Wollman R, Goodwin SS, Zhang N, Scholey JM (2007). Genes required for mitotic spindle assembly in Drosophila S2 cells.. Science.

[35] Somma MP, Ceprani F, Bucciarelli E, Naim V, De Arcangelis V (2008). Identification of Drosophila mitotic genes by combining co-expression analysis and RNA interference.. PLoS Genet.

[36] FlyBase (2007) Polypeptide report help. Available: http://flybase.org/static_pages/newhelp/polypeptide_help.html. Accessed 28 May 2009

[37] Kozak M (1989). The scanning model for translation: an update.. J Cell Biol.

[38] Kozak M (2002). Pushing the limits of the scanning mechanism for initiation of translation.. Gene.

[39] Suyama M, Torrents D, Bork P (2006). PAL2NAL: robust conversion of protein sequence alignments into the corresponding codon alignments.. Nucleic Acids Res.

[40] Ruiz-Echevarria MJ, Peltz SW (2000). The RNA binding protein Pub1 modulates the stability of transcripts containing upstream open reading frames.. Cell.

[41] Hornstein E, Harel H, Levy G, Meyuhas O (1999). Overexpression of poly(A)-binding protein down-regulates the translation or the abundance of its own mRNA.. FEBS Lett.

[42] Bailey TL, Elkan C (1994). Fitting a mixture model by expectation maximization to discover motifs in biopolymers.. Proc Int Conf Intell Syst Mol Bio..

[43] Zhang S, Ruiz-Echevarria MJ, Quan Y, Peltz SW (1995). Identification and characterization of a sequence motif involved in nonsense-mediated mRNA decay.. Mol Cell Biol.

[44] Fairbrother WG, Yeh RF, Sharp PA, Burge CB (2002). Predictive identification of exonic splicing enhancers in human genes.. Science.

[45] Kim YK, Furic L, Desgroseillers L, Maquat LE (2005). Mammalian Staufen1 recruits Upf1 to specific mRNA 3′UTRs so as to elicit mRNA decay.. Cell.

[46] Kim YK, Furic L, Parisien M, Major F, DesGroseillers L (2007). Staufen1 regulates diverse classes of mammalian transcripts.. EMBO J.

[47] Cuccurese M, Russo G, Russo A, Pietropaolo C (2005). Alternative splicing and nonsense-mediated mRNA decay regulate mammalian ribosomal gene expression.. Nucleic Acids Res.

[48] Vilardell J, Chartrand P, Singer RH, Warner JR (2000). The odyssey of a regulated transcript.. RNA.

[49] McGlincy NJ, Smith CWJ (2008). Alternative splicing resulting in nonsense-mediated mRNA decay: what is the meaning of nonsense?. Trends Biochem Sci.

[50] Smyth GK, Gentleman R, Carey V, Du-doit S, R Irizarry WH (2005). Limma: linear models for microarray data.. Bioinformatics and Computational Biology Solutions using R and Bioconductor..

[51] Gentleman RC, Carey VJ, Bates DM, Bolstad B, Dettling M (2004). Bioconductor: open software development for computational biology and bioinformatics.. Genome Biology.

[52] Ritchie ME, Silver J, Oshlack A, Holmes M, Diyagama D (2007). A comparison of background correction methods for two-colour microarrays.. Bioinformatics.

[53] Lange K (1994). An adaptive barrier method for convex programming.. Methods and Applications of Analysis.

[54] Bates DM, Watts DG (1988). Nonlinear Regression Analysis and Its Applications..

[55] Hothorn T, Hornik K, van de Wiel MA, Zeileis A (2006). A Lego System for Conditional Inference.. The American Statistician.

