# Supplementary material for: Genome-Wide Identification of Alternative Splice Forms Down-Regulated by Nonsense-Mediated mRNA Decay in Drosophila
Source: PLoS Genet. 2009 Jun 19;5(6):e1000525. doi: 10.1371/journal.pgen.1000525 (PMC2689934; doi:10.1371/journal.pgen.1000525)
Supplement: Figure S1 — Overlayed MA plots for upf1. MA plot of the 3 normalized upf1 arrays. The three arrays have been superimposed on the same plot. The red line is a lowess line, and the blue line is M = 0. The yellow/green points are probes targeting upf1/upf2 and the blue/purple points are positive (present)/negative (absent) control probes. (0.79 MB PDF) [file pgen.1000525.s001.pdf]

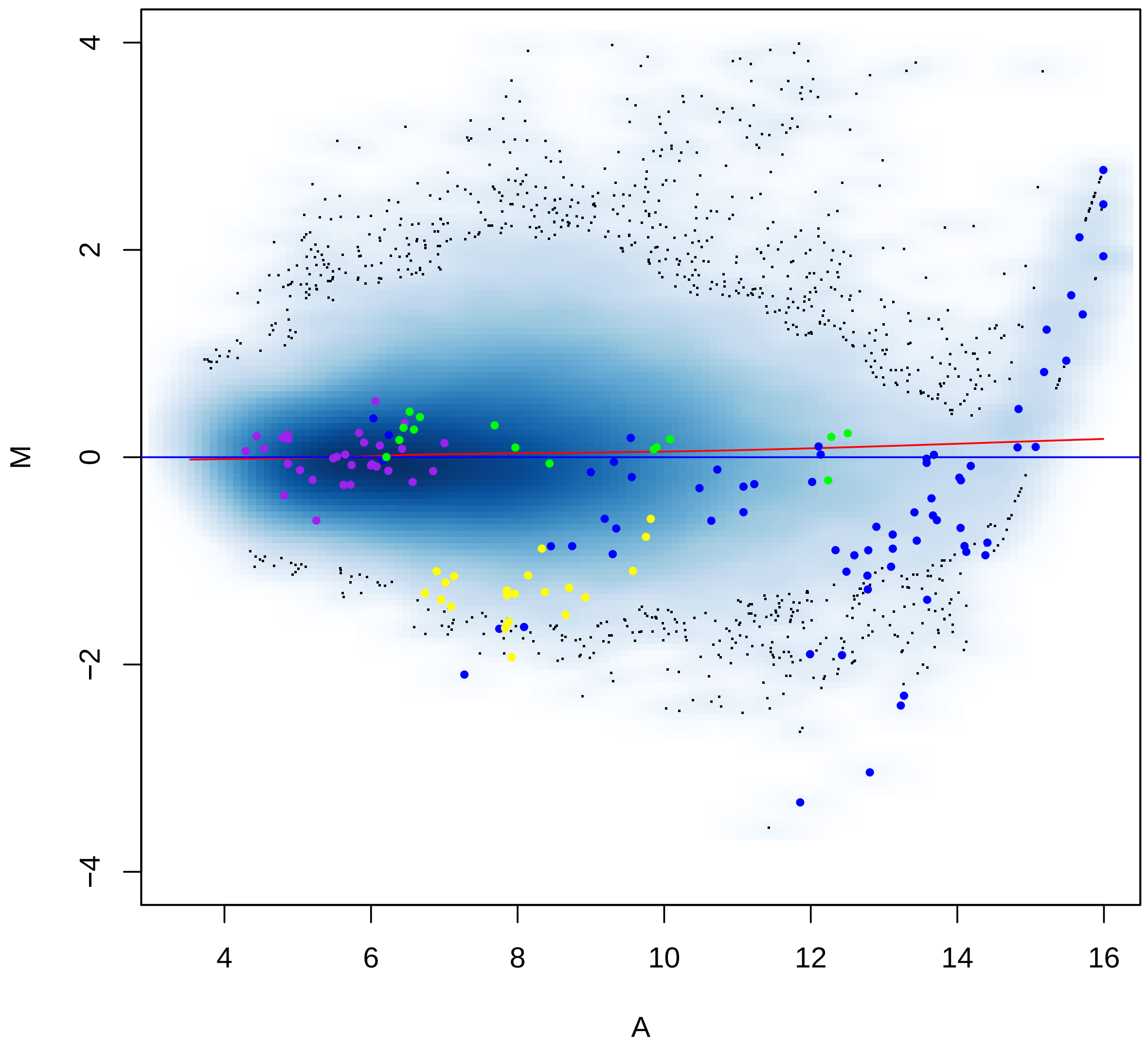

Figure S1. Overlayed MA plots for *upf1*. MA plot of the 3 normalized *upf1* arrays. The three arrays have been superimposed on the same plot. The red line is a lowess line, and the blue line is $M = 0$. The yellow/green points are probes targeting *upf1/upf2* and the blue/purple points are positive (present)/negative (absent) control probes.
