# Supplementary figures and images for: Genome-Wide Identification of Alternative Splice Forms Down-Regulated by Nonsense-Mediated mRNA Decay in Drosophila
Source: PLoS Genet. 2009 Jun 19;5(6):e1000525. doi: 10.1371/journal.pgen.1000525 (PMC2689934; doi:10.1371/journal.pgen.1000525)

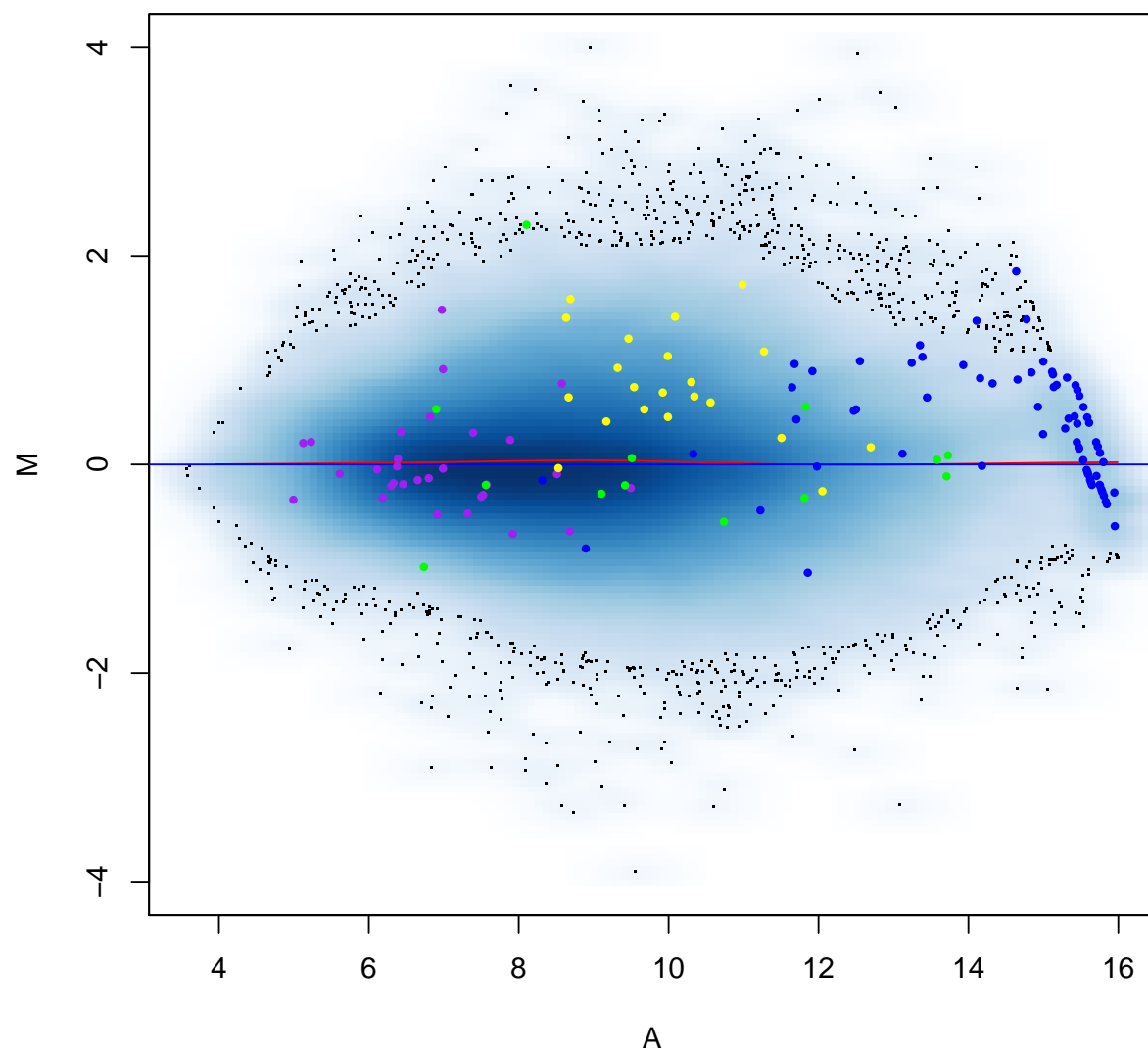

**Figure S2. Overlaid MA plots for *upf2*.** As Figure S1, but for *upf2*.

Supplement: Figure S2 — Overlayed MA plots for upf2. As Figure S1, but for upf2. (0.79 MB PDF) [file pgen.1000525.s002.pdf]

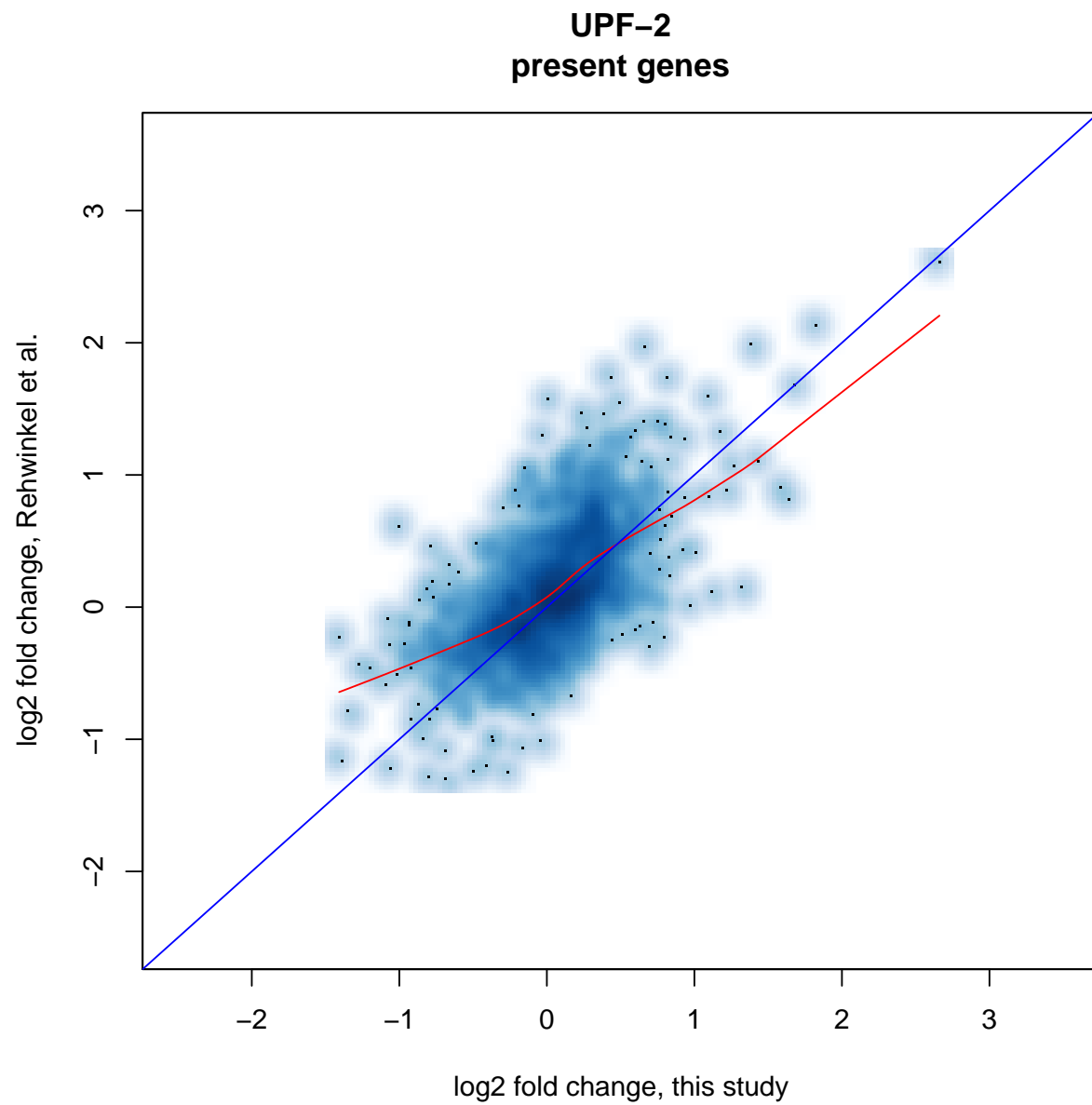

**Figure S6. Smoothed scatterplot of *upf2* and Affymetrix data.** As Figure S5, but for *upf2*.

Supplement: Figure S6 — Smoothed scatterplot of upf2 and Affymetrix data. As Figure S5, but for upf2. (0.68 MB PDF) [file pgen.1000525.s006.pdf]

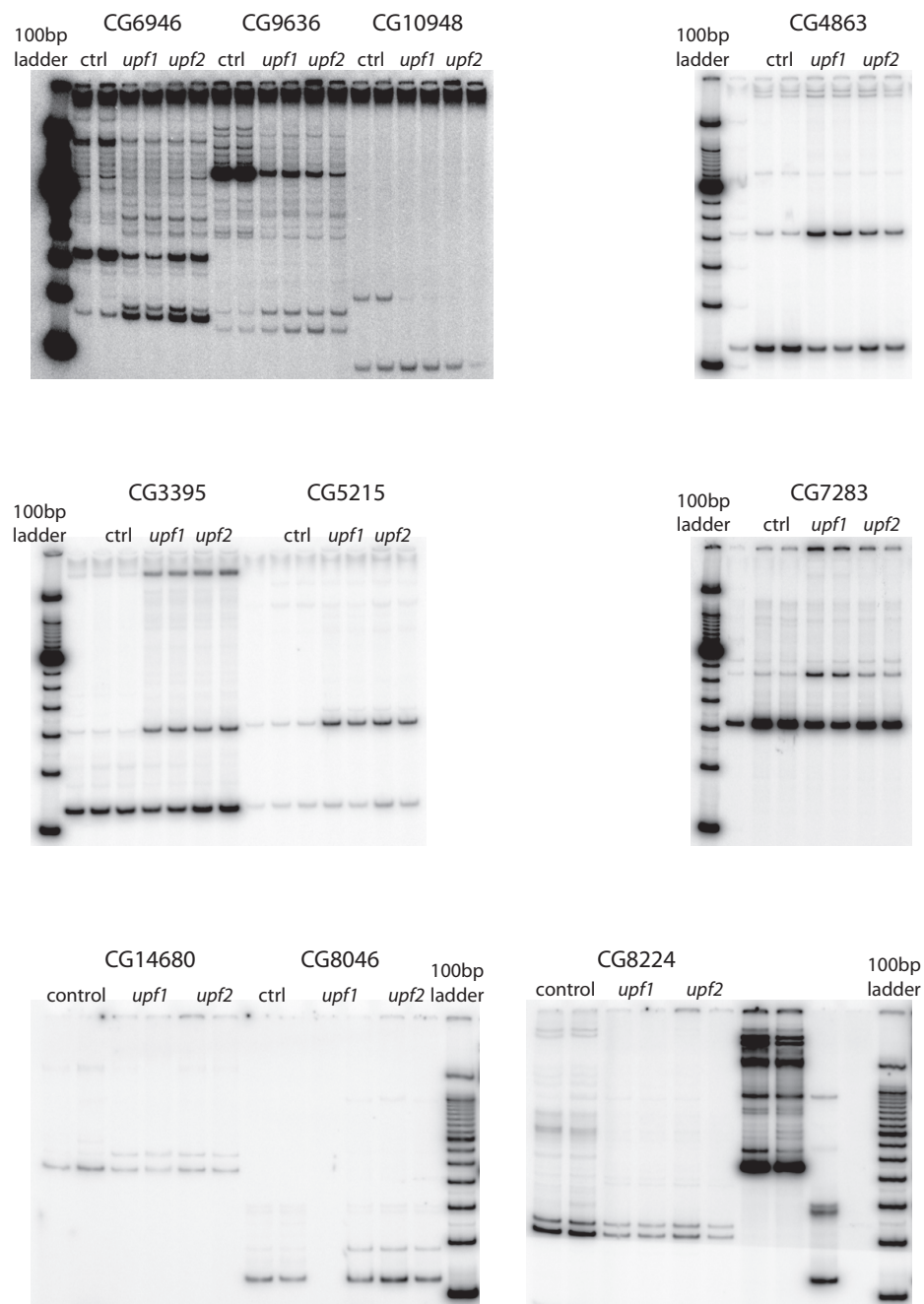

**Figure S8. RT-PCR validation of NMD.** Full gels from the RT-PCR validation described in main Figure 1.

Supplement: Figure S8 — RT-PCR validation of NMD. Full gels from the RT-PCR validation described in main Figure 1. (1.45 MB PDF) [file pgen.1000525.s008.pdf]

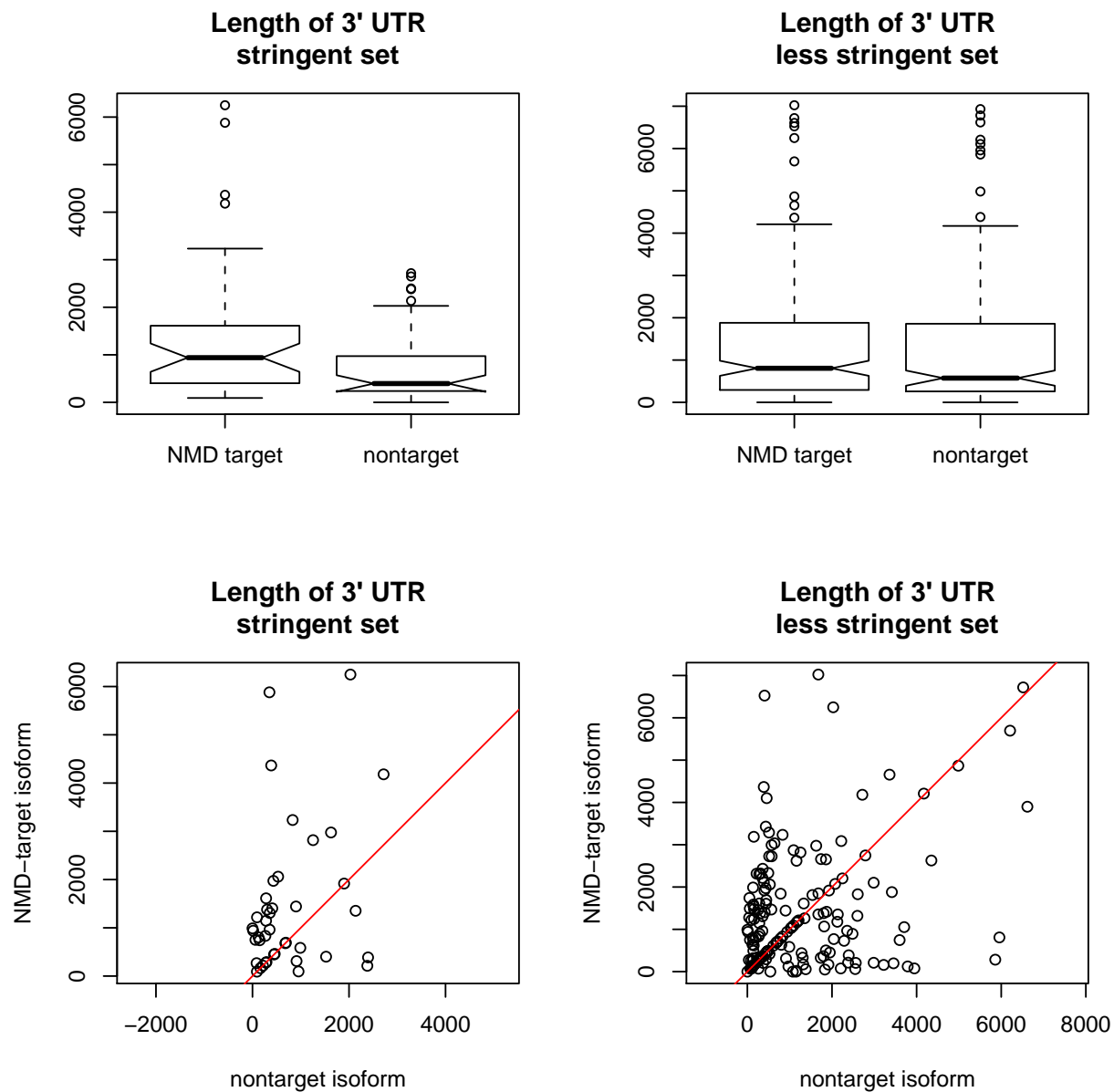

**Figure S13. Length of 3' UTR.** As Figure S9 for the feature “length of 3' UTR.”

Supplement: Figure S13 — Length of 3′ UTR. As Figure S9 for the feature “length of 3′ UTR.” (0.05 MB PDF) [file pgen.1000525.s013.pdf]

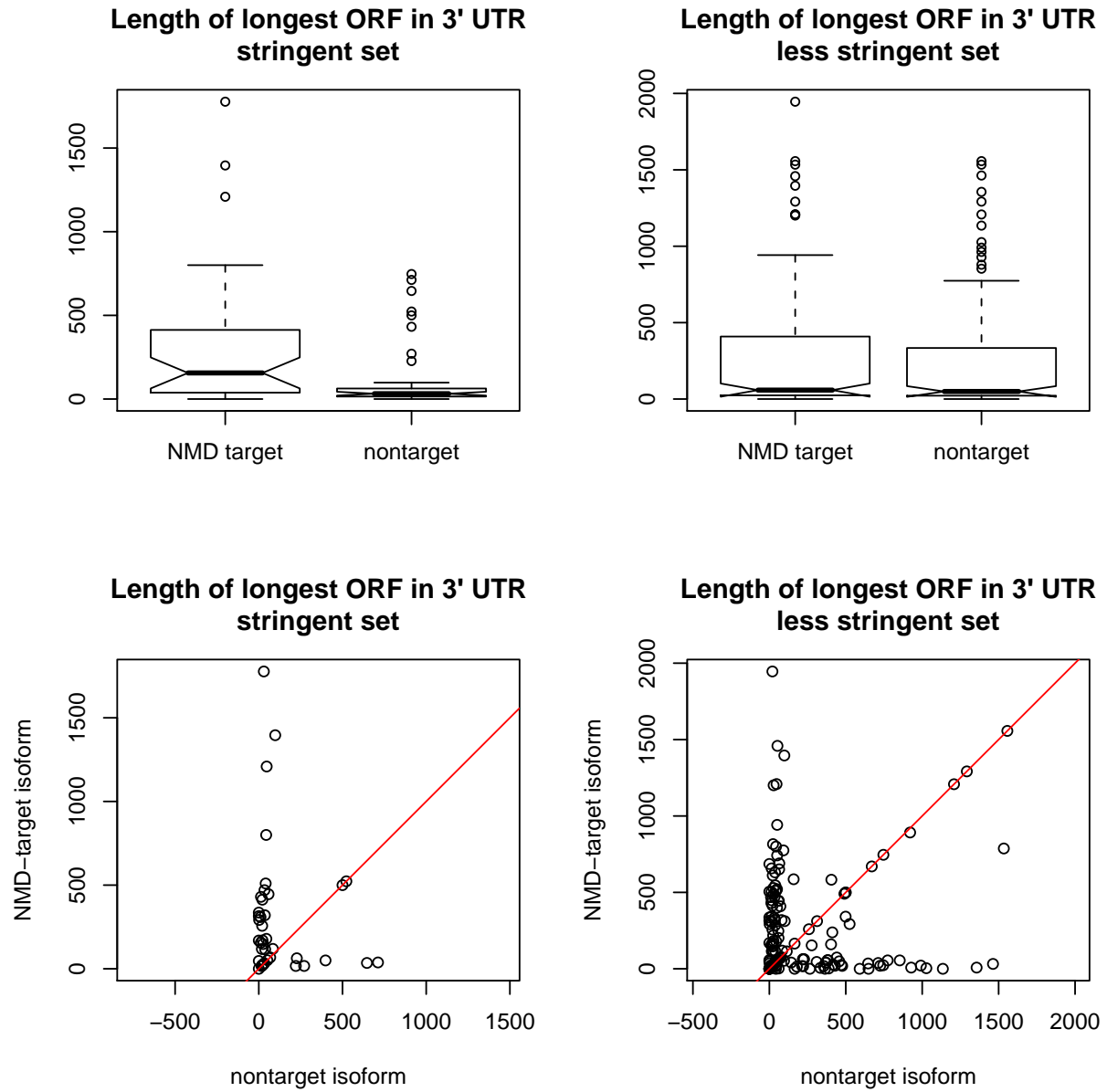

**Figure S15. Length of longest ORF in 3' UTR.** As Figure S9 for the feature “length of longest ORF in 2' UTR.”

Supplement: Figure S15 — Length of longest ORF in 3′ UTR. As Figure S9 for the feature “length of longest ORF in 2′ UTR.” (0.05 MB PDF) [file pgen.1000525.s015.pdf]

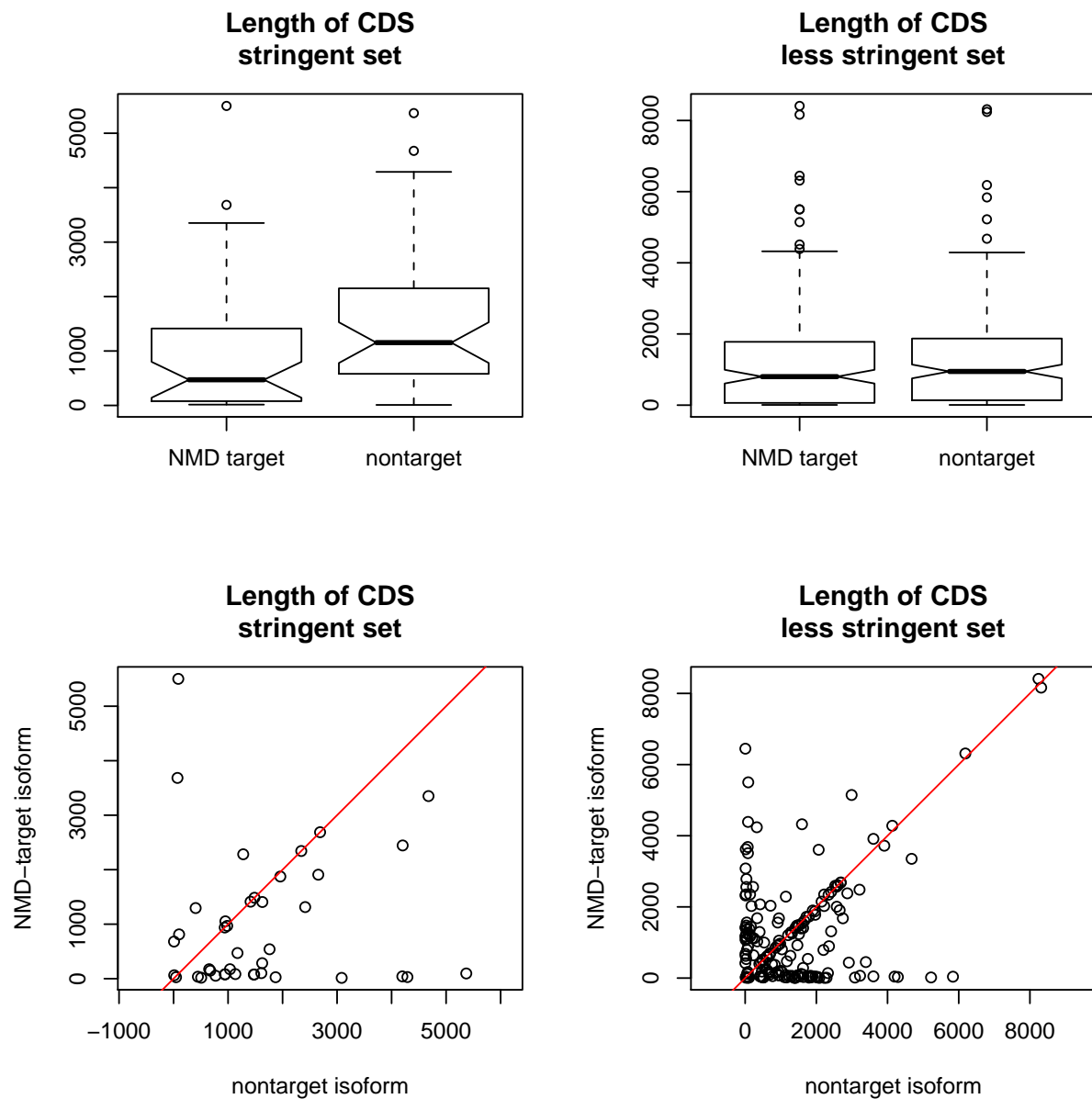

**Figure S19. Length of CDS.** As Figure S9 for the feature “length of CDS.”

Supplement: Figure S19 — Length of CDS. As Figure S9 for the feature “length of CDS.” (0.05 MB PDF) [file pgen.1000525.s019.pdf]
