# Supplementary material for: Genome-Wide Identification of Alternative Splice Forms Down-Regulated by Nonsense-Mediated mRNA Decay in Drosophila
Source: PLoS Genet. 2009 Jun 19;5(6):e1000525. doi: 10.1371/journal.pgen.1000525 (PMC2689934; doi:10.1371/journal.pgen.1000525)
Supplement: Figure S3 — Individual MA plots for upf1. MA plots of the 3 normalized upf1 arrays side by side. Lines and colorscheme are as in Figure S1. (2.28 MB PDF) [file pgen.1000525.s003.pdf]

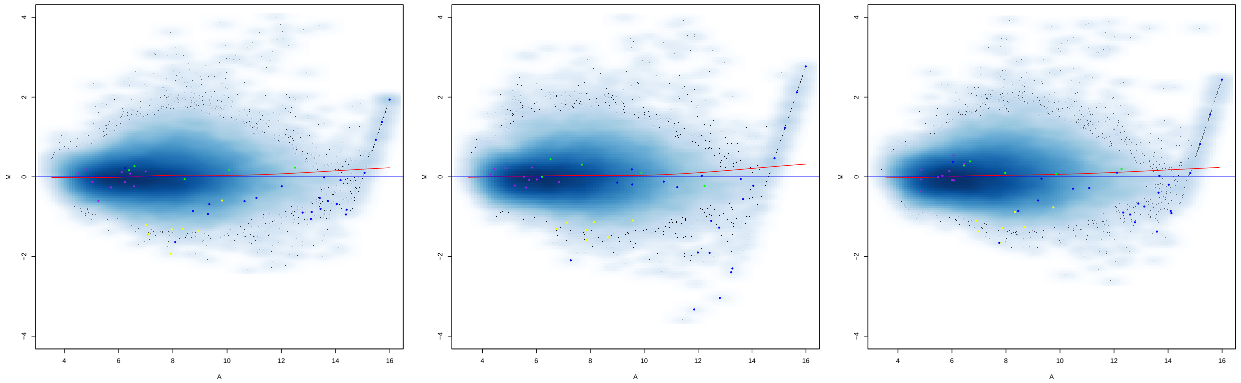

Figure S3. Individual MA plots for *upf1*. MA plots of the 3 normalized *upf1* arrays side by side. Lines and colorscheme are as in Figure S1.
