# Supplementary material for: Genome-Wide Identification of Alternative Splice Forms Down-Regulated by Nonsense-Mediated mRNA Decay in Drosophila
Source: PLoS Genet. 2009 Jun 19;5(6):e1000525. doi: 10.1371/journal.pgen.1000525 (PMC2689934; doi:10.1371/journal.pgen.1000525)
Supplement: Figure S5 — Smoothed scatterplot of upf1 and Affymetrix data. Smoothed scatterplot of gene level log2 fold changes from this study versus Rehwinkel et al., from the upf1 arrays. Rehwinkel's normalized data was obtained from Array Express E-MEXP-202. Genes that were labelled as absent in either study were removed. The blue line is y = x and the red line is lowess smoother. Our gene level fold changes were obtained by averaging all probes annotated as being constitutive. Using only constitutive exon probes instead of constitutive exon and junction probes did not qualitatively change the scatterplot. (0.68 MB PDF) [file pgen.1000525.s005.pdf]

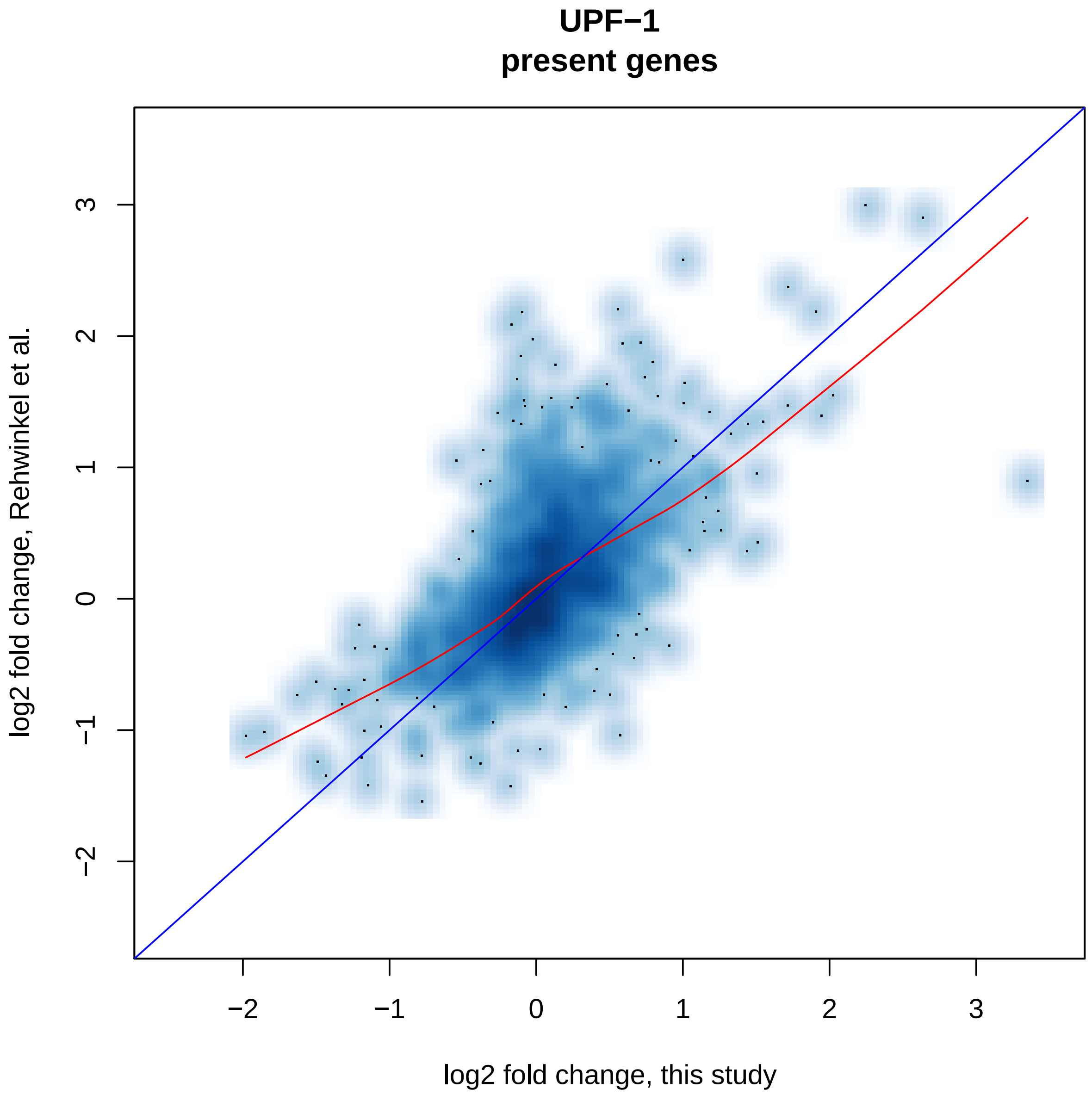

Figure S5. Smoothed scatterplot of *upf1* and Affymetrix data. Smoothed scatterplot of gene level $\log_2$ fold changes from this study versus Rehwinkel et al., from the *upf1* arrays. Rehwinkel's normalized data was obtained from Array Express E-MEXP-202. Genes that were labelled as absent in either study were removed. The blue line is $y = x$ and the red line is lowess smoother. Our gene level fold changes were obtained by averaging all probes annotated as being constitutive. Using only constitutive exon probes instead of constitutive exon and junction probes did not qualitatively change the scatterplot.
