# Supplementary material for: Genome-Wide Identification of Alternative Splice Forms Down-Regulated by Nonsense-Mediated mRNA Decay in Drosophila
Source: PLoS Genet. 2009 Jun 19;5(6):e1000525. doi: 10.1371/journal.pgen.1000525 (PMC2689934; doi:10.1371/journal.pgen.1000525)
Supplement: Figure S7 — RT-PCR shows NMD in RpS9 and RpL3. RT-PCR shows NMD-target isoforms of RpS9 and RpL3. Neither gene could be deconvolved by our array analysis. (0.51 MB PDF) [file pgen.1000525.s007.pdf]

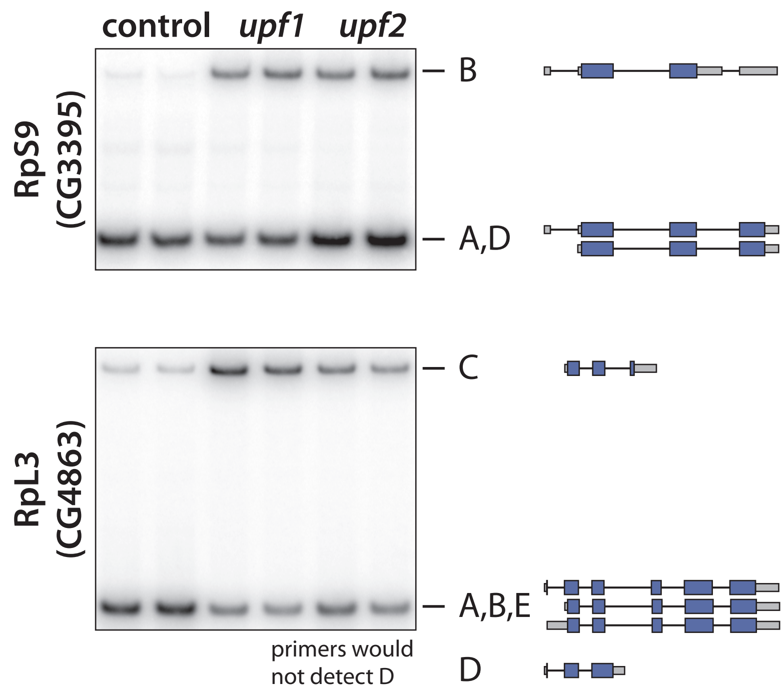

Figure S7. RT-PCR shows NMD in RpS9 and RpL3. RT-PCR shows NMD-target isoforms of RpS9 and RpL3. Neither gene could be deconvolved by our array analysis.
