# Supplementary material for: Genome-Wide Identification of Alternative Splice Forms Down-Regulated by Nonsense-Mediated mRNA Decay in Drosophila
Source: PLoS Genet. 2009 Jun 19;5(6):e1000525. doi: 10.1371/journal.pgen.1000525 (PMC2689934; doi:10.1371/journal.pgen.1000525)
Supplement: Figure S9 — Length of 5′ UTR. Top right and top left plots are boxplots comparing the set of NMD-target mRNAs to the set of NMD nontarget mRNAs from the same genes, for both the stringent and the less stringent set of upf1-affected genes. Bottom right and bottom left are scatterplots between mRNAs of the same gene, labelled as either NMD-target or NMD nontarget, for both the stringent and the less stringent set of genes. The feature in question is the “length of 5′ UTR.” The scatterplots have an aspect ratio of 1. (0.06 MB PDF) [file pgen.1000525.s009.pdf]

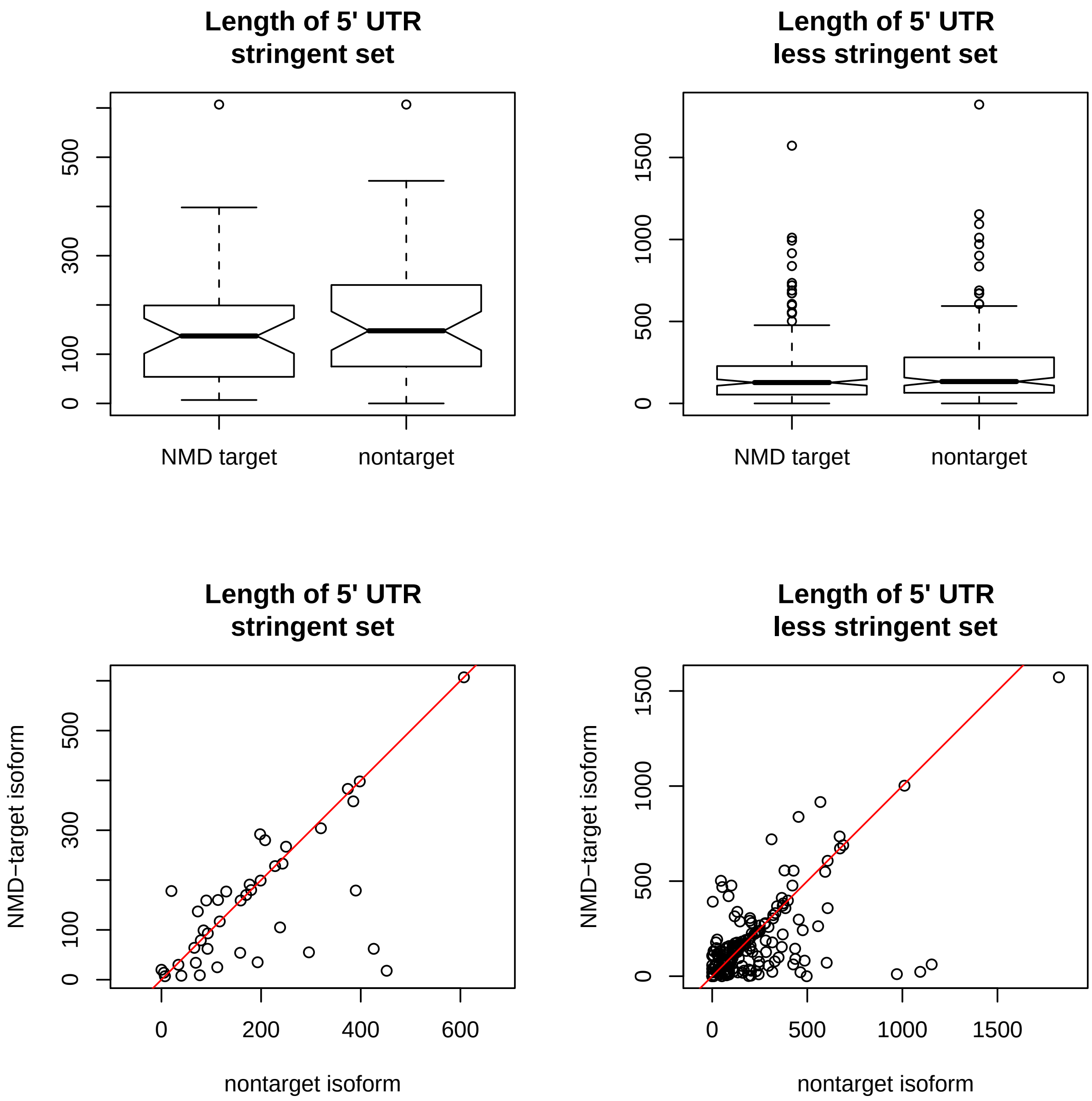

Figure S9. Length of 5' UTR. Top right and top left plots are boxplots comparing the set of NMD-target mRNAs to the set of NMD nontarget mRNAs from the same genes, for both the stringent and the less stringent set of *upf1*-affected genes. Bottom right and bottom left are scatterplots between mRNAs of the same gene, labelled as either NMD-target or NMD nontarget, for both the stringent and the less stringent set of genes. The feature in question is the “length of 5' UTR.” The scatterplots have an aspect ratio of 1.
