# Supplementary material for: Genome-Wide Identification of Alternative Splice Forms Down-Regulated by Nonsense-Mediated mRNA Decay in Drosophila
Source: PLoS Genet. 2009 Jun 19;5(6):e1000525. doi: 10.1371/journal.pgen.1000525 (PMC2689934; doi:10.1371/journal.pgen.1000525)
Supplement: Figure S10 — Length of longest ORF in the 5′ UTR. As Figure S9 for the feature “length of longest ORF in 5′ UTR.” The bottom left scatterplot has been jittered. (0.05 MB PDF) [file pgen.1000525.s010.pdf]

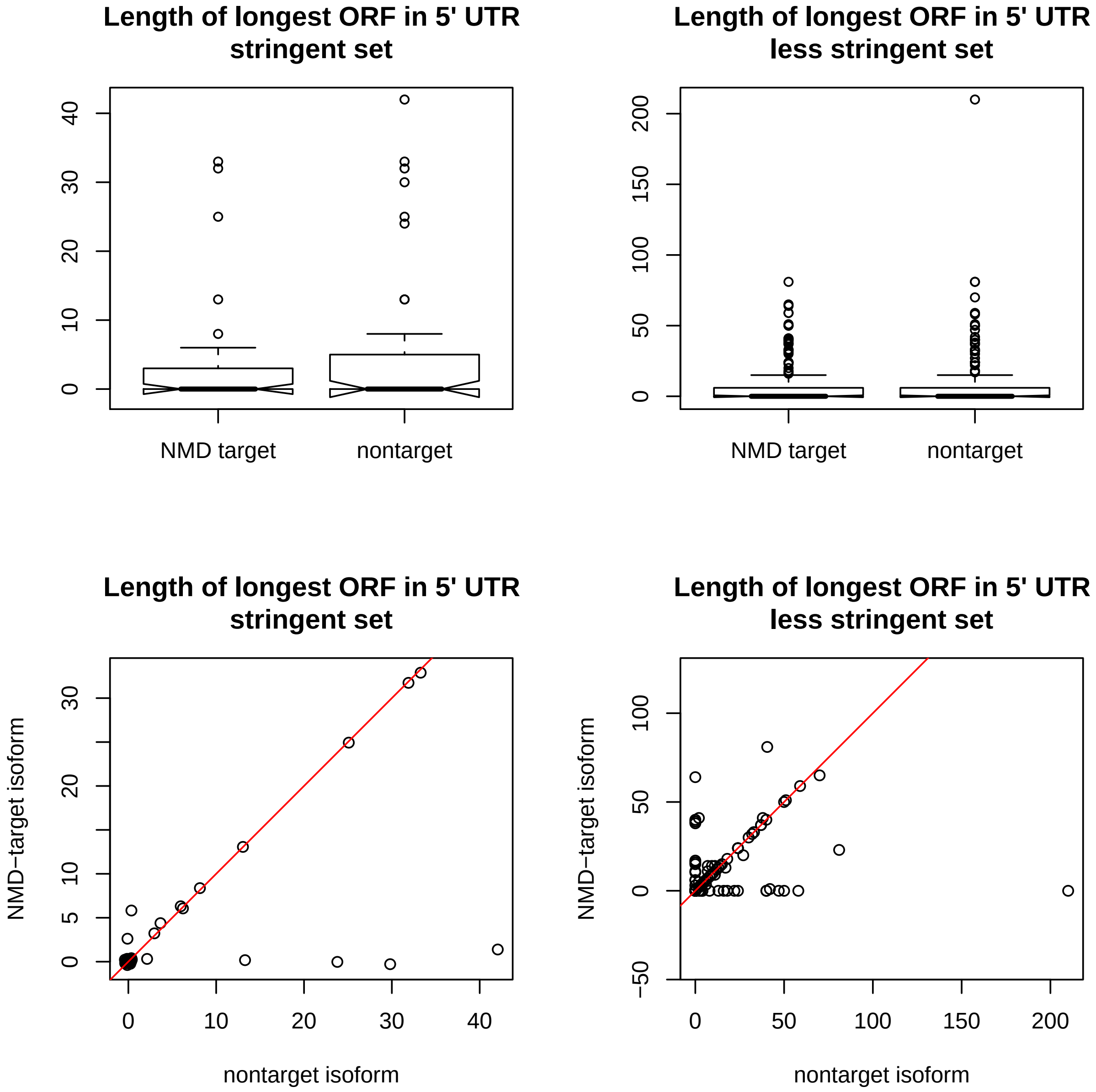

Figure S10. Length of longest ORF in the 5' UTR. As Figure S9 for the feature "length of longest ORF in 5' UTR." The bottom left scatterplot has been jittered.
