# Supplementary material for: Genome-Wide Identification of Alternative Splice Forms Down-Regulated by Nonsense-Mediated mRNA Decay in Drosophila
Source: PLoS Genet. 2009 Jun 19;5(6):e1000525. doi: 10.1371/journal.pgen.1000525 (PMC2689934; doi:10.1371/journal.pgen.1000525)
Supplement: Figure S12 — Length of longest A-rich region in 5′ UTR. As Figure S9 for the feature “length of longest A-rich region in 5′ UTR.” The bottom left scatterplot has been jittered. (0.05 MB PDF) [file pgen.1000525.s012.pdf]

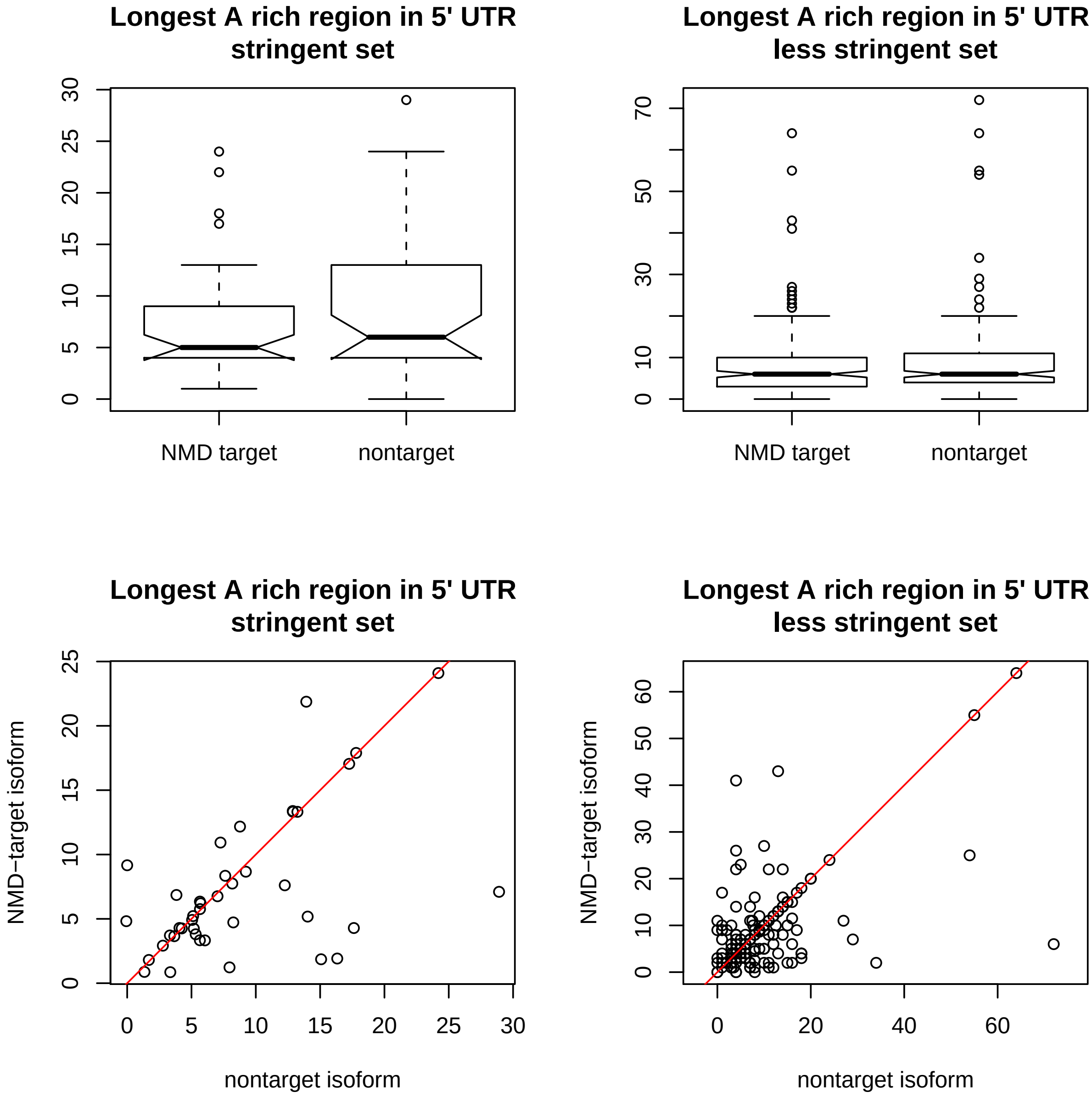

Figure S12. Length of longest A-rich region in 5' UTR. As Figure S9 for the feature "length of longest A-rich region in 5' UTR." The bottom left scatterplot has been jittered.
