# Supplementary material for: Genome-Wide Identification of Alternative Splice Forms Down-Regulated by Nonsense-Mediated mRNA Decay in Drosophila
Source: PLoS Genet. 2009 Jun 19;5(6):e1000525. doi: 10.1371/journal.pgen.1000525 (PMC2689934; doi:10.1371/journal.pgen.1000525)
Supplement: Figure S14 — Number of introns in 3′ UTR. As Figure S9 for the feature “number of introns in 3′ UTR.” Both the bottom right and the bottom left scatterplots have been jittered. (0.05 MB PDF) [file pgen.1000525.s014.pdf]

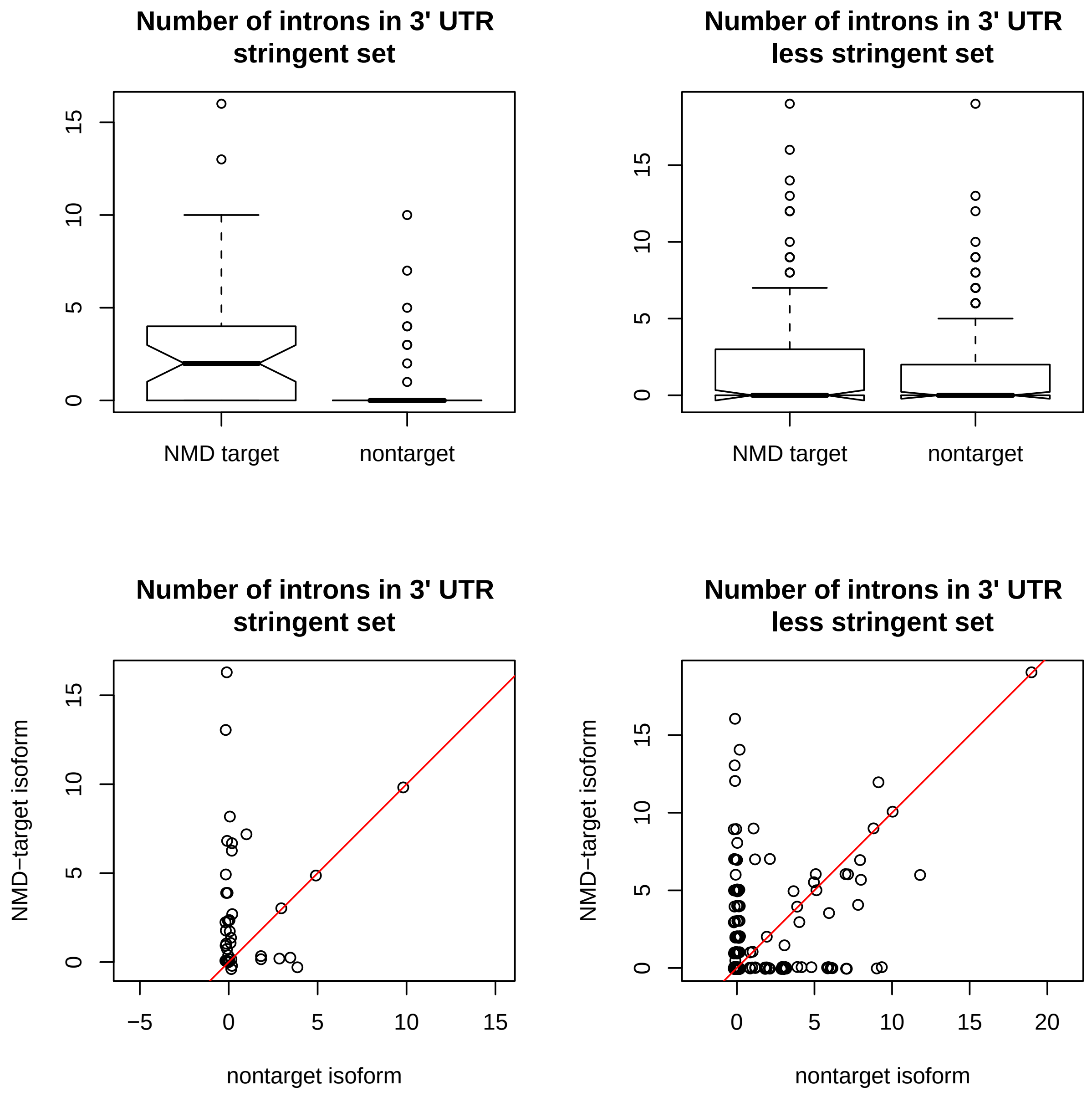

Figure S14. Number of introns in 3' UTR. As Figure S9 for the feature "number of introns in 3' UTR." Both the bottom right and the bottom left scatterplots has been jittered.
