# Supplementary material for: Genome-Wide Identification of Alternative Splice Forms Down-Regulated by Nonsense-Mediated mRNA Decay in Drosophila
Source: PLoS Genet. 2009 Jun 19;5(6):e1000525. doi: 10.1371/journal.pgen.1000525 (PMC2689934; doi:10.1371/journal.pgen.1000525)
Supplement: Figure S17 — Length of longest A-rich region in 3′ UTR. As Figure S9 for the feature “length of longest A-rich region in 3′ UTR.” (0.05 MB PDF) [file pgen.1000525.s017.pdf]

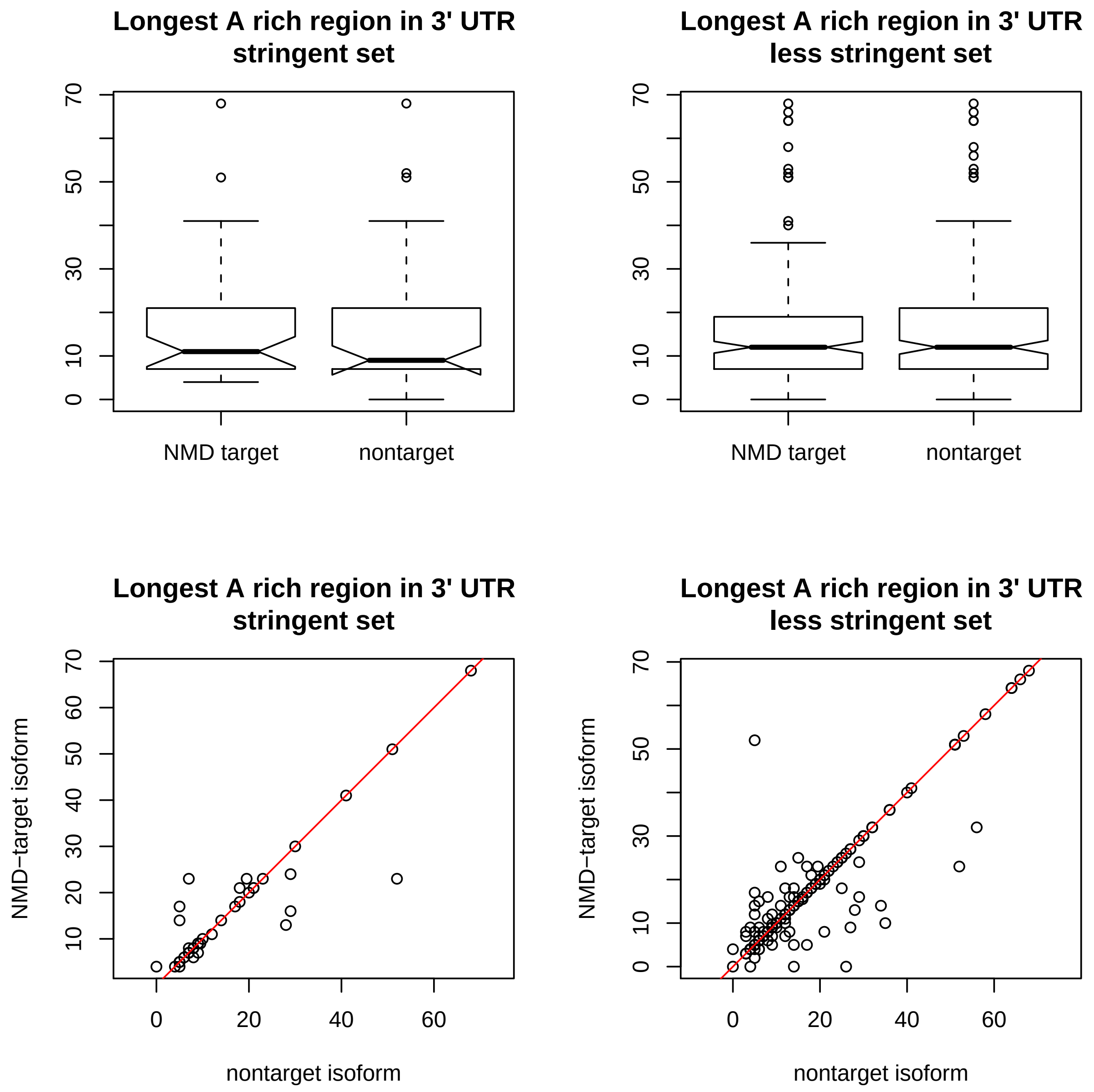

Figure S17. Length of longest A-rich region in 3’ UTR. As Figure S9 for the feature “length of longest A-rich region in 3’ UTR.”
