# Supplementary material for: Genome-Wide Identification of Alternative Splice Forms Down-Regulated by Nonsense-Mediated mRNA Decay in Drosophila
Source: PLoS Genet. 2009 Jun 19;5(6):e1000525. doi: 10.1371/journal.pgen.1000525 (PMC2689934; doi:10.1371/journal.pgen.1000525)
Supplement: Figure S18 — Number of introns in transcript. As Figure S9 for the feature “number of introns in transcript.” The bottom left scatterplot has been jittered. (0.05 MB PDF) [file pgen.1000525.s018.pdf]

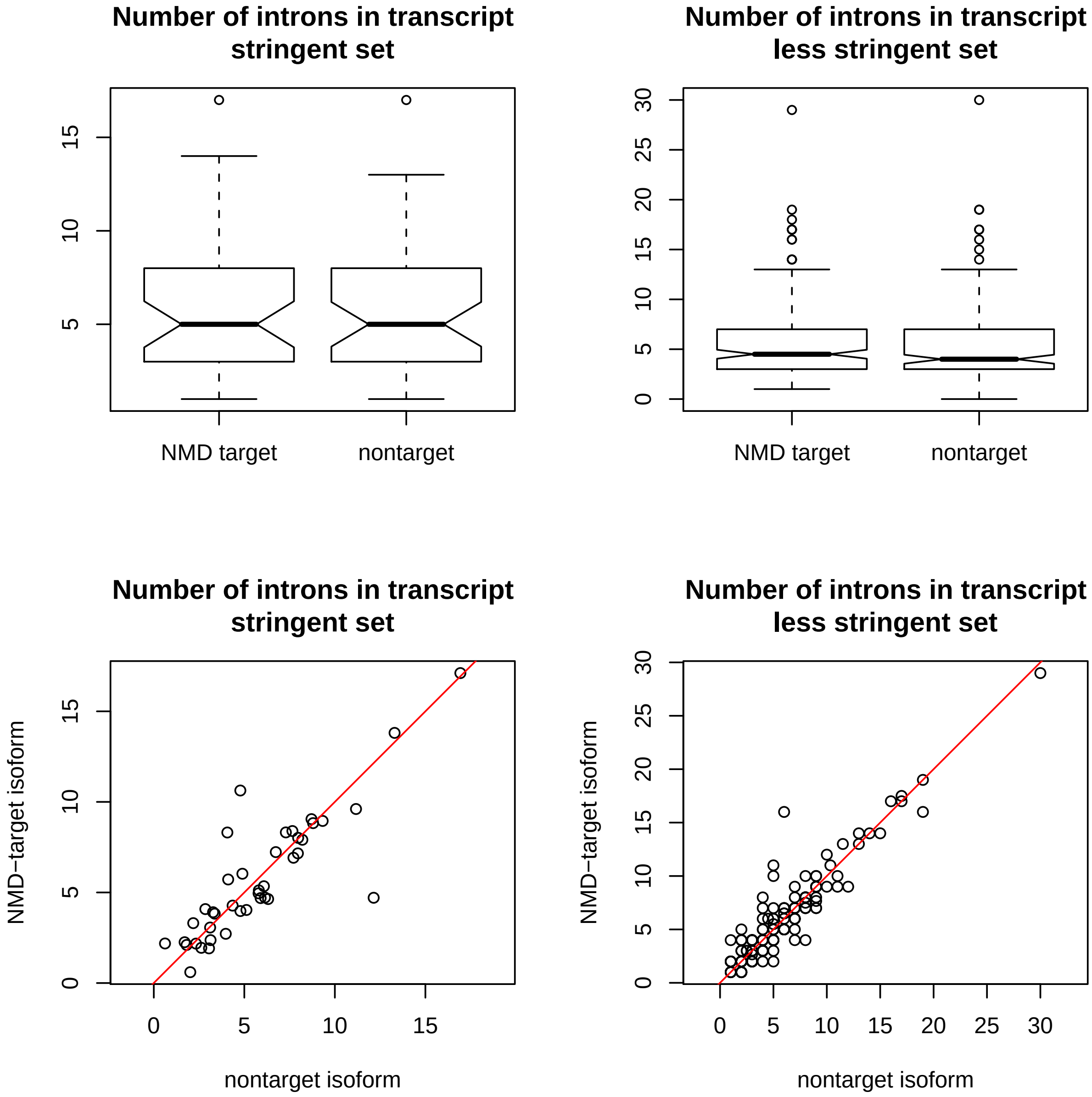

Figure S18. Number of introns in transcript. As Figure S9 for the feature "number of introns in transcript." The bottom left scatterplot has been jittered.
