# Supplementary material for: Genome-Wide Identification of Alternative Splice Forms Down-Regulated by Nonsense-Mediated mRNA Decay in Drosophila
Source: PLoS Genet. 2009 Jun 19;5(6):e1000525. doi: 10.1371/journal.pgen.1000525 (PMC2689934; doi:10.1371/journal.pgen.1000525)
Supplement: Figure S21 — Distance from stop codon to final intron. As Figure S9 for the feature “distance from stop codon to final intron.” CG11100 did not have any introns and is not assigned a value. (0.05 MB PDF) [file pgen.1000525.s021.pdf]

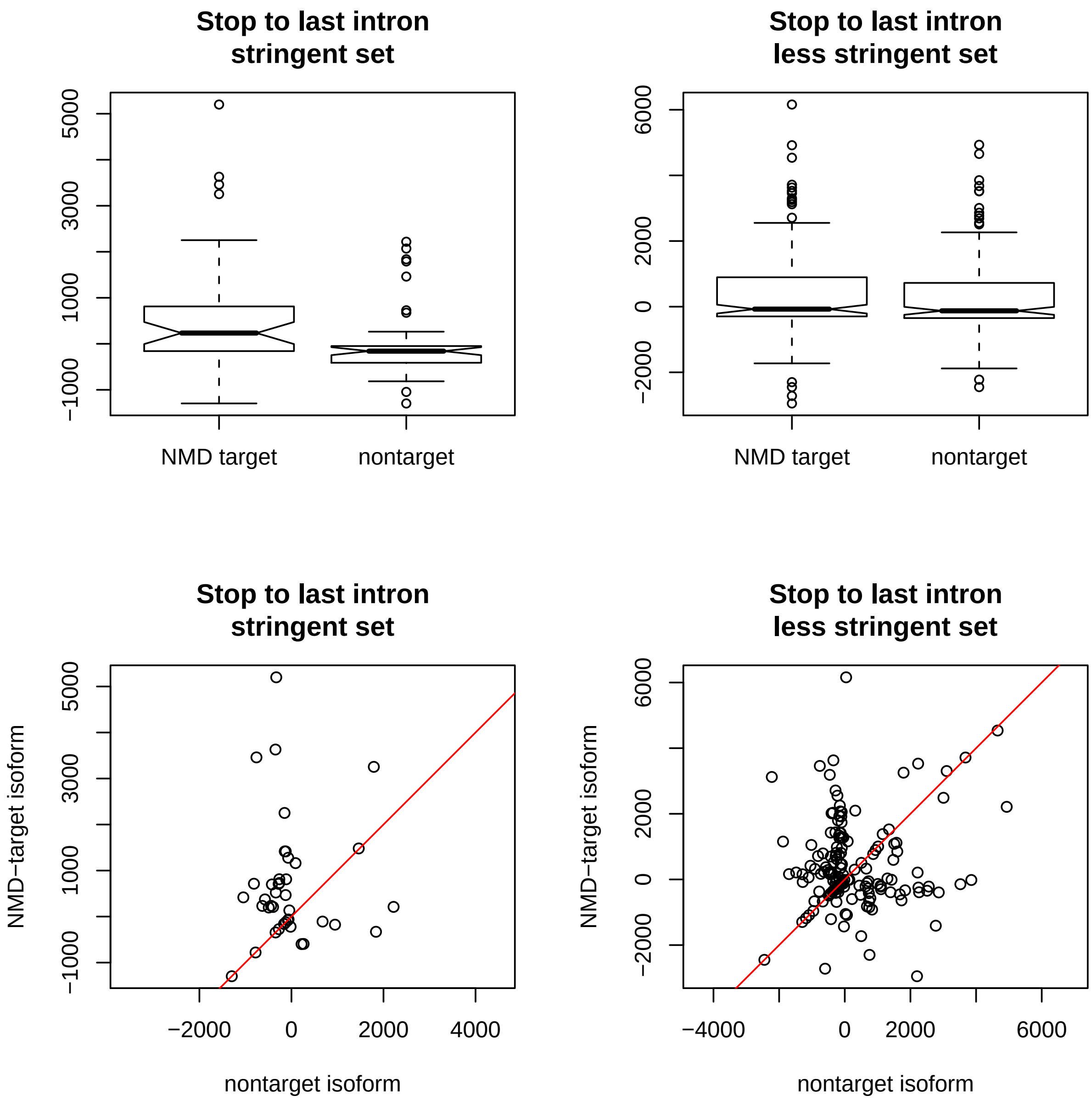

Figure S21. Distance from stop codon to final intron. As Figure S9 for the feature “distance from stop codon to final intron.” CG11100 did not have any introns and is not assigned a value.
