# Supplementary material for: Genome-Wide Identification of Alternative Splice Forms Down-Regulated by Nonsense-Mediated mRNA Decay in Drosophila
Source: PLoS Genet. 2009 Jun 19;5(6):e1000525. doi: 10.1371/journal.pgen.1000525 (PMC2689934; doi:10.1371/journal.pgen.1000525)
Supplement: Figure S22 — Motifs found by MEME. A. Overrepresented motifs found in the 3′ UTRs of NMD-target mRNAs. MEME was used to search for motifs with a width of 6–16 nt occurring zero or one times per sequence. All motifs appear to be repetitive sequence. B. Overrepresented motifs found in the 3′ UTRs of the 27 NMD-target mRNAs with an early stop codon. Motifs 2, 3, and 5 match the motifs in (A); motifs 1 and 4 appear similar to known RARRAR splicing enhancers. (0.47 MB PDF) [file pgen.1000525.s022.pdf]

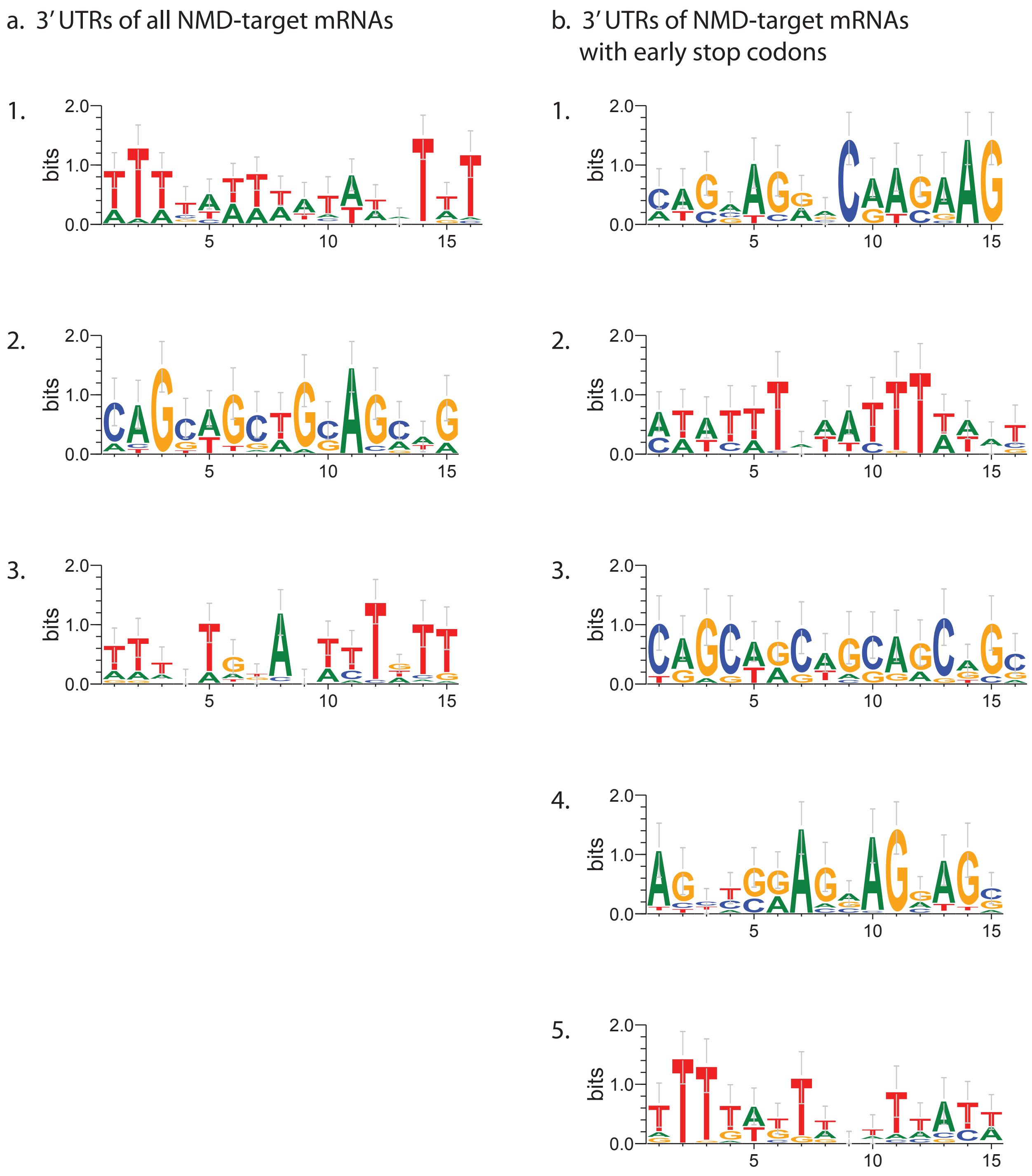

Figure S22. Motifs found by MEME. a. Overrepresented motifs found in the 3' UTRs of NMD-target mRNAs. MEME was used to search for motifs with a width of 6-16 nt occurring zero or one times per sequence. All motifs appear to be repetitive sequence. b. Overrepresented motifs found in the 3' UTRs of the 27 NMD-target mRNAs with an early stop codon. Motifs 2, 3, and 5 match the motifs in (a); motifs 1 and 4 appear similar to known RARRAR splicing enhancers.
