# Supplementary material for: Genome-Wide Identification of Alternative Splice Forms Down-Regulated by Nonsense-Mediated mRNA Decay in Drosophila
Source: PLoS Genet. 2009 Jun 19;5(6):e1000525. doi: 10.1371/journal.pgen.1000525 (PMC2689934; doi:10.1371/journal.pgen.1000525)
Supplement: Table S1 — Number of AUG (start) codons in 5′ UTR. Tables (a) and (b) show an unpaired comparison between the NMD-target mRNAs and the NMD nontarget mRNAs for the stringent and the less stringent set of upf1-affected genes, for the feature “number of AUG (start) codons in 5′ UTR.” Tables (c) and (d) show a paired comparison between the two sets of mRNAs, for the stringent and the less stringent set of genes. (0.04 MB PDF) [file pgen.1000525.s023.pdf]

Table S1. Number of AUG (start) codons in 5' UTR

(a) Stringent set

value	NMD target	NMD nontarget
0	29	30
1	8	7
2	2	2
3	1	2
5	1	2
6	0	1

(b) Less stringent set

value	NMD target	NMD nontarget
0	119	122
1	42	37
2	10	8
3	13	10
4	1	5
5	7	6
6	1	1
7	2	4
8	1	1
9	0	2
11	1	0
12	1	1
15	0	1

(c) Stringent set

	NMD nontarget					
	0	1	2	3	5	6
0	25	1	0	1	1	1
1	1	6	0	1	0	0
2	0	0	2	0	0	0
3	1	0	0	0	0	0
5	0	0	0	0	1	0

(d) Less stringent set

	NMD nontarget												
	0	1	1.5	2	3	4	4.5	5	6	7	9	12	15
0	93	7	0	0	0	2	1	1	1	2	1	0	1
0.5	0	1	0	0	0	0	0	0	0	0	0	0	0
0.67	1	0	0	0	0	0	0	0	0	0	0	0	0
1	7	25	0	1	2	1	0	0	0	0	0	0	0
2	0	2	0	5	2	0	0	0	0	0	0	0	0
3	3	1	1	3	3	0	0	1	0	0	0	0	0
4	0	0	0	0	0	1	0	0	0	0	0	0	0
5	1	1	0	0	0	1	0	4	0	0	0	0	0
6	1	0	0	0	0	0	0	0	0	0	0	0	0
7	0	0	0	0	0	0	0	0	0	2	0	0	0
8	0	0	0	0	0	1	0	0	0	0	0	0	0
11.5	0	0	0	0	0	0	0	0	0	0	0	1	0

Tables (a) and (b) show an unpaired comparison between the NMD-target mRNAs and the NMD nontarget mRNAs for the stringent and the less stringent set of *upf1*-affected genes, for the feature "number of AUG (start) codons in 5' UTR." Tables (c) and (d) show a paired comparison between the two sets of mRNAs, for the stringent and the less stringent set of genes.
