# Supplementary material for: Genome-Wide Identification of Alternative Splice Forms Down-Regulated by Nonsense-Mediated mRNA Decay in Drosophila
Source: PLoS Genet. 2009 Jun 19;5(6):e1000525. doi: 10.1371/journal.pgen.1000525 (PMC2689934; doi:10.1371/journal.pgen.1000525)
Supplement: Table S2 — Number of introns in 5′ UTR. As Table S1, but for the feature “number of introns in 5′ UTR.” (0.03 MB PDF) [file pgen.1000525.s024.pdf]

Table S2. Number of introns in 5' UTR

(a) Stringent set

value	NMD target	NMD nontarget
0	28	30
1	12	12
2	1	2

(b) Less stringent set

value	NMD target	NMD nontarget
0	133	142
1	57	47
2	6	7
3	2	2

(c) Stringent set

	NMD nontarget			
	0	0.5	1	2
0	20	1	6	1
1	7	0	5	0
2	0	0	0	1

(d) Less stringent set

	NMD nontarget						
	0	0.25	0.5	0.67	1	2	3
0	100	1	2	0	15	3	1
0.5	3	0	0	0	0	0	0
0.67	0	0	0	0	2	0	0
1	21	0	1	1	22	1	0
2	2	0	0	0	1	2	1
3	1	0	0	0	0	1	0

As Table S1, but for the feature "number of introns in 5' UTR."
