# Supplementary material for: Genome-Wide Identification of Alternative Splice Forms Down-Regulated by Nonsense-Mediated mRNA Decay in Drosophila
Source: PLoS Genet. 2009 Jun 19;5(6):e1000525. doi: 10.1371/journal.pgen.1000525 (PMC2689934; doi:10.1371/journal.pgen.1000525)
Supplement: Table S3 — GO terms enriched in stringent upf1 set. (0.04 MB PDF) [file pgen.1000525.s025.pdf]

Table S3. GO terms enriched in stringent *upf1* set

GO Term	P-value	Genes
**Biological Process**		
GO:0006412 translation	0.0009	RpL10Ab glo RpL8 RpS15 sqd RpL34b sta
GO:0051231 spindle elongation	0.0031	RpL10Ab RpL8 RpS15 sta
GO:0000022 mitotic spindle elongation	0.0031	RpL10Ab RpL8 RpS15 sta
GO:0044267 cellular protein metabolic process	0.0038	RpL10Ab glo RpL8 RpS15 CG3731 grass sqd RpL34b sta SNF4Agamma CG10107 Tao-1 CaMKII
GO:0019538 protein metabolic process	0.0040	RpL10Ab glo RpL8 RpS15 CG3731 grass sqd RpL34b sta SNF4Agamma CG10107 Tao-1 CaMKII
GO:0007052 mitotic spindle organization	0.0041	CG13900 RpL10Ab RpL8 RpS15 sta
GO:0000226 microtubule cytoskeleton organization	0.0054	CG13900 RpL10Ab RpL8 RpS15 sqd sta
GO:0007051 spindle organization	0.0086	CG13900 RpL10Ab RpL8 RpS15 sta
**Cellular Component**		
GO:0022626 cytosolic ribosome	0.0005	RpL10Ab RpL8 RpS15 RpL34b sta
GO:0030529 ribonucleoprotein complex	0.0006	CG13900 RpL10Ab RpL8 RpS15 sqd RpL34b sta
GO:0033279 ribosomal subunit	0.0009	RpL10Ab RpL8 RpS15 RpL34b sta
GO:0005840 ribosome	0.0010	RpL10Ab RpL8 RpS15 RpL34b sta
GO:0044445 cytosolic part	0.0012	RpL10Ab RpL8 RpS15 RpL34b sta
GO:0033180 proton-transporting V-type ATPase, V1 domain	0.0029	Vha26 VhaSFD
GO:0000221 vacuolar proton-transporting V-type ATPase, V1 domain	0.0029	Vha26 VhaSFD
GO:0022625 cytosolic large ribosomal subunit	0.0045	RpL10Ab RpL8 RpL34b
GO:0015934 large ribosomal subunit	0.0059	RpL10Ab RpL8 RpL34b
GO:0033176 proton-transporting V-type ATPase complex	0.0077	Vha26 VhaSFD
GO:0016471 vacuolar proton-transporting V-type ATPase complex	0.0077	Vha26 VhaSFD
GO:0033178 proton-transporting two-sector ATPase complex, catalytic domain	0.0098	Vha26 VhaSFD
**Molecular Function**		
GO:0003735 structural constituent of ribosome	0.0008	RpL10Ab RpL8 RpS15 RpL34b sta
GO:0005198 structural molecule activity	0.0015	RpL10Ab RpL8 RpS15 RpL34b CG4673 sta
