# Supplementary material for: Genome-Wide Identification of Alternative Splice Forms Down-Regulated by Nonsense-Mediated mRNA Decay in Drosophila
Source: PLoS Genet. 2009 Jun 19;5(6):e1000525. doi: 10.1371/journal.pgen.1000525 (PMC2689934; doi:10.1371/journal.pgen.1000525)
Supplement: Table S4 — GO terms enriched in less stringent upf1 set. (0.03 MB PDF) [file pgen.1000525.s026.pdf]

Table S4. GO terms enriched in less stringent *upf1* set

GO Term	P-value	Genes
Biological Process		
GO:0045807 positive regulation of endocytosis	0.0046	Syx7 Nedd4
Molecular Function		
GO:0016409 palmitoyltransferase activity	0.0046	CPTI Spt-I
GO:0043169 cation binding	0.0056	Actn Fer1HCH Tace ap Nrg Cpn CG10126
GO:0046872 metal ion binding	0.0066	Actn Fer1HCH Tace ap Nrg Cpn CG10126
GO:0043167 ion binding	0.0066	Actn Fer1HCH Tace ap Nrg Cpn CG10126
