# Supplementary material for: Genome-Wide Identification of Alternative Splice Forms Down-Regulated by Nonsense-Mediated mRNA Decay in Drosophila
Source: PLoS Genet. 2009 Jun 19;5(6):e1000525. doi: 10.1371/journal.pgen.1000525 (PMC2689934; doi:10.1371/journal.pgen.1000525)
Supplement: Table S5 — Number of genes successfully deconvolved. The number of genes deconvolved by the analysis. “Deconvolved” indicates that the deconvolution was successful, “too few configurations” indicates the there were too few probe configurations to deconvolve the gene and “inconsistent” indicates that the gene could be deconvolved, but the estimates were inconsistent with the gene model. (0.03 MB PDF) [file pgen.1000525.s027.pdf]

Table S5. Number of genes successfully deconvolved

Experiment	deconvolved	too few configurations	inconsistent	total
upf1, 2 isoforms	1410	137	29	1576
upf1, 3+ isoforms	668	437	19	1124
upf2, 2 isoforms	1433	137	6	1576
upf2, 3+ isoforms	684	437	3	1124

The number of genes deconvolved by the analysis. "Deconvolved" indicates that the deconvolution was successful, "too few configurations" indicates the there were too few probe configurations to deconvolve the gene and "inconsistent" indicates that the gene could be deconvolved, but the estimates were inconsistent with the gene model.
