# Supplementary material for: Genome-Wide Identification of Alternative Splice Forms Down-Regulated by Nonsense-Mediated mRNA Decay in Drosophila
Source: PLoS Genet. 2009 Jun 19;5(6):e1000525. doi: 10.1371/journal.pgen.1000525 (PMC2689934; doi:10.1371/journal.pgen.1000525)
Supplement: Table S6 — upf2 target genes, stringent set. Set of NMD targets for upf2, using the stringent cutoff. (0.04 MB PDF) [file pgen.1000525.s028.pdf]

Table S6. *upf2* target genes, stringent set

gene ID	name	transcript	NMD status
CG33206	l(1)G0168	CG33206-RB	target
		CG33206-RA	nontarget
CG3321		CG3321-RA	target
		CG3321-RB	nontarget
CG3358		CG3358-RA	target
		CG3358-RB	nontarget
CG3629	Dll	CG3629-RB	target
		CG3629-RA	nontarget
CG5215	Zn72D	CG5215-RA	target
		CG5215-RB	nontarget
CG5896	grass	CG5896-RA	target
		CG5896-RB	nontarget
CG6359		CG6359-RA	target
		CG6359-RB	nontarget
CG6454		CG6454-RA	target
		CG6454-RB	nontarget
CG7766		CG7766-RA	target
		CG7766-RB	nontarget
CG8332	RpS15	CG8332-RB	target
		CG8332-RA	nontarget
CG8857	RpS11	CG8857-RB	target
		CG8857-RA	nontarget
CG17077	pnt	CG17077-RB	target
		CG17077-RD	nontarget
		CG17077-RC	possibly absent
CG31332	unc-115	CG31332-RD	target
		CG31332-RB	nontarget
		CG31332-RC	nontarget
		CG31332-RA	possibly absent
CG32149	RhoGAP71E	CG32149-RC	target
		CG32149-RB	nontarget
		CG32149-RA	possibly absent
CG33261	Trl	CG33261-RF	target
		CG33261-RA	nontarget
		CG33261-RB	possibly absent
		CG33261-RC	possibly absent
		CG33261-RD	possibly absent
		CG33261-RE	possibly absent
CG3671	Mvl	CG3671-RB	target
		CG3671-RA	nontarget
		CG3671-RC	possibly absent
CG4452		CG4452-RB	target
		CG4452-RA	nontarget
		CG4452-RC	nontarget
CG6946	glo	CG6946-RC	target
		CG6946-RA	nontarget
		CG6946-RB	possibly absent

Set of NMD targets for *upf2*, using the stringent cutoff.
