# Supplementary material for: Genome-Wide Identification of Alternative Splice Forms Down-Regulated by Nonsense-Mediated mRNA Decay in Drosophila
Source: PLoS Genet. 2009 Jun 19;5(6):e1000525. doi: 10.1371/journal.pgen.1000525 (PMC2689934; doi:10.1371/journal.pgen.1000525)
Supplement: Table S7 — upf1 target genes, less stringent set. (0.05 MB PDF) [file pgen.1000525.s029.pdf]

## Table S7. *upf1* target genes, less stringent set

| gene ID | name | transcript | NMD status |
|---|---|---|---|
| CG10079 | Egfr | CG10079-RB | target |
| | | CG10079-RA | nontarget |
| CG10126 | | CG10126-RA | target |
| | | CG10126-RB | nontarget |
| CG10371 | Plip | CG10371-RA | target |
| | | CG10371-RB | nontarget |
| CG10494 | | CG10494-RB | target |
| | | CG10494-RA | nontarget |
| CG10699 | Lim3 | CG10699-RB | target |
| | | CG10699-RA | nontarget |
| CG1088 | Vha26 | CG1088-RB | target |
| | | CG1088-RA | nontarget |
| CG10901 | osk | CG10901-RA | target |
| | | CG10901-RB | nontarget |
| CG1104 | | CG1104-RA | target |
| | | CG1104-RB | nontarget |
| CG11760 | | CG11760-RA | target |
| | | CG11760-RB | nontarget |
| CG11779 | | CG11779-RA | target |
| | | CG11779-RB | nontarget |
| CG1200 | Aplip1 | CG1200-RB | target |
| | | CG1200-RA | nontarget |
| CG12101 | Hsp60 | CG12101-RA | target |
| | | CG12101-RB | nontarget |
| CG12134 | | CG12134-RB | target |
| | | CG12134-RA | nontarget |
| CG1233 | | CG1233-RA | target |
| | | CG1233-RB | nontarget |
| CG12342 | dgo | CG12342-RA | target |
| | | CG12342-RB | nontarget |
| CG1263 | RpL8 | CG1263-RB | target |
| | | CG1263-RA | nontarget |
| CG12891 | CPTI | CG12891-RB | target |
| | | CG12891-RA | nontarget |
| CG13204 | | CG13204-RB | target |
| | | CG13204-RA | nontarget |
| CG1338 | hydra | CG1338-RA | target |
| | | CG1338-RB | nontarget |
| CG13521 | robo | CG13521-RA | target |
| | | CG13521-RB | nontarget |
| CG1362 | cdc2rk | CG1362-RA | target |
| | | CG1362-RB | nontarget |
| CG13900 | | CG13900-RA | target |
| | | CG13900-RB | nontarget |
| CG13923 | | CG12022-RA | target |
| | | CG13923-RA | nontarget |
| CG14444 | APC7 | CG14444-RB | target |
| | | CG14444-RA | nontarget |
| CG1462 | Aph-4 | CG1462-RB | target |
| | | CG1462-RA | nontarget |
| CG1486 | | CG1486-RB | target |
| | | CG1486-RA | nontarget |
| CG1488 | Cyp311a1 | CG1488-RB | target |
| | | CG1488-RA | nontarget |
| CG15117 | | CG15117-RA | target |
| | | CG15117-RB | nontarget |
| CG15626 | | CG15626-RB | target |
| | | CG15626-RA | nontarget |
| CG15881 | | CG15881-RB | target |
| | | CG15881-RA | nontarget |
| CG1648 | | CG1648-RA | target |

Continued on next page

Table S7 – continued from previous page

| gene ID | name | transcript | NMD status |
|---|---|---|---|
| | | CG1648-RB | nontarget |
| CG16718 | | CG16718-RB | target |
| | | CG16718-RA | nontarget |
| CG17035 | GXIVsPLA2 | CG17035-RB | target |
| | | CG17035-RA | nontarget |
| CG18009 | Trf2 | CG18009-RA | target |
| | | CG18009-RD | nontarget |
| CG1866 | Moca-cyp | CG1866-RB | target |
| | | CG1866-RA | nontarget |
| CG1902 | | CG1902-RC | target |
| | | CG1902-RA | nontarget |
| CG2152 | Pcmt | CG2152-RB | target |
| | | CG2152-RA | nontarget |
| CG2177 | | CG2177-RB | target |
| | | CG2177-RA | nontarget |
| CG2179 | Xe7 | CG2179-RA | target |
| | | CG2179-RB | nontarget |
| CG30015 | | CG30015-RA | target |
| | | CG30015-RB | nontarget |
| CG31751 | | CG31751-RB | target |
| | | CG31751-RA | nontarget |
| CG3182 | sei | CG3182-RB | target |
| | | CG3182-RA | nontarget |
| CG31961 | | CG31961-RA | target |
| | | CG31961-RB | nontarget |
| CG32140 | nuf | CG32140-RB | target |
| | | CG32140-RA | nontarget |
| CG3217 | CkIIalpha-i3 | CG3217-RB | target |
| | | CG3217-RA | nontarget |
| CG3241 | msl-2 | CG3241-RB | target |
| | | CG3241-RA | nontarget |
| CG32647 | | CG32647-RA | target |
| | | CG32647-RB | nontarget |
| CG33085 | | CG33085-RD | target |
| | | CG33085-RA | nontarget |
| CG33206 | l(1)G0168 | CG33206-RB | target |
| | | CG33206-RA | nontarget |
| CG3358 | | CG3358-RA | target |
| | | CG3358-RB | nontarget |
| CG3530 | | CG3530-RB | target |
| | | CG3530-RA | nontarget |
| CG3629 | Dll | CG3629-RB | target |
| | | CG3629-RA | nontarget |
| CG3731 | | CG3731-RA | target |
| | | CG3731-RB | nontarget |
| CG3861 | kdn | CG3861-RA | target |
| | | CG3861-RB | nontarget |
| CG4059 | ftz-f1 | CG4059-RA | target |
| | | CG4059-RB | nontarget |
| CG4070 | Tis11 | CG4070-RB | target |
| | | CG4070-RA | nontarget |
| CG4143 | mbf1 | CG4143-RB | target |
| | | CG4143-RA | nontarget |
| CG4247 | mRpS10 | CG4247-RB | target |
| | | CG4247-RA | nontarget |
| CG4590 | inx2 | CG4590-RB | target |
| | | CG4590-RA | nontarget |
| CG4609 | fax | CG4609-RA | target |
| | | CG4609-RB | nontarget |
| CG4673 | | CG4673-RB | target |
| | | CG4673-RA | nontarget |

Continued on next page

Table S7 – continued from previous page

| gene ID | name | transcript | NMD status |
|---|---|---|---|
| CG4712 | | CG4712-RB | target |
| | | CG4712-RA | nontarget |
| CG4795 | Cpn | CG4795-RB | target |
| | | CG4795-RA | nontarget |
| CG5081 | Syx7 | CG5081-RA | target |
| | | CG5081-RB | nontarget |
| CG5215 | Zn72D | CG5215-RA | target |
| | | CG5215-RB | nontarget |
| CG5326 | | CG5326-RB | target |
| | | CG5326-RA | nontarget |
| CG5394 | Aats-glupro | CG5394-RA | target |
| | | CG5394-RB | nontarget |
| CG5613 | | CG5613-RB | target |
| | | CG5613-RA | nontarget |
| CG5625 | | CG5625-RA | target |
| | | CG5625-RB | nontarget |
| CG5729 | Dgp-1 | CG5729-RB | target |
| | | CG5729-RA | nontarget |
| CG5785 | thr | CG5785-RB | target |
| | | CG5785-RA | nontarget |
| CG5854 | | CG5854-RA | target |
| | | CG5854-RB | nontarget |
| CG5896 | grass | CG5896-RA | target |
| | | CG5896-RB | nontarget |
| CG6023 | | CG6023-RB | target |
| | | CG6023-RA | nontarget |
| CG6084 | | CG6084-RB | target |
| | | CG6084-RA | nontarget |
| CG6090 | RpL34a | CG6090-RB | target |
| | | CG6090-RA | nontarget |
| CG6297 | JIL-1 | CG6297-RA | target |
| | | CG6297-RB | nontarget |
| CG6315 | fl(2)d | CG6315-RB | target |
| | | CG6315-RA | nontarget |
| CG6454 | | CG6454-RA | target |
| | | CG6454-RB | nontarget |
| CG6608 | | CG6608-RB | target |
| | | CG6608-RA | nontarget |
| CG6726 | | CG6726-RA | target |
| | | CG6726-RB | nontarget |
| CG6767 | | CG6767-RA | target |
| | | CG6767-RB | nontarget |
| CG6798 | nAcRbeta-96A | CG6798-RA | target |
| | | CG6798-RB | nontarget |
| CG6829 | Ark | CG6829-RB | target |
| | | CG6829-RA | nontarget |
| CG6891 | | CG6891-RB | target |
| | | CG6891-RA | nontarget |
| CG7070 | PyK | CG7070-RB | target |
| | | CG7070-RA | nontarget |
| CG7263 | | CG7263-RA | target |
| | | CG7263-RB | nontarget |
| CG7334 | Sug | CG7334-RA | target |
| | | CG7334-RB | nontarget |
| CG7540 | M6 | CG7540-RA | target |
| | | CG7540-RB | nontarget |
| CG7725 | rogdi | CG7725-RA | target |
| | | CG7725-RB | nontarget |
| CG7908 | Tace | CG7908-RB | target |
| | | CG7908-RA | nontarget |
| CG8318 | Nf1 | CG8318-RB | target |

Continued on next page

Table S7 – continued from previous page

| gene ID | name | transcript | NMD status |
|---|---|---|---|
| | | CG8318-RC | nontarget |
| CG8327 | SpdS | CG8327-RB | target |
| | | CG8327-RA | nontarget |
| CG8332 | RpS15 | CG8332-RB | target |
| | | CG8332-RA | nontarget |
| CG8376 | ap | CG8376-RB | target |
| | | CG8376-RA | nontarget |
| CG8430 | Got1 | CG8430-RA | target |
| | | CG8430-RB | nontarget |
| CG8486 | | CG8486-RB | target |
| | | CG8486-RA | nontarget |
| CG8495 | RpS29 | CG8495-RA | target |
| | | CG8495-RC | nontarget |
| CG8557 | | CG8557-RA | target |
| | | CG8557-RB | nontarget |
| CG8765 | | CG8765-RA | target |
| | | CG8765-RB | nontarget |
| CG8783 | | CG8783-RA | target |
| | | CG8783-RB | nontarget |
| CG8811 | muskelin | CG8811-RA | target |
| | | CG8811-RB | nontarget |
| CG8944 | | CG8944-RB | target |
| | | CG8944-RA | nontarget |
| CG8956 | Ahcy89E | CG8956-RC | target |
| | | CG8956-RD | nontarget |
| CG9195 | Scamp | CG9195-RA | target |
| | | CG9195-RB | nontarget |
| CG9248 | | CG9248-RB | target |
| | | CG9248-RA | nontarget |
| CG9256 | Nhe2 | CG9256-RA | target |
| | | CG9256-RB | nontarget |
| CG9354 | RpL34b | CG9354-RA | target |
| | | CG9354-RB | nontarget |
| CG9413 | | CG9413-RA | target |
| | | CG9413-RB | nontarget |
| CG9415 | Xbp1 | CG9415-RA | target |
| | | CG9415-RB | nontarget |
| CG9425 | | CG9425-RB | target |
| | | CG9425-RA | nontarget |
| CG9611 | | CG9611-RB | target |
| | | CG9611-RA | nontarget |
| CR32885 | pgc | CR32885-RB | target |
| | | CR32885-RA | nontarget |
| CG10023 | Fak56D | CG10023-RA | target |
| | | CG10023-RB | nontarget |
| | | CG10023-RC | possibly absent |
| CG10107 | | CG10107-RA | target |
| | | CG10107-RC | target |
| | | CG10107-RB | nontarget |
| CG10121 | SP1173 | CG10121-RB | target |
| | | CG10121-RA | nontarget |
| | | CG10121-RC | possibly absent |
| | | CG10121-RD | possibly absent |
| CG10772 | Fur1 | CG10772-RC | target |
| | | CG10772-RD | nontarget |
| | | CG10772-RA | possibly absent |
| | | CG10772-RB | possibly absent |
| | | CG10772-RE | possibly absent |
| | | CG10772-RF | possibly absent |
| CG10868 | orb | CG10868-RC | target |
| | | CG10868-RA | nontarget |

Continued on next page

Table S7 – continued from previous page

| gene ID | name | transcript | NMD status |
|---|---|---|---|
| | | CG10868-RB | nontarget |
| CG10948 | | CG10948-RB | target |
| | | CG10948-RC | nontarget |
| | | CG10948-RA | possibly absent |
| CG11081 | plexA | CG11081-RA | target |
| | | CG11081-RD | nontarget |
| | | CG11081-RB | possibly absent |
| | | CG11081-RC | possibly absent |
| CG11100 | Mes2 | CG11100-RC | target |
| | | CG11100-RA | nontarget |
| | | CG11100-RB | nontarget |
| CG11163 | | CG11163-RA | target |
| | | CG11163-RB | target |
| | | CG11163-RD | target |
| | | CG11163-RC | nontarget |
| CG11537 | | CG11537-RC | target |
| | | CG11537-RA | nontarget |
| | | CG11537-RB | possibly absent |
| CG12085 | pUf68 | CG12085-RD | target |
| | | CG12085-RB | nontarget |
| | | CG12085-RA | possibly absent |
| | | CG12085-RC | possibly absent |
| CG1213 | | CG1213-RA | target |
| | | CG1213-RB | nontarget |
| | | CG1213-RC | nontarget |
| CG12746 | | CG12746-RB | target |
| | | CG12746-RD | nontarget |
| | | CG12746-RA | possibly absent |
| | | CG12746-RC | possibly absent |
| CG14217 | Tao-1 | CG14217-RB | target |
| | | CG14217-RA | nontarget |
| | | CG14217-RD | possibly absent |
| | | CG14217-RE | possibly absent |
| CG14414 | | CG14414-RA | target |
| | | CG14414-RB | nontarget |
| | | CG14414-RC | possibly absent |
| CG14823 | | CG14823-RC | target |
| | | CG14823-RA | nontarget |
| | | CG14823-RB | nontarget |
| | | CG14823-RD | nontarget |
| CG14938 | crol | CG14938-RB | target |
| | | CG14938-RA | nontarget |
| | | CG14938-RD | nontarget |
| | | CG14938-RC | possibly absent |
| CG1623 | | CG1623-RA | target |
| | | CG1623-RC | target |
| | | CG1623-RE | nontarget |
| CG1634 | Nrg | CG1634-RC | target |
| | | CG1634-RB | nontarget |
| | | CG1634-RA | possibly absent |
| CG16833 | | CG16833-RA | target |
| | | CG16833-RB | nontarget |
| | | CG16833-RC | possibly absent |
| CG16901 | sqd | CG16901-RD | target |
| | | CG16901-RB | nontarget |
| | | CG16901-RA | possibly absent |
| | | CG16901-RC | possibly absent |
| CG16952 | | CG16952-RB | target |
| | | CG16952-RA | nontarget |
| | | CG16952-RC | possibly absent |
| CG16973 | msn | CG16973-RA | target |

Continued on next page

Table S7 – continued from previous page

| gene ID | name | transcript | NMD status |
|---|---|---|---|
| | | CG16973-RD | target |
| | | CG16973-RB | nontarget |
| | | CG16973-RC | possibly absent |
| | | CG16973-RE | possibly absent |
| CG17332 | VhaSFD | CG17332-RA | target |
| | | CG17332-RB | nontarget |
| | | CG17332-RD | possibly absent |
| CG17834 | | CG17834-RB | target |
| | | CG17834-RA | nontarget |
| | | CG17834-RC | possibly absent |
| | | CG17834-RD | possibly absent |
| CG18069 | CaMKII | CG18069-RB | target |
| | | CG18069-RC | nontarget |
| | | CG18069-RA | possibly absent |
| CG18769 | | CG18769-RF | target |
| | | CG18769-RB | nontarget |
| | | CG18769-RA | possibly absent |
| | | CG18769-RC | possibly absent |
| | | CG18769-RD | possibly absent |
| | | CG18769-RE | possibly absent |
| CG1877 | lin19 | CG1877-RA | target |
| | | CG1877-RD | nontarget |
| | | CG1877-RB | possibly absent |
| | | CG1877-RC | possibly absent |
| CG2216 | Fer1HCH | CG2216-RA | target |
| | | CG2216-RB | target |
| | | CG2216-RC | target |
| | | CG2216-RD | nontarget |
| | | CG2216-RE | possibly absent |
| CG2225 | | CG2225-RC | target |
| | | CG2225-RD | nontarget |
| | | CG2225-RA | possibly absent |
| | | CG2225-RB | possibly absent |
| | | CG2225-RE | possibly absent |
| CG2304 | Trc8 | CG2304-RA | target |
| | | CG2304-RD | target |
| | | CG2304-RB | nontarget |
| | | CG2304-RC | possibly absent |
| CG31045 | Mhcl | CG31045-RA | target |
| | | CG31045-RD | nontarget |
| | | CG31045-RB | possibly absent |
| | | CG31045-RC | possibly absent |
| | | CG31045-RE | possibly absent |
| CG31237 | Rpb4 | CG31318-RA | target |
| | | CG31318-RB | target |
| | | CG31237-RA | nontarget |
| CG31305 | | CG31305-RA | target |
| | | CG31305-RG | nontarget |
| | | CG31305-RI | nontarget |
| | | CG31305-RB | possibly absent |
| | | CG31305-RD | possibly absent |
| | | CG31305-RF | possibly absent |
| CG31332 | unc-115 | CG31332-RA | target |
| | | CG31332-RB | target |
| | | CG31332-RD | target |
| | | CG31332-RC | nontarget |
| CG31536 | Cdep | CG31536-RA | target |
| | | CG31536-RC | target |
| | | CG31536-RB | nontarget |
| CG31764 | vir-1 | CG31764-RA | target |
| | | CG31764-RB | nontarget |

Continued on next page

Table S7 – continued from previous page

| gene ID | name | transcript | NMD status |
|---|---|---|---|
| | | CG31764-RC | nontarget |
| CG32018 | Zyx102EF | CG32018-RB | target |
| | | CG32018-RC | target |
| | | CG32018-RE | nontarget |
| | | CG32018-RA | possibly absent |
| | | CG32018-RD | possibly absent |
| | | CG32018-RF | possibly absent |
| | | CG32018-RG | possibly absent |
| CG32423 | shep | CG32423-RD | target |
| | | CG32423-RB | nontarget |
| | | CG32423-RA | possibly absent |
| | | CG32423-RC | possibly absent |
| CG32508 | shaking B | CG32508-RA | target |
| | | CG32508-RB | nontarget |
| | | CG32508-RC | nontarget |
| CG32538 | gfA | CG32538-RA | target |
| | | CG32538-RB | nontarget |
| | | CG32538-RC | nontarget |
| CG32858 | sn | CG32858-RB | target |
| | | CG32858-RA | nontarget |
| | | CG32858-RC | possibly absent |
| CG33054 | | CG33056-RB | target |
| | | CG33056-RD | target |
| | | CG33054-RA | nontarget |
| | | CG33056-RC | nontarget |
| | | CG33054-RB | possibly absent |
| | | CG33056-RA | possibly absent |
| | | CG33056-RE | possibly absent |
| CG33129 | | CG33129-RE | target |
| | | CG33129-RA | nontarget |
| | | CG33129-RB | nontarget |
| | | CG33129-RC | possibly absent |
| CG33184 | | CG33184-RC | target |
| | | CG33184-RA | nontarget |
| | | CG33184-RB | possibly absent |
| CG33261 | Trl | CG33261-RF | target |
| | | CG33261-RA | nontarget |
| | | CG33261-RC | nontarget |
| | | CG33261-RD | nontarget |
| | | CG33261-RE | nontarget |
| | | CG33261-RB | possibly absent |
| CG3638 | | CG3638-RC | target |
| | | CG3638-RA | nontarget |
| | | CG3638-RB | nontarget |
| | | CG3638-RD | nontarget |
| CG3948 | zetaCOP | CG3948-RC | target |
| | | CG3948-RA | nontarget |
| | | CG3948-RB | nontarget |
| CG4016 | Spt-I | CG4016-RB | target |
| | | CG4016-RA | nontarget |
| | | CG4016-RC | possibly absent |
| CG4239 | | CG4239-RA | target |
| | | CG4239-RB | target |
| | | CG4239-RC | nontarget |
| CG4376 | Actn | CG4376-RB | target |
| | | CG4376-RA | nontarget |
| | | CG4376-RC | possibly absent |
| CG4389 | | CG4389-RA | target |
| | | CG4389-RB | nontarget |
| | | CG4389-RC | possibly absent |
| CG4452 | | CG4452-RB | target |

Continued on next page

Table S7 – continued from previous page

| gene ID | name | transcript | NMD status |
|---|---|---|---|
| | | CG4452-RA | nontarget |
| | | CG4452-RC | possibly absent |
| CG4816 | qkr54B | CG4816-RA | target |
| | | CG4816-RB | nontarget |
| | | CG4816-RC | possibly absent |
| CG4921 | Rab4 | CG4921-RC | target |
| | | CG4921-RA | nontarget |
| | | CG4921-RB | possibly absent |
| CG5288 | | CG5288-RB | target |
| | | CG5288-RC | target |
| | | CG5288-RA | nontarget |
| CG5461 | bun | CG5461-RA | target |
| | | CG5461-RB | nontarget |
| | | CG5461-RC | possibly absent |
| CG5486 | Ubp64E | CG5486-RB | target |
| | | CG5486-RA | nontarget |
| | | CG5486-RC | possibly absent |
| CG6854 | | CG6854-RA | target |
| | | CG6854-RB | nontarget |
| | | CG6854-RC | possibly absent |
| CG6946 | glo | CG6946-RC | target |
| | | CG6946-RA | nontarget |
| | | CG6946-RB | possibly absent |
| CG7176 | Idh | CG7176-RF | target |
| | | CG7176-RD | nontarget |
| | | CG7176-RA | possibly absent |
| | | CG7176-RB | possibly absent |
| | | CG7176-RC | possibly absent |
| | | CG7176-RE | possibly absent |
| | | CG7176-RG | possibly absent |
| CG7283 | RpL10Ab | CG7283-RB | target |
| | | CG7283-RA | nontarget |
| | | CG7283-RC | possibly absent |
| CG7555 | Nedd4 | CG7555-RD | target |
| | | CG7555-RB | nontarget |
| | | CG7555-RA | possibly absent |
| | | CG7555-RC | possibly absent |
| CG7926 | Axn | CG7926-RA | target |
| | | CG7926-RB | nontarget |
| | | CG7926-RC | possibly absent |
| CG8121 | | CG8121-RA | target |
| | | CG8121-RC | target |
| | | CG8121-RB | nontarget |
| CG8127 | Eip75B | CG8127-RB | target |
| | | CG8127-RA | nontarget |
| | | CG8127-RC | possibly absent |
| | | CG8127-RD | possibly absent |
| CG8178 | Nach | CG8178-RB | target |
| | | CG8178-RC | nontarget |
| | | CG8178-RA | possibly absent |
| CG8363 | Paps | CG8363-RE | target |
| | | CG8363-RD | nontarget |
| | | CG8363-RA | possibly absent |
| | | CG8363-RB | possibly absent |
| | | CG8363-RC | possibly absent |
| CG8440 | Lis-1 | CG8440-RC | target |
| | | CG8440-RD | target |
| | | CG8440-RE | nontarget |
| | | CG8440-RA | possibly absent |
| | | CG8440-RB | possibly absent |
| | | CG8440-RF | possibly absent |

Continued on next page

Table S7 – continued from previous page

| gene ID | name | transcript | NMD status |
|---|---|---|---|
| CG8465 | l(1)G0222 | CG8465-RA | target |
| | | CG8465-RC | nontarget |
| | | CG8465-RB | possibly absent |
| CG8663 | nrv3 | CG8663-RB | target |
| | | CG8663-RD | nontarget |
| | | CG8663-RA | possibly absent |
| | | CG8663-RC | possibly absent |
| CG8732 | l(2)44DEa | CG8732-RA | target |
| | | CG8732-RB | nontarget |
| | | CG8732-RC | possibly absent |
| CG9277 | betaTub56D | CG9277-RB | target |
| | | CG9277-RA | nontarget |
| | | CG9277-RC | possibly absent |
| | | CG9277-RD | possibly absent |
| CG9381 | mura | CG9381-RA | target |
| | | CG9381-RB | nontarget |
| | | CG9381-RC | possibly absent |
| CG9755 | pum | CG9755-RC | target |
| | | CG9755-RE | target |
| | | CG9755-RA | nontarget |
| | | CG9755-RB | possibly absent |
| | | CG9755-RD | possibly absent |
| CG9772 | | CG9772-RA | target |
| | | CG9772-RC | nontarget |
| | | CG9772-RB | possibly absent |
