# Supplementary material for: Genome-Wide Identification of Alternative Splice Forms Down-Regulated by Nonsense-Mediated mRNA Decay in Drosophila
Source: PLoS Genet. 2009 Jun 19;5(6):e1000525. doi: 10.1371/journal.pgen.1000525 (PMC2689934; doi:10.1371/journal.pgen.1000525)
Supplement: Table S8 — upf2 target genes, less stringent set. (0.04 MB PDF) [file pgen.1000525.s030.pdf]

Table S8. *upf2* target genes, less stringent set

gene ID	name	transcript	NMD status
CG1104		CG1104-RA	target
		CG1104-RB	nontarget
CG1109		CG1109-RA	target
		CG1109-RB	nontarget
CG11328	Nhe3	CG11328-RB	target
		CG11328-RA	nontarget
CG11961		CG11961-RB	target
		CG11961-RA	nontarget
CG12134		CG12134-RB	target
		CG12134-RA	nontarget
CG12864	Su(var)2-HP2	CG12864-RA	target
		CG12864-RB	nontarget
CG13900		CG13900-RA	target
		CG13900-RB	nontarget
CG13923		CG12022-RA	target
		CG13923-RA	nontarget
CG1546	PH4alphaSG2	CG1546-RB	target
		CG1546-RA	nontarget
CG17035	GXIVsPLA2	CG17035-RB	target
		CG17035-RA	nontarget
CG17369	Vha55	CG17369-RB	target
		CG17369-RA	nontarget
CG1771	mew	CG1771-RB	target
		CG1771-RA	nontarget
CG1866	Moca-cyp	CG1866-RB	target
		CG1866-RA	nontarget
CG2182		CG2182-RA	target
		CG2182-RB	nontarget
CG2534	cno	CG2534-RA	target
		CG2534-RB	nontarget
CG31619		CG31619-RA	target
		CG31619-RB	nontarget
CG32527		CG32527-RB	target
		CG32527-RA	nontarget
CG33103	Ppn	CG33103-RA	target
		CG33103-RB	nontarget
CG33206	l(1)G0168	CG33206-RB	target
		CG33206-RA	nontarget
CG3321		CG3321-RA	target
		CG3321-RB	nontarget
CG3358		CG3358-RA	target
		CG3358-RB	nontarget
CG3629	Dll	CG3629-RB	target
		CG3629-RA	nontarget
CG3941	pita	CG3941-RA	target
		CG3941-RB	nontarget
CG4062	Aats-val	CG4062-RA	target
		CG4062-RB	nontarget
CG4482	mol	CG4482-RA	target
		CG4482-RB	nontarget
CG4879	RecQ5	CG4879-RB	target
		CG4879-RA	nontarget
CG5081	Syx7	CG5081-RA	target
		CG5081-RB	nontarget
CG5215	Zn72D	CG5215-RA	target
		CG5215-RB	nontarget
CG5729	Dgp-1	CG5729-RB	target
		CG5729-RA	nontarget
CG5854		CG5854-RB	target
		CG5854-RA	nontarget
CG5896	grass	CG5896-RA	target

Continued on next page

Table S8 – continued from previous page

gene ID	name	transcript	NMD status
		CG5896-RB	nontarget
CG6359		CG6359-RA	target
		CG6359-RB	nontarget
CG6454		CG6454-RA	target
		CG6454-RB	nontarget
CG7602	DNApol-iota	CG7602-RB	target
		CG7602-RA	nontarget
CG7766		CG7766-RA	target
		CG7766-RB	nontarget
CG8332	RpS15	CG8332-RB	target
		CG8332-RA	nontarget
CG8478		CG8478-RB	target
		CG8478-RA	nontarget
CG8811	muskelin	CG8811-RA	target
		CG8811-RB	nontarget
CG8857	RpS11	CG8857-RB	target
		CG8857-RA	nontarget
CG8983	ERp60	CG8983-RA	target
		CG8983-RB	nontarget
CG9153		CG9153-RA	target
		CG9153-RB	nontarget
CG9354	RpL34b	CG9354-RA	target
		CG9354-RB	nontarget
CG10107		CG10107-RA	target
		CG10107-RB	nontarget
		CG10107-RC	possibly absent
CG10772	Fur1	CG10772-RE	target
		CG10772-RF	nontarget
		CG10772-RA	possibly absent
		CG10772-RB	possibly absent
		CG10772-RC	possibly absent
		CG10772-RD	possibly absent
CG14025	Bsg25D	CG14025-RB	target
		CG14025-RC	nontarget
		CG14025-RA	possibly absent
CG14414		CG14414-RA	target
		CG14414-RB	nontarget
		CG14414-RC	possibly absent
CG1447	Ptx1	CG1447-RC	target
		CG1447-RB	nontarget
		CG1447-RA	possibly absent
CG14792	sta	CG14792-RA	target
		CG14792-RB	nontarget
		CG14792-RD	possibly absent
CG14823		CG14823-RC	target
		CG14823-RB	nontarget
		CG14823-RA	possibly absent
		CG14823-RD	possibly absent
CG15086		CG15086-RD	target
		CG15086-RA	nontarget
		CG15086-RB	possibly absent
		CG15086-RC	possibly absent
CG1651	Ank	CG1651-RC	target
		CG1651-RB	nontarget
		CG1651-RA	possibly absent
		CG1651-RD	possibly absent
CG17077	pnt	CG17077-RB	target
		CG17077-RC	nontarget
		CG17077-RD	nontarget
CG17299	SNF4Agamma	CG17299-RA	target
		CG17299-RE	target

Continued on next page

Table S8 – continued from previous page

gene ID	name	transcript	NMD status
		CG17299-RG	target
		CG17299-RB	nontarget
		CG17299-RD	nontarget
		CG17299-RC	possibly absent
		CG17299-RF	possibly absent
		CG17299-RH	possibly absent
CG17952	LBR	CG17952-RC	target
		CG17952-RB	nontarget
		CG17952-RA	possibly absent
CG18660	Nckx30C	CG18660-RC	target
		CG18660-RB	nontarget
		CG18660-RA	possibly absent
CG2040	hig	CG2040-RC	target
		CG2040-RB	nontarget
		CG2040-RA	possibly absent
CG2098	ferrochelatase	CG2098-RB	target
		CG2098-RA	nontarget
		CG2098-RC	possibly absent
CG2168	RpS3A	CG2168-RA	target
		CG2168-RD	nontarget
		CG2168-RB	possibly absent
CG31137	twin	CG31137-RB	target
		CG31137-RF	nontarget
		CG31137-RA	possibly absent
		CG31137-RC	possibly absent
		CG31137-RD	possibly absent
		CG31137-RE	possibly absent
CG31363	Jupiter	CG31363-RD	target
		CG31363-RC	nontarget
		CG31363-RE	nontarget
		CG31363-RA	possibly absent
		CG31363-RB	possibly absent
		CG31363-RH	possibly absent
CG32103		CG32103-RB	target
		CG32103-RA	nontarget
		CG32103-RC	nontarget
CG32149	RhoGAP71E	CG32149-RC	target
		CG32149-RB	nontarget
		CG32149-RA	possibly absent
CG32858	sn	CG32858-RA	target
		CG32858-RB	nontarget
		CG32858-RC	possibly absent
CG33045		CG33045-RE	target
		CG33045-RG	nontarget
		CG33045-RA	possibly absent
		CG33045-RC	possibly absent
		CG33045-RD	possibly absent
		CG33045-RF	possibly absent
CG33129		CG33129-RE	target
		CG33129-RA	nontarget
		CG33129-RB	nontarget
		CG33129-RC	nontarget
CG33261	Trl	CG33261-RF	target
		CG33261-RA	nontarget
		CG33261-RB	possibly absent
		CG33261-RC	possibly absent
		CG33261-RD	possibly absent
		CG33261-RE	possibly absent
CG3413	wdp	CG3413-RB	target
		CG3413-RD	nontarget
		CG3413-RA	possibly absent

Continued on next page

Table S8 – continued from previous page

gene ID	name	transcript	NMD status
		CG3413-RC	possibly absent
CG3671	Mvl	CG3671-RB	target
		CG3671-RC	target
		CG3671-RA	nontarget
CG3777		CG3777-RC	target
		CG3777-RA	nontarget
		CG3777-RB	possibly absent
CG4376	Actn	CG4376-RB	target
		CG4376-RA	nontarget
		CG4376-RC	possibly absent
CG4452		CG4452-RB	target
		CG4452-RA	nontarget
		CG4452-RC	nontarget
CG4898	Tm1	CG4898-RA	target
		CG4898-RH	target
		CG4898-RB	nontarget
		CG4898-RC	possibly absent
		CG4898-RD	possibly absent
		CG4898-RE	possibly absent
		CG4898-RF	possibly absent
		CG4898-RI	possibly absent
		CG4898-RJ	possibly absent
		CG4898-RK	possibly absent
		CG4898-RL	possibly absent
CG6143	Pep	CG6143-RA	target
		CG6143-RB	target
		CG6143-RC	nontarget
CG6391	Aps	CG6391-RB	target
		CG6391-RA	nontarget
		CG6391-RC	possibly absent
CG6835	GS	CG6835-RC	target
		CG6835-RD	nontarget
		CG6835-RA	possibly absent
CG6946	glo	CG6946-RC	target
		CG6946-RA	nontarget
		CG6946-RB	possibly absent
CG7283	RpL10Ab	CG7283-RB	target
		CG7283-RA	nontarget
		CG7283-RC	possibly absent
CG7852		CG7852-RC	target
		CG7852-RA	nontarget
		CG7852-RB	possibly absent
CG8291		CG8291-RA	target
		CG8291-RB	nontarget
		CG8291-RC	possibly absent
CG8874	Fps85D	CG8874-RB	target
		CG8874-RD	nontarget
		CG8874-RA	possibly absent
		CG8874-RC	possibly absent
CG8920		CG8920-RA	target
		CG8920-RB	nontarget
		CG8920-RC	nontarget
