# Supplementary material for: Genome-Wide Identification of Alternative Splice Forms Down-Regulated by Nonsense-Mediated mRNA Decay in Drosophila
Source: PLoS Genet. 2009 Jun 19;5(6):e1000525. doi: 10.1371/journal.pgen.1000525 (PMC2689934; doi:10.1371/journal.pgen.1000525)
Supplement: Table S9 — Deconvolution results for the stringent set of upf1 affected genes. (0.04 MB PDF) [file pgen.1000525.s031.pdf]

Table S9. Deconvolution results for the stringent set of *upf1* affected genes

Gene	Transcript	alpha	beta	Call
CG1088	CG1088-RA	0.99	0.65	Unchanged
	CG1088-RB	1.85	0.35	Up
CG1263	CG1263-RA	1.09	1.00	Unchanged
	CG1263-RB	2.21	0.00	Up
CG12891	CG12891-RA	1.07	0.94	Unchanged
	CG12891-RB	1.86	0.06	Up
CG13521	CG13521-RA	1.10	1.00	Up
	CG13521-RB	0.67	0.00	Slightly down
CG13900	CG13900-RA	3.13	0.46	Up
	CG13900-RB	1.03	0.54	Unchanged
CG1753	CG1753-RA	1.21	1.00	Unchanged
	CG1753-RB	2.49	0.00	Up
CG18009	CG18009-RA	2.34	0.00	Up
	CG18009-RD	0.97	1.00	Unchanged
CG1902	CG1902-RA	0.71	0.67	Slightly down
	CG1902-RC	1.49	0.33	Up
CG2152	CG2152-RA	0.75	0.97	Slightly down
	CG2152-RB	1.38	0.03	Up
CG33206	CG33206-RA	0.89	0.78	Unchanged
	CG33206-RB	7.59	0.22	Up
CG3358	CG3358-RA	3.48	0.15	Up
	CG3358-RB	0.87	0.85	Unchanged
CG3629	CG3629-RA	0.92	1.00	Unchanged
	CG3629-RB	12.96	0.00	Up
CG3731	CG3731-RA	1.50	0.13	Up
	CG3731-RB	0.87	0.87	Unchanged
CG4059	CG4059-RA	5.15	0.04	Up
	CG4059-RB	0.74	0.96	Slightly down
CG4673	CG4673-RA	0.90	0.56	Unchanged
	CG4673-RB	1.81	0.44	Up
CG5215	CG5215-RA	4.17	0.32	Up
	CG5215-RB	0.98	0.68	Unchanged
CG5896	CG5896-RA	3.47	0.03	Up
	CG5896-RB	0.80	0.97	Slightly down
CG6084	CG6084-RA	0.54	0.56	Slightly down
	CG6084-RB	1.29	0.44	Up
CG6315	CG6315-RA	1.01	0.75	Unchanged
	CG6315-RB	2.71	0.25	Up
CG6454	CG6454-RA	7.77	0.01	Up
	CG6454-RB	1.05	0.99	Unchanged
CG7540	CG7540-RA	1.27	0.70	Up
	CG7540-RB	0.66	0.30	Slightly down
CG8332	CG8332-RA	0.59	1.00	Slightly down
	CG8332-RB	4.37	0.00	Up
CG9248	CG9248-RA	0.61	0.00	Slightly down
	CG9248-RB	1.22	1.00	Up
CG9354	CG9354-RA	3.84	0.00	Up
	CG9354-RB	0.66	1.00	Slightly down
CG9413	CG9413-RA	1.15	0.95	Up
	CG9413-RB	0.59	0.05	Slightly down
CG10107	CG10107-RA	2.75	0.27	Up
	CG10107-RB	0.80	0.73	Unchanged
	CG10107-RC	1.87	0.00	Possibly absent
CG10948	CG10948-RA	0.97	0.00	Possibly absent
	CG10948-RB	2.94	0.15	Up
	CG10948-RC	1.18	0.85	Unchanged
CG14217	CG14217-RA	0.83	0.00	Unchanged
	CG14217-RB	2.74	0.00	Up
	CG14217-RD	0.82	0.00	Possibly absent
	CG14217-RE	1.05	1.00	Possibly absent
CG14792	CG14792-RA	1.04	0.00	Unchanged

Continued on next page

Table S9 – continued from previous page

Gene	Transcript	alpha	beta	Call
	CG14792-RB	4.19	0.00	Up
	CG14792-RD	1.55	1.00	Possibly absent
CG1623	CG1623-RA	1.56	0.00	Possibly absent
	CG1623-RC	2.38	0.00	Up
	CG1623-RE	0.96	1.00	Unchanged
CG16901	CG16901-RA	1.03	0.70	Possibly absent
	CG16901-RB	0.96	0.18	Unchanged
	CG16901-RC	0.79	0.12	Possibly absent
	CG16901-RD	2.45	0.00	Up
CG17299	CG17299-RA	1.47	0.01	Possibly absent
	CG17299-RB	1.12	0.00	Possibly absent
	CG17299-RC	1.27	0.20	Possibly absent
	CG17299-RD	1.13	0.00	Possibly absent
	CG17299-RE	0.96	0.00	Possibly absent
	CG17299-RF	0.83	0.07	Unchanged
	CG17299-RG	1.67	0.35	Up
	CG17299-RH	0.83	0.36	Possibly absent
CG17332	CG17332-RA	1.39	0.68	Up
	CG17332-RB	0.78	0.00	Slightly down
	CG17332-RD	1.09	0.32	Possibly absent
CG18069	CG18069-RA	0.96	0.00	Possibly absent
	CG18069-RB	1.29	1.00	Up
	CG18069-RC	0.93	0.00	Unchanged
CG31237	CG31237-RA	0.86	1.00	Unchanged
	CG31318-RA	1.85	0.00	Up
	CG31318-RB	1.34	0.00	Possibly absent
CG31305	CG31305-RA	1.46	0.00	Up
	CG31305-RB	0.84	0.00	Possibly absent
	CG31305-RD	0.75	0.00	Possibly absent
	CG31305-RF	0.90	0.00	Possibly absent
	CG31305-RG	0.63	0.77	Slightly down
	CG31305-RI	0.54	0.23	Slightly down
CG31332	CG31332-RA	3.59	0.00	Possibly absent
	CG31332-RB	1.53	1.00	Unchanged
	CG31332-RC	0.96	0.00	Unchanged
	CG31332-RD	5.88	0.00	Up
CG31764	CG31764-RA	1.44	0.01	Up
	CG31764-RB	0.65	0.02	Slightly down
	CG31764-RC	0.68	0.97	Slightly down
CG32423	CG32423-RA	0.90	0.38	Possibly absent
	CG32423-RB	1.01	0.00	Unchanged
	CG32423-RC	1.07	0.62	Possibly absent
	CG32423-RD	1.92	0.00	Up
CG33175	CG33175-RA	0.73	0.00	Slightly down
	CG33175-RG	1.39	0.00	Up
	CG33175-RH	1.14	1.00	Possibly absent
CG4376	CG4376-RA	1.24	1.00	Unchanged
	CG4376-RB	2.21	0.00	Up
	CG4376-RC	1.07	0.00	Possibly absent
CG4452	CG4452-RA	1.00	0.81	Unchanged
	CG4452-RB	704.16	0.00	Up
	CG4452-RC	0.88	0.19	Possibly absent
CG6854	CG6854-RA	1.36	0.00	Up
	CG6854-RB	0.73	0.14	Slightly down
	CG6854-RC	0.85	0.86	Possibly absent
CG6946	CG6946-RA	0.80	0.86	Slightly down
	CG6946-RB	0.94	0.00	Possibly absent
	CG6946-RC	4.53	0.14	Up
CG7283	CG7283-RA	0.83	0.91	Unchanged
	CG7283-RB	4.90	0.09	Up
	CG7283-RC	0.75	0.00	Possibly absent
