# Supplementary material for: Genome-Wide Identification of Alternative Splice Forms Down-Regulated by Nonsense-Mediated mRNA Decay in Drosophila
Source: PLoS Genet. 2009 Jun 19;5(6):e1000525. doi: 10.1371/journal.pgen.1000525 (PMC2689934; doi:10.1371/journal.pgen.1000525)
Supplement: Table S10 — Deconvolution results for the less stringent set of upf1 affected genes. (0.05 MB PDF) [file pgen.1000525.s032.pdf]

Table S10. Deconvolution results for the less stringent set of *upf1* affected genes

Gene	Transcript	alpha	beta	Call
CG10079	CG10079-RA	0.92	1.00	Unchanged
	CG10079-RB	1.51	0.00	Up
CG10126	CG10126-RA	1.35	0.42	Up
	CG10126-RB	0.80	0.58	Slightly down
CG10371	CG10371-RA	1.12	0.24	Up
	CG10371-RB	0.80	0.76	Slightly down
CG10494	CG10494-RA	0.91	1.00	Slightly down
	CG10494-RB	1.43	0.00	Up
CG10699	CG10699-RA	0.82	1.00	Slightly down
	CG10699-RB	1.46	0.00	Up
CG1088	CG1088-RA	0.99	0.65	Unchanged
	CG1088-RB	1.85	0.35	Up
CG10901	CG10901-RA	1.22	0.45	Up
	CG10901-RB	0.74	0.55	Slightly down
CG1104	CG1104-RA	1.31	0.35	Up
	CG1104-RB	0.89	0.65	Unchanged
CG11760	CG11760-RA	1.68	1.00	Up
	CG11760-RB	0.80	0.00	Slightly down
CG11779	CG11779-RA	1.14	1.00	Up
	CG11779-RB	0.68	0.00	Slightly down
CG1200	CG1200-RA	1.07	1.00	Unchanged
	CG1200-RB	1.60	0.00	Up
CG12101	CG12101-RA	1.49	0.00	Up
	CG12101-RB	1.10	1.00	Unchanged
CG12134	CG12134-RA	0.78	1.00	Slightly down
	CG12134-RB	1.43	0.00	Up
CG1233	CG1233-RA	1.33	0.30	Up
	CG1233-RB	0.82	0.70	Slightly down
CG12342	CG12342-RA	2.93	0.00	Up
	CG12342-RB	0.95	1.00	Unchanged
CG1263	CG1263-RA	1.09	1.00	Unchanged
	CG1263-RB	2.21	0.00	Up
CG12891	CG12891-RA	1.07	0.94	Unchanged
	CG12891-RB	1.86	0.06	Up
CG13204	CG13204-RA	0.83	0.64	Slightly down
	CG13204-RB	1.84	0.36	Up
CG1338	CG1338-RA	1.45	0.00	Up
	CG1338-RB	0.99	1.00	Unchanged
CG13521	CG13521-RA	1.10	1.00	Up
	CG13521-RB	0.67	0.00	Slightly down
CG1362	CG1362-RA	1.42	0.36	Up
	CG1362-RB	0.85	0.64	Unchanged
CG13900	CG13900-RA	3.13	0.46	Up
	CG13900-RB	1.03	0.54	Unchanged
CG13923	CG12022-RA	1.18	0.41	Up
	CG13923-RA	0.79	0.59	Slightly down
CG14444	CG14444-RA	1.13	0.92	Unchanged
	CG14444-RB	2.19	0.08	Up
CG1462	CG1462-RA	0.97	1.00	Unchanged
	CG1462-RB	1.34	0.00	Up
CG1486	CG1486-RA	0.94	0.73	Unchanged
	CG1486-RB	1.63	0.27	Up
CG1488	CG1488-RA	1.09	0.88	Unchanged
	CG1488-RB	1.73	0.12	Up
CG15117	CG15117-RA	1.27	1.00	Up
	CG15117-RB	0.71	0.00	Slightly down
CG15626	CG15626-RA	0.86	0.55	Slightly down
	CG15626-RB	1.42	0.45	Up
CG15881	CG15881-RA	0.82	1.00	Slightly down
	CG15881-RB	1.52	0.00	Up
CG1648	CG1648-RA	1.73	0.00	Up

Continued on next page

Table S10 – continued from previous page

Gene	Transcript	alpha	beta	Call
	CG1648-RB	0.99	1.00	Unchanged
CG16718	CG16718-RA	0.78	0.00	Slightly down
	CG16718-RB	1.17	1.00	Up
CG17035	CG17035-RA	0.87	0.86	Unchanged
	CG17035-RB	4.20	0.14	Up
CG18009	CG18009-RA	2.34	0.00	Up
	CG18009-RD	0.97	1.00	Unchanged
CG1866	CG1866-RA	0.74	0.56	Unchanged
	CG1866-RB	3.10	0.44	Up
CG1902	CG1902-RA	0.71	0.67	Slightly down
	CG1902-RC	1.49	0.33	Up
CG2152	CG2152-RA	0.75	0.97	Slightly down
	CG2152-RB	1.38	0.03	Up
CG2177	CG2177-RA	0.92	0.62	Unchanged
	CG2177-RB	1.25	0.38	Up
CG2179	CG2179-RA	2.60	0.54	Up
	CG2179-RB	1.01	0.46	Unchanged
CG30015	CG30015-RA	1.11	1.00	Up
	CG30015-RB	0.79	0.00	Slightly down
CG31751	CG31751-RA	1.07	1.00	Unchanged
	CG31751-RB	1.95	0.00	Up
CG3182	CG3182-RA	0.99	0.76	Unchanged
	CG3182-RB	1.55	0.24	Up
CG31961	CG31961-RA	1.15	0.27	Up
	CG31961-RB	0.82	0.73	Slightly down
CG32140	CG32140-RA	0.87	1.00	Unchanged
	CG32140-RB	1.62	0.00	Up
CG3217	CG3217-RA	1.00	1.00	Unchanged
	CG3217-RB	1.36	0.00	Up
CG3241	CG3241-RA	1.10	1.00	Unchanged
	CG3241-RB	1.50	0.00	Up
CG32647	CG32647-RA	1.59	0.00	Up
	CG32647-RB	0.98	1.00	Unchanged
CG33085	CG33085-RA	0.89	0.00	Slightly down
	CG33085-RD	1.28	1.00	Up
CG33206	CG33206-RA	0.89	0.78	Unchanged
	CG33206-RB	7.59	0.22	Up
CG3358	CG3358-RA	3.48	0.15	Up
	CG3358-RB	0.87	0.85	Unchanged
CG3530	CG3530-RA	0.72	0.16	Slightly down
	CG3530-RB	1.42	0.84	Up
CG3629	CG3629-RA	0.92	1.00	Slightly down
	CG3629-RB	12.96	0.00	Up
CG3731	CG3731-RA	1.50	0.13	Up
	CG3731-RB	0.87	0.87	Slightly down
CG3861	CG3861-RA	1.21	0.35	Up
	CG3861-RB	0.67	0.65	Slightly down
CG4059	CG4059-RA	5.15	0.04	Up
	CG4059-RB	0.74	0.96	Slightly down
CG4070	CG4070-RA	0.79	0.13	Slightly down
	CG4070-RB	1.19	0.87	Up
CG4143	CG4143-RA	1.14	1.00	Unchanged
	CG4143-RB	1.85	0.00	Up
CG4247	CG4247-RA	0.59	1.00	Slightly down
	CG4247-RB	1.34	0.00	Up
CG4590	CG4590-RA	0.77	0.28	Slightly down
	CG4590-RB	1.22	0.72	Up
CG4609	CG4609-RA	1.98	0.36	Up
	CG4609-RB	1.07	0.64	Unchanged
CG4673	CG4673-RA	0.90	0.56	Unchanged
	CG4673-RB	1.81	0.44	Up

Continued on next page

Table S10 – continued from previous page

Gene	Transcript	alpha	beta	Call
CG4712	CG4712-RA	0.89	1.00	Slightly down
	CG4712-RB	1.40	0.00	Up
CG4795	CG4795-RA	0.98	1.00	Unchanged
	CG4795-RB	1.49	0.00	Up
CG5081	CG5081-RA	1.12	1.00	Up
	CG5081-RB	0.80	0.00	Slightly down
CG5215	CG5215-RA	4.17	0.32	Up
	CG5215-RB	0.98	0.68	Unchanged
CG5326	CG5326-RA	0.83	1.00	Slightly down
	CG5326-RB	1.38	0.00	Up
CG5394	CG5394-RA	1.28	0.00	Up
	CG5394-RB	0.97	1.00	Unchanged
CG5613	CG5613-RA	0.91	0.72	Unchanged
	CG5613-RB	1.60	0.28	Up
CG5625	CG5625-RA	1.23	0.87	Up
	CG5625-RB	0.77	0.13	Slightly down
CG5729	CG5729-RA	0.66	0.01	Slightly down
	CG5729-RB	10.30	0.99	Up
CG5785	CG5785-RA	0.85	1.00	Slightly down
	CG5785-RB	1.41	0.00	Up
CG5854	CG5854-RA	1.32	0.08	Up
	CG5854-RB	1.00	0.92	Unchanged
CG5896	CG5896-RA	3.47	0.03	Up
	CG5896-RB	0.80	0.97	Slightly down
CG6023	CG6023-RA	0.78	0.96	Slightly down
	CG6023-RB	1.50	0.04	Up
CG6084	CG6084-RA	0.54	0.56	Slightly down
	CG6084-RB	1.29	0.44	Up
CG6090	CG6090-RA	0.56	1.00	Slightly down
	CG6090-RB	1.32	0.00	Up
CG6297	CG6297-RA	1.41	0.00	Up
	CG6297-RB	1.00	1.00	Unchanged
CG6315	CG6315-RA	1.01	0.75	Unchanged
	CG6315-RB	2.71	0.25	Up
CG6454	CG6454-RA	7.77	0.01	Up
	CG6454-RB	1.05	0.99	Unchanged
CG6608	CG6608-RA	1.05	1.00	Unchanged
	CG6608-RB	1.41	0.00	Up
CG6726	CG6726-RA	1.20	1.00	Up
	CG6726-RB	0.73	0.00	Slightly down
CG6767	CG6767-RA	1.24	0.00	Up
	CG6767-RB	0.93	1.00	Slightly down
CG6798	CG6798-RA	1.27	0.62	Up
	CG6798-RB	0.84	0.38	Slightly down
CG6829	CG6829-RA	0.89	0.65	Unchanged
	CG6829-RB	1.32	0.35	Up
CG6891	CG6891-RA	1.06	1.00	Unchanged
	CG6891-RB	1.26	0.00	Up
CG7070	CG7070-RA	0.77	0.00	Slightly down
	CG7070-RB	1.23	1.00	Up
CG7263	CG7263-RA	1.45	0.33	Up
	CG7263-RB	0.78	0.67	Slightly down
CG7334	CG7334-RA	1.21	0.98	Up
	CG7334-RB	0.81	0.02	Slightly down
CG7540	CG7540-RA	1.27	0.70	Up
	CG7540-RB	0.66	0.30	Slightly down
CG7725	CG7725-RA	1.38	1.00	Up
	CG7725-RB	0.86	0.00	Slightly down
CG7908	CG7908-RA	0.96	1.00	Unchanged
	CG7908-RB	1.29	0.00	Up
CG8318	CG8318-RB	1.08	1.00	Up

Continued on next page

Table S10 – continued from previous page

Gene	Transcript	alpha	beta	Call
	CG8318-RC	0.61	0.00	Slightly down
CG8327	CG8327-RA	0.59	0.92	Slightly down
	CG8327-RB	1.28	0.08	Up
CG8332	CG8332-RA	0.59	1.00	Slightly down
	CG8332-RB	4.37	0.00	Up
CG8376	CG8376-RA	0.86	0.89	Slightly down
	CG8376-RB	1.34	0.11	Up
CG8430	CG8430-RA	2.00	0.53	Up
	CG8430-RB	0.83	0.47	Slightly down
CG8486	CG8486-RA	0.73	0.00	Slightly down
	CG8486-RB	1.34	1.00	Up
CG8495	CG8495-RA	1.87	0.17	Up
	CG8495-RC	0.83	0.83	Unchanged
CG8557	CG8557-RA	1.07	1.00	Up
	CG8557-RB	0.75	0.00	Slightly down
CG8765	CG8765-RA	1.58	0.01	Up
	CG8765-RB	1.12	0.99	Unchanged
CG8783	CG8783-RA	1.49	0.18	Up
	CG8783-RB	0.85	0.82	Slightly down
CG8811	CG8811-RA	1.49	0.19	Up
	CG8811-RB	0.86	0.81	Unchanged
CG8944	CG8944-RA	1.02	0.97	Unchanged
	CG8944-RB	2.19	0.03	Up
CG8956	CG8956-RC	1.42	0.27	Up
	CG8956-RD	0.84	0.73	Slightly down
CG9195	CG9195-RA	1.47	1.00	Up
	CG9195-RB	0.78	0.00	Slightly down
CG9248	CG9248-RA	0.61	0.00	Slightly down
	CG9248-RB	1.22	1.00	Up
CG9256	CG9256-RA	1.07	1.00	Up
	CG9256-RB	0.67	0.00	Slightly down
CG9354	CG9354-RA	3.84	0.00	Up
	CG9354-RB	0.66	1.00	Slightly down
CG9413	CG9413-RA	1.15	0.95	Up
	CG9413-RB	0.59	0.05	Slightly down
CG9415	CG9415-RA	1.48	0.70	Up
	CG9415-RB	0.73	0.30	Slightly down
CG9425	CG9425-RA	0.73	0.40	Slightly down
	CG9425-RB	1.37	0.60	Up
CG9611	CG9611-RA	0.81	0.39	Slightly down
	CG9611-RB	1.47	0.61	Up
CR32885	CR32885-RA	0.80	0.83	Slightly down
	CR32885-RB	1.23	0.17	Up
CG10023	CG10023-RA	1.18	1.00	Up
	CG10023-RB	0.75	0.00	Slightly down
	CG10023-RC	0.92	0.00	Possibly absent
CG10107	CG10107-RA	2.75	0.27	Up
	CG10107-RB	0.80	0.73	Unchanged
	CG10107-RC	1.87	0.00	Up
CG10121	CG10121-RA	0.74	0.28	Slightly down
	CG10121-RB	1.37	0.00	Up
	CG10121-RC	0.91	0.63	Possibly absent
	CG10121-RD	1.23	0.09	Possibly absent
CG10772	CG10772-RA	1.18	0.00	Possibly absent
	CG10772-RB	0.85	0.02	Possibly absent
	CG10772-RC	2.78	0.05	Up
	CG10772-RD	1.17	0.89	Unchanged
	CG10772-RE	1.18	0.04	Possibly absent
	CG10772-RF	1.03	0.00	Possibly absent
CG10868	CG10868-RA	0.89	0.31	Slightly down
	CG10868-RB	0.66	0.27	Slightly down

Continued on next page

Table S10 – continued from previous page

Gene	Transcript	alpha	beta	Call
	CG10868-RC	1.33	0.43	Up
CG10948	CG10948-RA	0.97	0.00	Possibly absent
	CG10948-RB	2.94	0.15	Up
	CG10948-RC	1.18	0.85	Unchanged
CG11081	CG11081-RA	1.16	1.00	Up
	CG11081-RB	0.83	0.00	Possibly absent
	CG11081-RC	0.95	0.00	Possibly absent
	CG11081-RD	0.70	0.00	Slightly down
CG11100	CG11100-RA	0.82	0.07	Slightly down
	CG11100-RB	0.87	0.68	Slightly down
	CG11100-RC	1.31	0.25	Up
CG11163	CG11163-RA	1.23	0.00	Up
	CG11163-RB	1.35	0.00	Up
	CG11163-RC	1.07	0.00	Unchanged
	CG11163-RD	1.34	1.00	Up
CG11537	CG11537-RA	0.80	0.00	Slightly down
	CG11537-RB	1.01	0.00	Possibly absent
	CG11537-RC	1.14	1.00	Up
CG12085	CG12085-RA	0.96	0.00	Possibly absent
	CG12085-RB	1.10	0.64	Unchanged
	CG12085-RC	1.40	0.00	Possibly absent
	CG12085-RD	2.05	0.36	Up
CG1213	CG1213-RA	1.24	0.26	Up
	CG1213-RB	0.75	0.67	Slightly down
	CG1213-RC	0.75	0.07	Slightly down
CG12746	CG12746-RA	1.04	0.00	Possibly absent
	CG12746-RB	1.20	1.00	Up
	CG12746-RC	0.69	0.00	Possibly absent
	CG12746-RD	0.72	0.00	Slightly down
CG14217	CG14217-RA	0.83	0.00	Unchanged
	CG14217-RB	2.74	0.00	Up
	CG14217-RD	0.82	0.00	Possibly absent
	CG14217-RE	1.05	1.00	Possibly absent
CG14414	CG14414-RA	1.97	0.36	Up
	CG14414-RB	1.09	0.64	Unchanged
	CG14414-RC	0.92	0.00	Possibly absent
CG14823	CG14823-RA	0.70	0.01	Slightly down
	CG14823-RB	0.00	0.08	Slightly down
	CG14823-RC	1.24	0.17	Up
	CG14823-RD	0.91	0.74	Unchanged
CG14938	CG14938-RA	0.93	0.76	Unchanged
	CG14938-RB	1.41	0.02	Up
	CG14938-RC	0.81	0.00	Possibly absent
	CG14938-RD	0.82	0.21	Slightly down
CG1623	CG1623-RA	1.56	0.00	Up
	CG1623-RC	2.38	0.00	Up
	CG1623-RE	0.96	1.00	Unchanged
CG1634	CG1634-RA	0.89	0.51	Possibly absent
	CG1634-RB	0.70	0.00	Slightly down
	CG1634-RC	1.32	0.49	Up
CG16833	CG16833-RA	1.17	0.42	Up
	CG16833-RB	0.79	0.00	Slightly down
	CG16833-RC	0.98	0.58	Possibly absent
CG16901	CG16901-RA	1.03	0.70	Possibly absent
	CG16901-RB	0.96	0.18	Unchanged
	CG16901-RC	0.79	0.12	Possibly absent
	CG16901-RD	2.45	0.00	Up
CG16952	CG16952-RA	0.74	0.01	Slightly down
	CG16952-RB	1.15	0.99	Up
	CG16952-RC	1.10	0.00	Possibly absent
CG16973	CG16973-RA	1.64	0.16	Up

Continued on next page

Table S10 – continued from previous page

Gene	Transcript	alpha	beta	Call
	CG16973-RB	1.01	0.83	Unchanged
	CG16973-RC	0.92	0.00	Possibly absent
	CG16973-RD	1.57	0.01	Up
	CG16973-RE	0.98	0.00	Possibly absent
CG17332	CG17332-RA	1.39	0.68	Up
	CG17332-RB	0.78	0.00	Slightly down
	CG17332-RD	1.09	0.32	Possibly absent
CG17834	CG17834-RA	0.76	0.00	Slightly down
	CG17834-RB	1.41	0.99	Up
	CG17834-RC	1.14	0.00	Possibly absent
	CG17834-RD	0.79	0.01	Possibly absent
CG18069	CG18069-RA	0.96	0.00	Possibly absent
	CG18069-RB	1.29	1.00	Up
	CG18069-RC	0.93	0.00	Unchanged
CG18769	CG18769-RA	0.87	0.00	Possibly absent
	CG18769-RB	0.74	0.00	Slightly down
	CG18769-RC	0.97	0.74	Possibly absent
	CG18769-RD	1.04	0.13	Possibly absent
	CG18769-RE	0.88	0.00	Possibly absent
	CG18769-RF	1.37	0.14	Up
CG1877	CG1877-RA	1.49	0.00	Up
	CG1877-RB	1.21	1.00	Possibly absent
	CG1877-RC	1.07	0.00	Possibly absent
	CG1877-RD	0.97	0.00	Unchanged
CG2216	CG2216-RA	1.59	0.00	Up
	CG2216-RB	5.02	0.14	Up
	CG2216-RC	2.17	0.05	Up
	CG2216-RD	1.08	0.42	Unchanged
	CG2216-RE	0.97	0.38	Possibly absent
CG2225	CG2225-RA	0.94	0.03	Possibly absent
	CG2225-RB	21.02	0.00	Possibly absent
	CG2225-RC	1.08	0.86	Up
	CG2225-RD	0.72	0.10	Slightly down
	CG2225-RE	1.10	0.01	Possibly absent
CG2304	CG2304-RA	1.39	0.78	Up
	CG2304-RB	0.00	0.09	Slightly down
	CG2304-RC	1.13	0.00	Possibly absent
	CG2304-RD	1.39	0.13	Up
CG31045	CG31045-RA	1.18	0.05	Up
	CG31045-RB	1.08	0.01	Possibly absent
	CG31045-RC	1.09	0.00	Possibly absent
	CG31045-RD	0.88	0.90	Slightly down
	CG31045-RE	0.78	0.04	Possibly absent
CG31237	CG31237-RA	0.86	1.00	Slightly down
	CG31318-RA	1.85	0.00	Up
	CG31318-RB	1.34	0.00	Up
CG31305	CG31305-RA	1.46	0.00	Up
	CG31305-RB	0.84	0.00	Possibly absent
	CG31305-RD	0.75	0.00	Possibly absent
	CG31305-RF	0.90	0.00	Possibly absent
	CG31305-RG	0.63	0.77	Slightly down
	CG31305-RI	0.54	0.23	Slightly down
CG31332	CG31332-RA	3.59	0.00	Up
	CG31332-RB	1.53	1.00	Up
	CG31332-RC	0.96	0.00	Unchanged
	CG31332-RD	5.88	0.00	Up
CG31536	CG31536-RA	2087.70	0.00	Up
	CG31536-RB	0.77	0.32	Slightly down
	CG31536-RC	1.25	0.68	Up
CG31764	CG31764-RA	1.44	0.01	Up
	CG31764-RB	0.65	0.02	Slightly down

Continued on next page

Gene	Transcript	alpha	beta	Call
	CG31764-RC	0.68	0.97	Slightly down
CG32018	CG32018-RA	1.13	0.00	Possibly absent
	CG32018-RB	1.40	0.00	Up
	CG32018-RC	1.53	0.00	Up
	CG32018-RD	1.01	0.29	Possibly absent
	CG32018-RE	1.00	0.44	Unchanged
	CG32018-RF	1.05	0.26	Possibly absent
	CG32018-RG	1.14	0.00	Possibly absent
CG32423	CG32423-RA	0.90	0.38	Possibly absent
	CG32423-RB	1.01	0.00	Unchanged
	CG32423-RC	1.07	0.62	Possibly absent
	CG32423-RD	1.92	0.00	Up
CG32508	CG32508-RA	1.52	0.00	Up
	CG32508-RB	0.80	0.30	Slightly down
	CG32508-RC	1.09	0.70	Unchanged
CG32538	CG32538-RA	2.05	0.00	Up
	CG32538-RB	0.81	0.00	Slightly down
	CG32538-RC	0.88	1.00	Slightly down
CG32858	CG32858-RA	0.98	1.00	Unchanged
	CG32858-RB	1.47	0.00	Up
	CG32858-RC	0.94	0.00	Possibly absent
CG33054	CG33054-RA	0.67	0.59	Slightly down
	CG33054-RB	0.91	0.00	Possibly absent
	CG33056-RA	1.01	0.00	Possibly absent
	CG33056-RB	1.45	0.40	Up
	CG33056-RC	0.86	0.00	Slightly down
	CG33056-RD	3.26	0.00	Up
	CG33056-RE	1.06	0.01	Possibly absent
CG33129	CG33129-RA	0.48	0.79	Slightly down
	CG33129-RB	0.56	0.00	Slightly down
	CG33129-RC	0.83	0.00	Possibly absent
	CG33129-RE	1.08	0.21	Up
CG33184	CG33184-RA	0.66	1.00	Slightly down
	CG33184-RB	1.18	0.00	Possibly absent
	CG33184-RC	1.43	0.00	Up
CG33261	CG33261-RA	0.00	0.00	Slightly down
	CG33261-RB	0.87	0.00	Possibly absent
	CG33261-RC	0.28	0.52	Slightly down
	CG33261-RD	0.72	0.48	Unchanged
	CG33261-RE	0.61	0.00	Slightly down
	CG33261-RF	1.57	0.00	Up
CG3638	CG3638-RA	0.95	0.00	Unchanged
	CG3638-RB	0.62	0.00	Slightly down
	CG3638-RC	1.90	0.02	Up
	CG3638-RD	1.00	0.98	Unchanged
CG3948	CG3948-RA	0.45	0.83	Slightly down
	CG3948-RB	0.78	0.00	Slightly down
	CG3948-RC	1.28	0.17	Up
CG4016	CG4016-RA	0.76	0.25	Slightly down
	CG4016-RB	1.15	0.56	Up
	CG4016-RC	0.86	0.19	Possibly absent
CG4239	CG4239-RA	1.53	0.00	Up
	CG4239-RB	1.68	0.01	Up
	CG4239-RC	0.98	0.99	Unchanged
CG4376	CG4376-RA	1.24	1.00	Unchanged
	CG4376-RB	2.21	0.00	Up
	CG4376-RC	1.07	0.00	Possibly absent
CG4389	CG4389-RA	1.49	0.77	Up
	CG4389-RB	0.79	0.23	Slightly down
	CG4389-RC	0.81	0.00	Possibly absent
CG4452	CG4452-RA	1.00	0.81	Unchanged

Continued on next page

Table S10 – continued from previous page

Gene	Transcript	alpha	beta	Call
	CG4452-RB	704.16	0.00	Up
	CG4452-RC	0.88	0.19	Possibly absent
CG4816	CG4816-RA	897.26	0.00	Up
	CG4816-RB	1.08	0.61	Unchanged
	CG4816-RC	1.01	0.39	Possibly absent
CG4921	CG4921-RA	0.00	0.28	Slightly down
	CG4921-RB	1.14	0.26	Possibly absent
	CG4921-RC	1.32	0.46	Up
CG5288	CG5288-RA	0.45	0.47	Slightly down
	CG5288-RB	1.23	0.04	Up
	CG5288-RC	1.32	0.48	Up
CG5461	CG5461-RA	1.41	0.00	Up
	CG5461-RB	0.88	0.00	Unchanged
	CG5461-RC	1.01	1.00	Possibly absent
CG5486	CG5486-RA	0.68	0.54	Slightly down
	CG5486-RB	1.28	0.46	Up
	CG5486-RC	0.91	0.00	Possibly absent
CG6854	CG6854-RA	1.36	0.00	Up
	CG6854-RB	0.73	0.14	Slightly down
	CG6854-RC	0.85	0.86	Possibly absent
CG6946	CG6946-RA	0.80	0.86	Slightly down
	CG6946-RB	0.94	0.00	Possibly absent
	CG6946-RC	4.53	0.14	Up
CG7176	CG7176-RA	0.87	0.00	Possibly absent
	CG7176-RB	0.98	0.00	Possibly absent
	CG7176-RC	0.96	0.19	Possibly absent
	CG7176-RD	0.82	0.00	Slightly down
	CG7176-RE	0.04	0.10	Possibly absent
	CG7176-RF	1.28	0.71	Up
	CG7176-RG	1.12	0.00	Possibly absent
CG7283	CG7283-RA	0.83	0.91	Unchanged
	CG7283-RB	4.90	0.09	Up
	CG7283-RC	0.75	0.00	Possibly absent
CG7555	CG7555-RA	1.07	0.00	Possibly absent
	CG7555-RB	0.85	0.00	Slightly down
	CG7555-RC	0.91	0.07	Possibly absent
	CG7555-RD	1.11	0.93	Up
CG7926	CG7926-RA	87.05	0.00	Up
	CG7926-RB	0.97	0.84	Unchanged
	CG7926-RC	0.86	0.16	Possibly absent
CG8121	CG8121-RA	1.19	0.74	Up
	CG8121-RB	0.67	0.26	Slightly down
	CG8121-RC	1.30	0.00	Up
CG8127	CG8127-RA	0.85	0.00	Slightly down
	CG8127-RB	1.09	0.96	Up
	CG8127-RC	0.87	0.00	Possibly absent
	CG8127-RD	0.89	0.04	Possibly absent
CG8178	CG8178-RA	0.93	0.00	Possibly absent
	CG8178-RB	1.89	0.00	Up
	CG8178-RC	0.92	1.00	Slightly down
CG8363	CG8363-RA	1.20	0.00	Possibly absent
	CG8363-RB	1.04	0.00	Possibly absent
	CG8363-RC	1.00	0.01	Possibly absent
	CG8363-RD	0.84	0.94	Slightly down
	CG8363-RE	1.28	0.04	Up
CG8440	CG8440-RA	1.15	0.34	Possibly absent
	CG8440-RB	1.01	0.00	Possibly absent
	CG8440-RC	1.45	0.24	Up
	CG8440-RD	1.15	0.41	Up
	CG8440-RE	0.73	0.02	Slightly down
	CG8440-RF	1.13	0.00	Possibly absent

Continued on next page

Table S10 – continued from previous page

Gene	Transcript	alpha	beta	Call
CG8465	CG8465-RA	1.18	0.42	Up
	CG8465-RB	0.94	0.16	Possibly absent
	CG8465-RC	0.77	0.43	Slightly down
CG8663	CG8663-RA	1.10	0.00	Possibly absent
	CG8663-RB	1.07	0.92	Up
	CG8663-RC	1.09	0.00	Possibly absent
	CG8663-RD	0.81	0.08	Slightly down
CG8732	CG8732-RA	1.07	1.00	Up
	CG8732-RB	0.68	0.00	Slightly down
	CG8732-RC	0.89	0.00	Possibly absent
CG9277	CG9277-RA	0.80	0.63	Slightly down
	CG9277-RB	1.13	0.37	Up
	CG9277-RC	0.80	0.00	Possibly absent
	CG9277-RD	0.96	0.00	Possibly absent
CG9381	CG9381-RA	1.31	0.98	Up
	CG9381-RB	0.65	0.02	Slightly down
	CG9381-RC	1.11	0.00	Possibly absent
CG9755	CG9755-RA	0.78	0.00	Slightly down
	CG9755-RB	1.00	0.00	Possibly absent
	CG9755-RC	1.35	0.00	Up
	CG9755-RD	1.27	0.00	Possibly absent
	CG9755-RE	1.15	1.00	Up
CG9772	CG9772-RA	1.11	0.76	Up
	CG9772-RB	0.93	0.00	Possibly absent
	CG9772-RC	0.86	0.24	Slightly down
