# Supplementary material for: Genome-Wide Identification of Alternative Splice Forms Down-Regulated by Nonsense-Mediated mRNA Decay in Drosophila
Source: PLoS Genet. 2009 Jun 19;5(6):e1000525. doi: 10.1371/journal.pgen.1000525 (PMC2689934; doi:10.1371/journal.pgen.1000525)
Supplement: Table S11 — Deconvolution results for the stringent set of upf2 affected genes. (0.03 MB PDF) [file pgen.1000525.s033.pdf]

Table S11. Deconvolution results for the stringent set of *upf2* affected genes

Gene	Transcript	alpha	beta	Call
CG33206	CG33206-RA	0.66	0.83	Slightly down
	CG33206-RB	4.94	0.17	Up
CG3321	CG3321-RA	1.38	0.93	Up
	CG3321-RB	0.59	0.07	Slightly down
CG3358	CG3358-RA	1.75	0.37	Up
	CG3358-RB	0.60	0.63	Slightly down
CG3629	CG3629-RA	1.12	0.98	Unchanged
	CG3629-RB	3.69	0.02	Up
CG5215	CG5215-RA	3.77	0.27	Up
	CG5215-RB	0.94	0.73	Unchanged
CG5896	CG5896-RA	3.04	0.01	Up
	CG5896-RB	0.74	0.99	Slightly down
CG6359	CG6359-RA	1.15	1.00	Up
	CG6359-RB	0.65	0.00	Slightly down
CG6454	CG6454-RA	3.35	0.01	Up
	CG6454-RB	1.23	0.99	Unchanged
CG7766	CG7766-RA	1.40	0.05	Up
	CG7766-RB	0.97	0.95	Unchanged
CG8332	CG8332-RA	1.08	0.98	Unchanged
	CG8332-RB	3.13	0.02	Up
CG8857	CG8857-RA	0.75	0.98	Slightly down
	CG8857-RB	1.64	0.02	Up
CG17077	CG17077-RB	1.49	0.00	Up
	CG17077-RC	0.86	1.00	Possibly absent
	CG17077-RD	0.65	0.00	Slightly down
CG31332	CG31332-RA	1.15	0.51	Possibly absent
	CG31332-RB	0.81	0.49	Unchanged
	CG31332-RC	0.65	0.00	Unchanged
	CG31332-RD	2.81	0.00	Up
CG32149	CG32149-RA	0.81	0.35	Possibly absent
	CG32149-RB	0.61	0.14	Slightly down
	CG32149-RC	1.30	0.52	Up
CG33261	CG33261-RA	0.00	0.00	Slightly down
	CG33261-RB	0.91	0.92	Possibly absent
	CG33261-RC	0.00	0.07	Possibly absent
	CG33261-RD	17.48	0.00	Possibly absent
	CG33261-RE	0.76	0.00	Possibly absent
	CG33261-RF	1.68	0.00	Up
CG3671	CG3671-RA	0.66	0.46	Unchanged
	CG3671-RB	1.32	0.36	Up
	CG3671-RC	1.29	0.18	Possibly absent
CG4452	CG4452-RA	0.82	0.70	Slightly down
	CG4452-RB	1.84	0.29	Up
	CG4452-RC	0.52	0.01	Slightly down
CG6946	CG6946-RA	0.64	0.73	Slightly down
	CG6946-RB	1.03	0.00	Possibly absent
	CG6946-RC	3.36	0.27	Up
