# Supplementary material for: Genome-Wide Identification of Alternative Splice Forms Down-Regulated by Nonsense-Mediated mRNA Decay in Drosophila
Source: PLoS Genet. 2009 Jun 19;5(6):e1000525. doi: 10.1371/journal.pgen.1000525 (PMC2689934; doi:10.1371/journal.pgen.1000525)
Supplement: Table S12 — Deconvolution results for the less stringent set of upf2 affected genes. (0.04 MB PDF) [file pgen.1000525.s034.pdf]

Table S12. Deconvolution results for the less stringent set of *upf2* affected genes

Gene	Transcript	alpha	beta	Call
CG1104	CG1104-RA	1.47	0.28	Up
	CG1104-RB	0.90	0.72	Unchanged
CG1109	CG1109-RA	1.06	1.00	Up
	CG1109-RB	0.61	0.00	Slightly down
CG11328	CG11328-RA	0.84	0.00	Slightly down
	CG11328-RB	1.08	1.00	Up
CG11961	CG11961-RA	1.01	1.00	Unchanged
	CG11961-RB	1.29	0.00	Up
CG12134	CG12134-RA	0.64	0.89	Slightly down
	CG12134-RB	1.72	0.11	Up
CG12864	CG12864-RA	1.29	0.28	Up
	CG12864-RB	0.92	0.72	Unchanged
CG13900	CG13900-RA	2.84	0.48	Up
	CG13900-RB	1.04	0.52	Unchanged
CG13923	CG12022-RA	1.35	0.00	Up
	CG13923-RA	0.98	1.00	Unchanged
CG1546	CG1546-RA	0.97	0.96	Unchanged
	CG1546-RB	1.53	0.04	Up
CG17035	CG17035-RA	0.70	0.83	Unchanged
	CG17035-RB	2.14	0.17	Up
CG17369	CG17369-RA	0.71	0.00	Slightly down
	CG17369-RB	1.21	1.00	Up
CG1771	CG1771-RA	0.71	0.00	Slightly down
	CG1771-RB	1.11	1.00	Up
CG1866	CG1866-RA	1.00	0.56	Unchanged
	CG1866-RB	2.75	0.44	Up
CG2182	CG2182-RA	1.24	1.00	Up
	CG2182-RB	0.54	0.00	Slightly down
CG2534	CG2534-RA	1.54	0.48	Up
	CG2534-RB	1.00	0.52	Unchanged
CG31619	CG31619-RA	1.34	0.34	Up
	CG31619-RB	0.96	0.66	Unchanged
CG32527	CG32527-RA	0.73	0.00	Slightly down
	CG32527-RB	1.59	1.00	Up
CG33103	CG33103-RA	1.32	0.54	Up
	CG33103-RB	0.73	0.46	Slightly down
CG33206	CG33206-RA	0.66	0.83	Slightly down
	CG33206-RB	4.94	0.17	Up
CG3321	CG3321-RA	1.38	0.93	Up
	CG3321-RB	0.59	0.07	Slightly down
CG3358	CG3358-RA	1.75	0.37	Up
	CG3358-RB	0.60	0.63	Slightly down
CG3629	CG3629-RA	1.12	0.98	Unchanged
	CG3629-RB	3.69	0.02	Up
CG3941	CG3941-RA	1.17	1.00	Up
	CG3941-RB	0.67	0.00	Slightly down
CG4062	CG4062-RA	1.12	1.00	Up
	CG4062-RB	0.76	0.00	Slightly down
CG4482	CG4482-RA	1.33	0.10	Up
	CG4482-RB	0.87	0.90	Unchanged
CG4879	CG4879-RA	0.64	0.31	Slightly down
	CG4879-RB	1.30	0.69	Up
CG5081	CG5081-RA	1.10	1.00	Up
	CG5081-RB	0.74	0.00	Slightly down
CG5215	CG5215-RA	3.77	0.27	Up
	CG5215-RB	0.94	0.73	Unchanged
CG5729	CG5729-RA	0.61	0.00	Slightly down
	CG5729-RB	3.05	1.00	Up
CG5854	CG5854-RA	1.09	1.00	Unchanged
	CG5854-RB	1.68	0.00	Up
CG5896	CG5896-RA	3.04	0.01	Up

Continued on next page

Table S12 – continued from previous page

Gene	Transcript	alpha	beta	Call
	CG5896-RB	0.74	0.99	Slightly down
CG6359	CG6359-RA	1.15	1.00	Up
	CG6359-RB	0.65	0.00	Slightly down
CG6454	CG6454-RA	3.35	0.01	Up
	CG6454-RB	1.23	0.99	Unchanged
CG7602	CG7602-RA	0.77	0.00	Slightly down
	CG7602-RB	1.22	1.00	Up
CG7766	CG7766-RA	1.40	0.05	Up
	CG7766-RB	0.97	0.95	Unchanged
CG8332	CG8332-RA	1.08	0.98	Unchanged
	CG8332-RB	3.13	0.02	Up
CG8478	CG8478-RA	0.84	1.00	Slightly down
	CG8478-RB	1.69	0.00	Up
CG8811	CG8811-RA	1.55	0.23	Up
	CG8811-RB	0.88	0.77	Unchanged
CG8857	CG8857-RA	0.75	0.98	Slightly down
	CG8857-RB	1.64	0.02	Up
CG8983	CG8983-RA	1.37	1.00	Up
	CG8983-RB	0.68	0.00	Slightly down
CG9153	CG9153-RA	1.08	1.00	Up
	CG9153-RB	0.58	0.00	Slightly down
CG9354	CG9354-RA	2.80	0.02	Up
	CG9354-RB	0.95	0.98	Unchanged
CG10107	CG10107-RA	2.31	0.39	Up
	CG10107-RB	0.92	0.61	Unchanged
	CG10107-RC	1.18	0.00	Possibly absent
CG10772	CG10772-RA	1.33	0.75	Possibly absent
	CG10772-RB	0.99	0.00	Possibly absent
	CG10772-RC	0.96	0.00	Possibly absent
	CG10772-RD	0.88	0.25	Possibly absent
	CG10772-RE	1.54	0.00	Up
	CG10772-RF	0.70	0.00	Slightly down
CG14025	CG14025-RA	1.03	0.00	Possibly absent
	CG14025-RB	1.73	0.40	Up
	CG14025-RC	0.84	0.60	Unchanged
CG14414	CG14414-RA	1.69	0.61	Up
	CG14414-RB	0.75	0.39	Slightly down
	CG14414-RC	0.83	0.00	Possibly absent
CG1447	CG1447-RA	0.92	0.00	Possibly absent
	CG1447-RB	0.82	0.77	Slightly down
	CG1447-RC	1.25	0.23	Up
CG14792	CG14792-RA	1.99	0.07	Up
	CG14792-RB	0.66	0.93	Slightly down
	CG14792-RD	1.26	0.00	Possibly absent
CG14823	CG14823-RA	0.71	0.00	Possibly absent
	CG14823-RB	0.00	0.19	Slightly down
	CG14823-RC	1.54	0.23	Up
	CG14823-RD	0.98	0.59	Possibly absent
CG15086	CG15086-RA	1.00	0.62	Unchanged
	CG15086-RB	0.97	0.00	Possibly absent
	CG15086-RC	0.72	0.38	Possibly absent
	CG15086-RD	1.56	0.00	Up
CG1651	CG1651-RA	0.80	0.03	Possibly absent
	CG1651-RB	0.53	0.00	Slightly down
	CG1651-RC	1.20	0.97	Up
	CG1651-RD	0.77	0.00	Possibly absent
CG17077	CG17077-RB	1.49	0.00	Up
	CG17077-RC	0.86	1.00	Slightly down
	CG17077-RD	0.65	0.00	Slightly down
CG17299	CG17299-RA	1.32	0.19	Up
	CG17299-RB	0.72	0.00	Slightly down

Continued on next page

Table S12 – continued from previous page

Gene	Transcript	alpha	beta	Call
	CG17299-RC	0.80	0.00	Possibly absent
	CG17299-RD	0.71	0.01	Slightly down
	CG17299-RE	1.16	0.62	Up
	CG17299-RF	0.92	0.17	Possibly absent
	CG17299-RG	1.36	0.01	Up
	CG17299-RH	0.94	0.00	Possibly absent
CG17952	CG17952-RA	0.70	0.05	Possibly absent
	CG17952-RB	0.76	0.94	Slightly down
	CG17952-RC	1.90	0.01	Up
CG18660	CG18660-RA	1.07	0.59	Possibly absent
	CG18660-RB	0.00	0.18	Slightly down
	CG18660-RC	1.36	0.23	Up
CG2040	CG2040-RA	0.97	0.88	Possibly absent
	CG2040-RB	0.76	0.12	Unchanged
	CG2040-RC	1.93	0.00	Up
CG2098	CG2098-RA	0.00	0.02	Slightly down
	CG2098-RB	1.14	0.93	Up
	CG2098-RC	1.17	0.05	Possibly absent
CG2168	CG2168-RA	1.18	0.75	Up
	CG2168-RB	0.94	0.13	Possibly absent
	CG2168-RD	0.71	0.12	Slightly down
CG31137	CG31137-RA	0.84	0.00	Possibly absent
	CG31137-RB	1.13	1.00	Up
	CG31137-RC	0.86	0.00	Possibly absent
	CG31137-RD	0.99	0.00	Possibly absent
	CG31137-RE	1.13	0.00	Possibly absent
	CG31137-RF	0.76	0.00	Slightly down
CG31363	CG31363-RA	1.09	0.60	Possibly absent
	CG31363-RB	0.94	0.01	Possibly absent
	CG31363-RC	0.97	0.00	Unchanged
	CG31363-RD	2.07	0.30	Up
	CG31363-RE	0.63	0.00	Slightly down
	CG31363-RH	0.92	0.09	Possibly absent
CG32103	CG32103-RA	0.71	0.00	Slightly down
	CG32103-RB	1.26	0.01	Up
	CG32103-RC	0.95	0.99	Unchanged
CG32149	CG32149-RA	0.81	0.35	Possibly absent
	CG32149-RB	0.61	0.14	Slightly down
	CG32149-RC	1.30	0.52	Up
CG32858	CG32858-RA	2.10	0.00	Up
	CG32858-RB	0.97	1.00	Unchanged
	CG32858-RC	1.06	0.00	Possibly absent
CG33045	CG33045-RA	0.82	0.00	Possibly absent
	CG33045-RC	1.16	0.00	Possibly absent
	CG33045-RD	0.97	0.16	Possibly absent
	CG33045-RE	3.93	0.11	Up
	CG33045-RF	0.53	0.00	Possibly absent
	CG33045-RG	0.74	0.74	Slightly down
CG33129	CG33129-RA	0.40	0.00	Slightly down
	CG33129-RB	0.47	0.50	Slightly down
	CG33129-RC	0.58	0.38	Slightly down
	CG33129-RE	1.18	0.12	Up
CG33261	CG33261-RA	0.00	0.00	Slightly down
	CG33261-RB	0.91	0.92	Possibly absent
	CG33261-RC	0.00	0.07	Possibly absent
	CG33261-RD	17.48	0.00	Possibly absent
	CG33261-RE	0.76	0.00	Possibly absent
	CG33261-RF	1.68	0.00	Up
CG3413	CG3413-RA	1.18	0.78	Possibly absent
	CG3413-RB	1.37	0.12	Up
	CG3413-RC	1.03	0.00	Possibly absent

Continued on next page

Table S12 – continued from previous page

Gene	Transcript	alpha	beta	Call
	CG3413-RD	0.73	0.09	Slightly down
CG3671	CG3671-RA	0.66	0.46	Slightly down
	CG3671-RB	1.32	0.36	Up
	CG3671-RC	1.29	0.18	Up
CG3777	CG3777-RA	0.99	0.91	Unchanged
	CG3777-RB	0.85	0.09	Possibly absent
	CG3777-RC	1.78	0.00	Up
CG4376	CG4376-RA	1.09	0.78	Unchanged
	CG4376-RB	1.75	0.22	Up
	CG4376-RC	1.06	0.00	Possibly absent
CG4452	CG4452-RA	0.82	0.70	Slightly down
	CG4452-RB	1.84	0.29	Up
	CG4452-RC	0.52	0.01	Slightly down
CG4898	CG4898-RA	1.17	0.79	Up
	CG4898-RB	0.51	0.00	Slightly down
	CG4898-RC	0.97	0.00	Possibly absent
	CG4898-RD	1.24	0.00	Possibly absent
	CG4898-RE	0.93	0.00	Possibly absent
	CG4898-RF	0.81	0.01	Possibly absent
	CG4898-RH	1.35	0.17	Up
	CG4898-RI	0.65	0.02	Possibly absent
	CG4898-RJ	0.87	0.00	Possibly absent
	CG4898-RK	0.91	0.00	Possibly absent
	CG4898-RL	2.05	0.00	Possibly absent
CG6143	CG6143-RA	2.48	0.27	Up
	CG6143-RB	1.40	0.00	Up
	CG6143-RC	0.95	0.73	Unchanged
CG6391	CG6391-RA	0.44	0.24	Slightly down
	CG6391-RB	1.42	0.65	Up
	CG6391-RC	0.86	0.11	Possibly absent
CG6835	CG6835-RA	1.17	0.43	Possibly absent
	CG6835-RC	1.60	0.24	Up
	CG6835-RD	0.00	0.33	Slightly down
CG6946	CG6946-RA	0.64	0.73	Slightly down
	CG6946-RB	1.03	0.00	Possibly absent
	CG6946-RC	3.36	0.27	Up
CG7283	CG7283-RA	1.67	0.90	Unchanged
	CG7283-RB	4.33	0.10	Up
	CG7283-RC	0.87	0.00	Possibly absent
CG7852	CG7852-RA	1.43	1.00	Unchanged
	CG7852-RB	1.12	0.00	Possibly absent
	CG7852-RC	1.96	0.00	Up
CG8291	CG8291-RA	1.22	0.98	Up
	CG8291-RB	0.69	0.02	Slightly down
	CG8291-RC	0.96	0.00	Possibly absent
CG8874	CG8874-RA	0.78	0.00	Possibly absent
	CG8874-RB	1.53	0.03	Up
	CG8874-RC	1.24	0.00	Possibly absent
	CG8874-RD	1.28	0.96	Unchanged
CG8920	CG8920-RA	1.30	1.00	Up
	CG8920-RB	0.59	0.00	Slightly down
	CG8920-RC	1.01	0.00	Unchanged
