# Supplementary material for: Genome-Wide Identification of Alternative Splice Forms Down-Regulated by Nonsense-Mediated mRNA Decay in Drosophila
Source: PLoS Genet. 2009 Jun 19;5(6):e1000525. doi: 10.1371/journal.pgen.1000525 (PMC2689934; doi:10.1371/journal.pgen.1000525)
Supplement: Text S1 — Supplementary results and methods. (0.08 MB PDF) [file pgen.1000525.s035.pdf]

Genome-wide identification of alternative splice forms down-regulated by nonsense-mediated mRNA decay in *Drosophila*

Supplementary Text

Supplementary Results: *upf2* knockdown

As noted in the manuscript, a knockdown of *upf2* was performed but our analysis indicates that the knockdown was less effective. In the following the key results from the knockdown are described. The analysis pipeline is exactly as in the analysis of the *upf1* knockdown in the manuscript.

The number of genes successfully deconvolved were approximately the same as for the *upf1* knockdown; see Supplementary Table S5 for precise numbers. We found 18 genes with one or more NMD affected isoforms using the stringent p-value cutoff and 81 genes using the less stringent cutoff; see Supplementary Tables S6 and S8 for a detailed list.

Focusing on the stringent set of NMD targets for *upf1* and *upf2*, we found that the overlap between the two sets of targets was modest, only 10 genes. Due to the lower-efficiency knockdown of *upf2*, we expect smaller changes in isoform levels, causing a subset of the *upf1*-affected genes to be classified as NMD-affected. However, the 8 genes classified as NMD-affected only in the *upf2* knockdown were unexpected. A closer examination of the genes called affected by NMD only in the *upf2* experiment explains the difference. In short, 6 of the 8 genes show the NMD affected isoform being up-regulated in both *upf1* and *upf2*, but are labeled not affected by NMD in *upf1* due to the behaviour of the other isoforms. Our methods do not attempt to provide an exhaustive list of NMD targets; it is thus unsurprising that the *upf2* set is not a strict subset of the *upf1* set.

For six genes, the *upf1* data broadly follow the *upf2* story: the isoform we label as an NMD target in the *upf2* data is also clearly up-regulated upon knockdown of *upf1*. One of the six genes is found in the *upf1* loose set, so this is identified as a valid *upf1* target, albeit at a less stringent cutoff. In the other 5 cases, some other isoforms of the gene show a slightly different pattern, resulting in an isoform being labeled as "possibly absent" (often falling just on the other side of the p-value cutoff). This is a category that essentially consists of two broad groups: absent isoforms as well as unchanging isoforms where there is no positive evidence for their presence.

There is only one *upf2*-affected gene, CG3321, that does not seem to be affected by the *upf1* knockdown – overall the gene is slightly down-regulated and there is no evidence for differential expression between the two isoforms. Finally, the data for one gene are labeled as inconsistent – this could be due to bad data or due to having the wrong gene structure.

We analyzed the correlation between isoform features and NMD status for both the *upf1* affected genes separately (discussed in the manuscript) and for the union of the *upf1* and *upf2* affected genes, with approximately the same results.

Supplementary results: Coding constraints

Four of the NMD-affected genes have short dual-coding regions in which the same sequence is translated in two reading frames. We calculated the dN/dS of the dual-coding regions between *D. melanogaster* and *D. ananassae*

using PAL2NAL. Note that dN/dS is not intended for this sort of analysis, because the estimation of changes assumes the sequence encodes a protein in one frame, not two. "Normal" constraints on one frame would mean that the dN/dS in the shifted frame is almost certainly extremely high.

gene	dN/dS NMD	nontarget
glo	99.000	0.001
robo	99.000	0.343
CG4452	9.024	0.049
CG9413	0.233	0.674

We also analyzed the codon bias in the NMD-target and nontarget reading frames of the 27 NMD-affected genes with early stop codons. In most genes in our analysis, the two isoforms (target and nontarget) share much of their coding sequence in the same frame; the isoform-specific sequences are short. Using χ^2 tests, we find little statistically significant difference between the nontarget and NMD-target reading frames: only glutamine has a significant skew in its codon usage, with the nontarget mRNAs showing a much stronger preference for the preferred CAG codon. A comparison of both the nontarget mRNAs and the NMD-target mRNAs to a background distribution of codon usage from all RefSeq genes was more informative. The nontarget mRNAs had a significant difference in codon usage for 9 amino acids, in all cases skewed more towards the preferred codon. The NMD-target mRNAs had a significant difference for only 2 amino acids, in both cases showing less preference for the preferred codon. We believe that this shows that the productive reading frames are optimized for translation efficiency, and the unproductive reading frames are not as optimized. (The genes in this set include some that are transcribed and translated at high rates, such as ribosomal protein genes, so we are not surprised that they are more optimized relative to the overall codon bias.)

Supplementary Methods: CDS reannotation

We developed two methods for annotating the CDS of each transcript of an alternatively spliced gene.

I. Canonical transcript based on NMD status

We assume that the full-length protein is encoded by the longest ORF found in any NMD nontarget transcript from a given gene. We call the transcript with this longest ORF the canonical transcript, and the start of its CDS is the canonical start. Transcripts without the canonical CDS are compared to the canonical transcript(s). If they contain sequence upstream of the canonical start position that is not found in any of the canonical transcripts, then the first ORF beginning within this novel sequence is annotated as the CDS. Otherwise, the first ORF starting at or downstream of the canonical start codon position is annotated as the CDS.

For a given gene, the algorithm is as follows:

1. Identify the NMD nontarget isoform, L, with the longest ORF, l, and call the start of this ORF l_s.
2. For each isoform of this gene aside from L, identify any sequence upstream of l_s that is found in this isoform but not in L.
3. For each isoform, choose the CDS in this priority:
   (a) the first ORF starting within isoform-specific sequence upstream of l_s,
   (b) the first ORF starting at or after l_s.

II. Canonical transcript based on overall longest ORF

In the absence of data about NMD, we assume that the full-length protein is encoded by the longest ORF found in any transcript of the gene. A difficult-to-resolve situation occurs when multiple transcripts include the same longest ORF but differ in their sequence 5' of that ORF. The distinct 5' sequences might introduce different upstream start codons and uORFs that are differentially subject to translation and NMD. It is clear that the ribosome will sometimes skip upstream AUGs, but we cannot predict, *ab initio*, which uORF is ignored. In this scenario, lacking a way to make a biologically informed choice, we annotate the uORF of each transcript as its CDS and realize that the result is unlikely to reflect the biological reality.

For a given gene, the full algorithm is as follows:

1. Identify the longest ORF, l, out of all isoforms.
2. Collect all isoforms with an ORF that starts at the start of l (l_s) and ends at the end of l (l_e) as set L.
3. For each transcript in L, identify any sequence upstream of l_s that is found in this transcript but not in all transcripts in L.
4. For each transcript not in L, identify any sequence upstream of l_s that is found in this transcript but not in any transcript in L.
5. For each transcript, choose the CDS in this priority:
   (a) the first ORF starting within transcript-specific sequence upstream of l_s, as identified in 3 and 4 above,
   (b) the first ORF starting at or after l_s.

Full *D. melanogaster* chromosome sequences, including gene annotations from FlyBase 5.0, were downloaded from NCBI GenBank. We chose to use the newest FlyBase data, rather than the version used to construct the array, so that the most up-to-date gene structure information was included. Sequences were obtained for all NMD-target and nontarget isoforms of 41 of the 45 NMD-affected genes identified by the strict cutoff. The IDs of two of these genes (CG31305, CG33175) had changed; we used older versions of the chromosome files to obtain the sequences of these genes. The remaining four genes (CG1623, CG18009, CG2152, CG31237) were excluded because the newest FlyBase release was missing one or more of the transcripts identified as NMD-target or nontarget on our array. Similarly, 181 of the 189 genes in the less stringent *upf1*-affected set existed with both NMD-target and nontarget isoforms in the GenBank chromosome files. The IDs of five of these genes (CG31305, CG32140, CG33008, CG33085, CG33184) had changed and we used the original FlyBase sequence versions. Eight genes (CG2152, CG2225, CG5785, CG10901, CG13923, CG31237, CG33054, CR32885) were excluded because at least one transcript was missing in the newest version.

All possible ORFs were computed and a CDS was chosen for each transcript by each of the two algorithms described above. Isoforms that were not detected on the array were excluded from the analysis.

Supplementary methods: Feature calculation

Features of the genes in the NMD-affected sets were computed from the 5' UTR, CDS, and 3' UTR sequences of each NMD-target and nontarget mRNA, as annotated by the second method described above. The features were:

1. number of introns in transcript
2. length of 5' UTR
3. number of introns in 5' UTR
4. number of AUG (start) codons in 5' UTR

5. length of longest ORF in 5' UTR
6. fraction of A nucleotides in 5' UTR
7. length of longest A-rich region in 5' UTR
8. length of CDS
9. number of introns in CDS
10. length of 3' UTR
11. number of introns in 3' UTR
12. length of longest ORF in 3' UTR
13. fraction of A nucleotides in 3' UTR
14. length of longest A-rich region in 3' UTR
15. distance from stop codon to final intron

Further comments on the supplementary material

Additional figure information

In the suuplementary material we have a number of MA plots. In an MA plot, we plot the difference $M = y - x$ versus the average $A = (y + x)/2$. Here x is the $\log_2$ intensity for one array and y is the $\log_2$ intensity for another array. M is thus an estimate of the fold change and A is an estimate of the average expression level. For replicates, an MA plot ought to be concentrated along the line $M = 0$ and the variance of the M values should not depend on A. In addition to the MA plot we plot a lowess curve which is essentially a smoothed running average.

Some of the scatterplots have been jittered. This means that a small amount of random noise has been added to each (x, y)-value, to prevent overplotting.
